# Gallium/Indium-Promoted [4 + 2] Annulation of 2‑Aminobenzonitriles with Alkynes and Dimerization of 2‑Aminobenzonitriles: One-Pot Access to 4‑Aminoquinolines and 4‑Amino-2-Arylquinazolines

**DOI:** 10.1021/acsomega.6c01857

**Published:** 2026-05-28

**Authors:** Norio Sakai, Mio Ishii, Shuji Yamauchi, Yohei Ogiwara, Kento Ishida

**Affiliations:** Department of Pure and Applied Chemistry, Faculty of Science and Technology, Tokyo University of Science (RIKADAI), Noda, Chiba 278-8510, Japan

## Abstract

Aminoquinolines are key structural motifs in many biologically active substances. Herein, we report that the [4 + 2] annulation reactions of 2-aminobenzonitriles with nonactivated aryl/alkyl alkynes are performed in the presence of a group 13 Lewis acid (GaCl_3_ or InBr_3_) to produce a variety of 4-amino-2-substituted quinolines in moderate to good yields. This cycloaddition reaction was applicable to various terminal alkynes containing a halogen atom, an ester moiety, and a silyl group, and internal alkynes. Notably, the use of GaCl_3_ effectively promoted the dimerization of 2-aminobenzonitriles, leading to the preparation of 4-amino-2-arylquinazoline derivatives in good yields.

## Graphical abstract

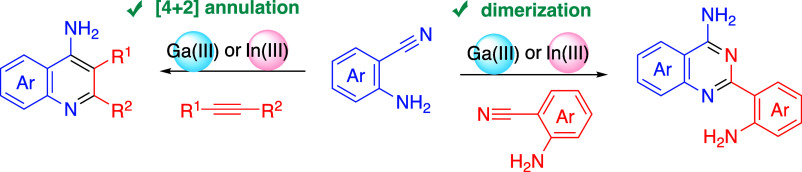

## Introduction

Aminoquinolines, particularly 4-aminoquinolines, are some of the most useful nitrogen-containing heterocycles and are common structural motifs in many biologically active substances, such as antimalarial drugs and antitumor agents.^1^ Consequently, many direct intermolecular approaches to the 4-aminoquinoline skeleton have been developed.^2,3^ In particular, annulation reactions that generate 4-aminoquinoline derivatives via the coupling of 2-aminobenzonitrile with suitable carbonyl derivatives as ketones and amides, or with alkynes, have been extensively investigated. For instance, in a stepwise approach performed using a carbonyl compound, the couplings of 2-aminobenzenitriles with ketones (or amides) in the presence of a Brønsted acid or a dehydrating agent generated imine intermediates that were subsequently subjected to base- or acid-promoted cyclization to produce 4-aminoquinoline derivatives (Scheme 1a).^1d,4^ Additionally, during a one-pot synthesis, Lewis acids, such as TiCl_4_,^5^ CuBTC,^6^ ZnCl_2_,^7^ BF_3_OEt_2_,^1b^ AlCl_3_,^8^ InCl_3_,^8^ and SnCl_4_,^9^ or protic acids^9^ promoted the coupling of 2-aminobenzenitriles with suitable ketones^10^ to yield 4-aminoquinoline derivatives (Scheme 1b). In these examples, each acid promotor played a dual role, acting as both condensation agent between the 2-aminobenzonitriles and carbonyl compounds, and as a cyclization promoter for the in situ-generated intermediates.

**Scheme 1:**
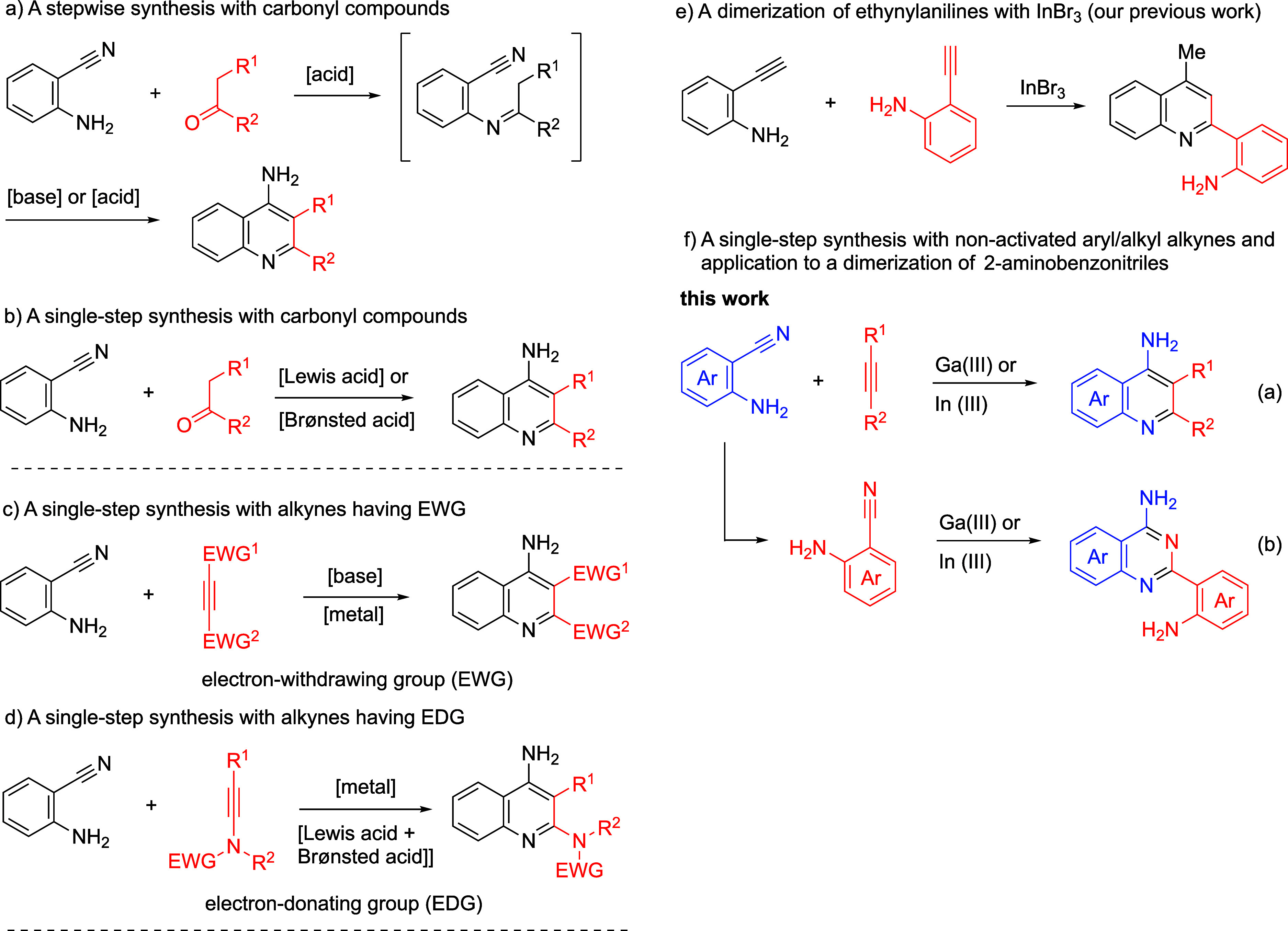
Diverse Approaches to 4-Aminoquinolines via the Couplings of 2-Aminobenzonitriles with Suitable Carbon Sources

Another skillful approach to this useful nitrogen-containing skeleton is the base- and metal-promoted coupling of 2-aminobenzonitriles with electron-deficient alkynes to produce 4-aminoquinolines (Scheme 1c).^11^ Additionally, the metal-catalyzed coupling of 2-aminobenzonitriles with ynamides bearing an electron-donating group (EDG), which behave as electron-rich alkynes yielded substituted 4-aminoquinolines (Scheme 1d). In these examples, single metal catalysts, such as ZnCl_2_ ^12^ and (Ph_3_P)­AuNTf_2_,^13^ a dual-metal catalyst that composed of Cu­(OAc)_2_ and Zn­(OTf)_2_,^14^ and a Me_3_SiOTf/TfOH system^15^ have been employed.

Although these approaches can incorporate various types of alkynes to produce valuable 4-aminoquinoline derivatives, most alkynes employed to date have been limited to activated alkynes containing an electron-withdrawing group and a sulfonamide group. Consequently, direct reactions between 2-aminobenzonitriles and simple aryl or alkyl alkynes in the presence of a Lewis acid are relatively unknown.

In this context, our group previously reported the indium­(III)-promoted dimerization of 2-ethynylanilines to generate 4-methylquinoline derivatives (Scheme 1e).^16^ It was found that selective activation of the alkyne moiety by a relatively soft Lewis acid, a trivalent indium compound, through π-interaction was the key step during dimerization.^17^ The current study therefore, focuses on the design of a straightforward route to 4-aminoquinoline frameworks via the [4 + 2] annulation of 2-aminobenzonitriles with simple alkynes in the presence of group 13 Lewis acid (Scheme 1f­(a)). Both gallium­(III)- and indium­(III)-promoted reactions are evaluated for the single-step synthesis of 4-methylquinoline derivatives. Moreover, the application of this Lewis acid-promoted dimerization of 2-aminobenzonitriles is demonstrated in the direct synthesis of useful 4-amino-2-arylquinazoline derivatives (Scheme 1f­(b)).

## Results and Discussion

Initially, the reaction conditions were optimized using 2-aminobenzonitrile (**1a**) and 1-hexyne (**2**) as model substrates (Table 1). When the reaction of **1a** with **2** (1.1 equiv) was performed in the presence of GaCl_3_ (1 equiv) in toluene at 80 °C for 1 h, the desired 4-amino-2-butylquinoline (**3a**) was obtained in 42% yield (entry 1). The structure of **3a** was confirmed by NMR spectroscopy and mass spectrometry. Since the side chain (C_4_) of **3a** can adopt a substructure equivalent that of a biologically active compound, Tacrine (9-amino-1,2,3,4-tetrahydroacridine), quinoline **3a** was used as a model compound.^18^ The use of 1,4-dioxane (dioxane), which enhanced the solubility of GaCl_3_ increased the yield of **3a** to 62%, while 1,2-dichloroethane (1,2-DCE) produced **3a** in 69% yield (entries 2 and 3). In contrast, the use of 0.5 equiv of GaCl_3_ lowered the yield of **3a** to 34% (entry 4). This is presumed to be due to a decrease in the catalytic activity of the gallium Lewis acid caused by the coordination of the basic amino group. Aluminum compounds as AlCl_3_ and AlBr_3_ were also examined; however, they failed to promote cyclization (entries 5 and 6). Then, the use of indium compounds as InCl_3_, InBr_3_, and InI_3_ led to decrease the yields (entries 7–9). The reaction performed using InBr_3_ in *o*-dichlorobenzene (*o*-DCB) at 120 °C produced quinoline **3a** in 72% yield (entry 10). Moreover, when the mixture of **1a** and **2** was treated with InBr_3_ (1 equiv) in toluene at 120 °C, the desired annulation proceeded cleanly to afford quinoline **3a** in 80% isolated yield (entry 11). Additionally, as a control experiment, the reaction of **1a** with **2** was carried out in the absence of a Lewis acid catalyst. As expected, the annulation reaction did not proceed, and the starting materials were recovered (entries 12 and 13).

**Table 1:**
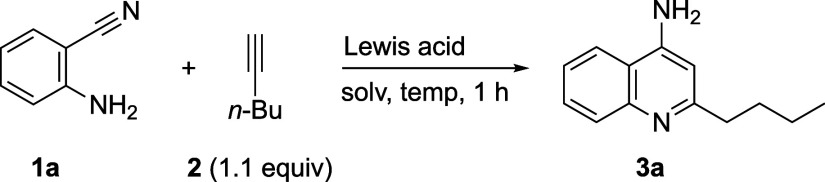
Optimization of Reaction Conditions^a^

entry	Lewis acid (1 equiv)	solvent	temp (°C)	GC yield of **3a** (%)
1	GaCl_3_	toluene	80	42
2	GaCl_3_	dioxane^b^	100	62
3	GaCl_3_	1,2-DCE^c^	80	71 (69)^d^
4	GaCl_3_ ^e^	1,2-DCE	80	34
5	AlCl_3_	1,2-DCE	80	ND^f^
6	AlBr_3_	1,2-DCE	80	ND^f^
7	InCl_3_	1,2-DCE	80	36
8	InBr_3_	1,2-DCE	80	50
9	InI_3_	1,2-DCE	80	47
10	InBr_3_	*o*-DCB^g^	120	72
11	InBr_3_	toluene	120	(80)^d^
12	----	toluene	120	NR
13	----	1,2-DCE	80	NR

a Standard conditions: **1a** (0.5 mmol), **2** (0.55 mmol), Lewis acid (0.5 mmol), solvent (0.8 mL), 80 °C (bath temperature), 1 h.

b 1,4-Dioxane.

c 1,2-Dichloroethane.

d Isolated yield.

e GaCl_3_ (0.5 equiv).

f ND = not detected.

g *o*-Dichlorobenzene.

With the optimal conditions in hand for reactions involving GaCl_3_ in 1,2-DCE (Method A) and InBr_3_ in toluene (Method B), the substrate scope was investigated using 2-aminobenzonitriles **1b**–**1i** (Scheme 2). Initially, the annulation reactions were performed using methyl-substituted 2-aminobenzonitriles **1b**–**1e** and 1-hexyne (**2**) were examined in the presence of GaCl_3_ or InBr_3_. In all cases, the desired annulation reactions occurred to produce the corresponding 2-butyl-substituted 4-aminoquinoline derivatives **3b**–**3e** in low to good yields. The results suggest that InBr_3_ tends to be more effective than GaCl_3_. Interestingly, when the reaction using 2-amino-4-methylbenzonitrile (**1c**) was treated with GaCl_3_ at 80̊C in 1,2-DCE, the desired 4-aminoquinoline derivative **3c** along with the formation of a trace amount of 2-(2-amino-4-methylphenyl)-4-amino-7-methylquinazoline (**4**) was observed via the dimerization of **1c** (see Scheme 6). Additionally, for the reaction of 2-amino-6-methylbenzonitrile (**1d**) performed in the presence of GaCl_3_ and InBr_3_, the corresponding stereoisomer **3d′** was obtained along with target **3d**. Similarly, when 2-amino-2-methylbenzonitrile (**1e**) were treated with GaCl_3_ and InBr_3_, the expected quinoline **3e** was formed in 48% and 83% yields, respectively. Only when using InBr_3_, the formation of a trace amount of stereoisomer **3e′** was observed. When the [4 + 2] annulation with 2-aminobezonitrile **1f** bearing two methoxy groups was then treated with InBr_3_, the corresponding stereoisomers **3f** and **3f′** was obtained. In contrast, when the reactions of 2-amino-6-chlorobenzonitrile (**1g**) performed in the presence of GaCl_3_ and InBr_3_, the desired 4-amino-2-butyl-5-chloroquinoline (**3g**) was obtained in yields of 6% and 24%, respectively. In this case, the formation of a stereoisomer like compounds **3d′** and **3f′** shown above was not observed. In similar, 2-amino-4-chlorobenzonitrile (**1h**) underwent cyclization to afford 4-amino-2-butyl-5-chloroquinoline (**3h**) in good yield. Although the annulation of **1i** bearing a strongly electron-withdrawing nitro group did not proceed in the presence of GaCl_3_, the reaction using InBr_3_ successfully proceeded to produce the desired quinoline **3i**. Thus, when a similar annulation reaction with substrate **1j** bearing a trifluoromethyl group was examined in the presence of InBr_3_, the desired [4 + 2] annulation proceeded to afford the corresponding quinoline **3j** in good yield along with trace amount of stereoisomer **3j′**. From the above results, it is suggested that when an electron-donating group is bonded onto the aryl group of 2-aminobenzonitriles, there is a tendency for stereoisomers to be formed.

**Scheme 2:**
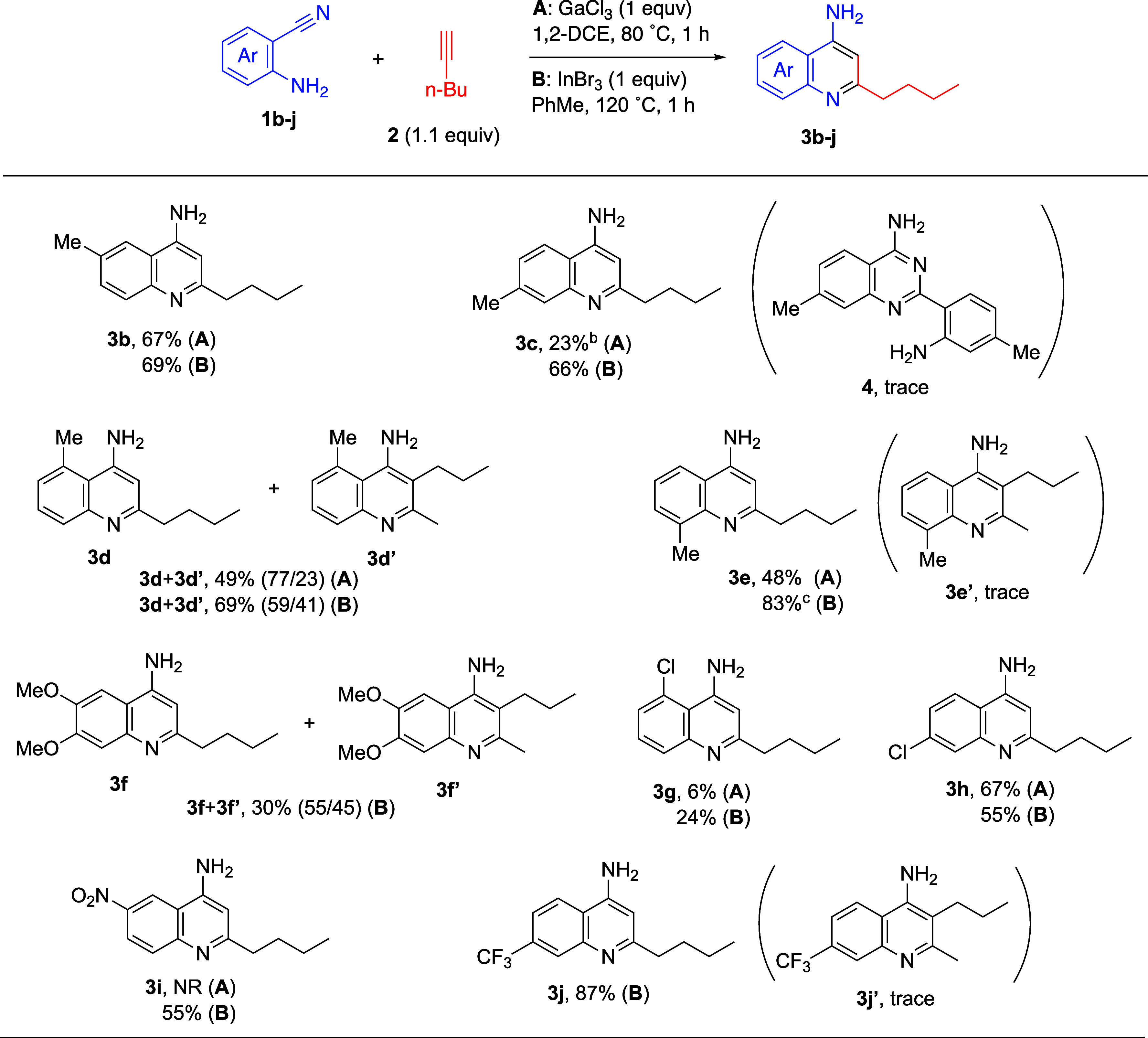
Substrate Scope of the 2-Aminobenzonitriles^a^

a Isolated yield.
b Formation of 4-aminoquinazoline derivative **4** was observed.
c Formation of a trace amount of stereoisomer **3e′** was observed.

The substrate scope was subsequently investigated for the reactions of 2-aminobenzonitrile (**1a**) with different aryl alkynes (Scheme 3). Initially, the reaction of **1a** with phenylacetylene (**5a**) was performed using GaCl_3_ in 1,2-DCE, affording the corresponding quinoline **6aa** in 71% yield. The reaction of **1a** with arylalkynes bearing a tolyl group also produced the desired 4-aminoquinolines **6ab** and **6ac** in good yields. Moreover, substrates bearing an electron-withdrawing substituent, such as a trifluoromethyl and a fluorine group, yielded the corresponding 4-aminoquinolines **6ad–6af** in high yields. To our delight, this annulation of **1a** was successfully extended to aryl alkyne **5g** bearing a thiophen ring, producing the expected quinoline derivative **6ag** in moderate yield. This reaction was also attempted using InBr_3_ in toluene; however, no improvement in yield was observed. In contrast, the reaction of **1a** with 3-ethynylpyridine (**5h**) failed to produce the desired quinoline **6ah** in the presence of either GaCl_3_ or InBr_3_, potentially due to the deactivation of these Lewis acids by the strongly basic pyridine ring. As another example, the coupling of 2-amino-6-fluorobenzonitrile (**1k**) with phenylacetylene (**5a**) was examined using InBr_3_, producing the corresponding quinoline derivative **6ka** in 86% yield. X-ray crystallographic analysis of **6ka** unambiguously confirmed the preparation of a 4-amino-2-arylquinoline skeleton.^19^

**Scheme 3:**
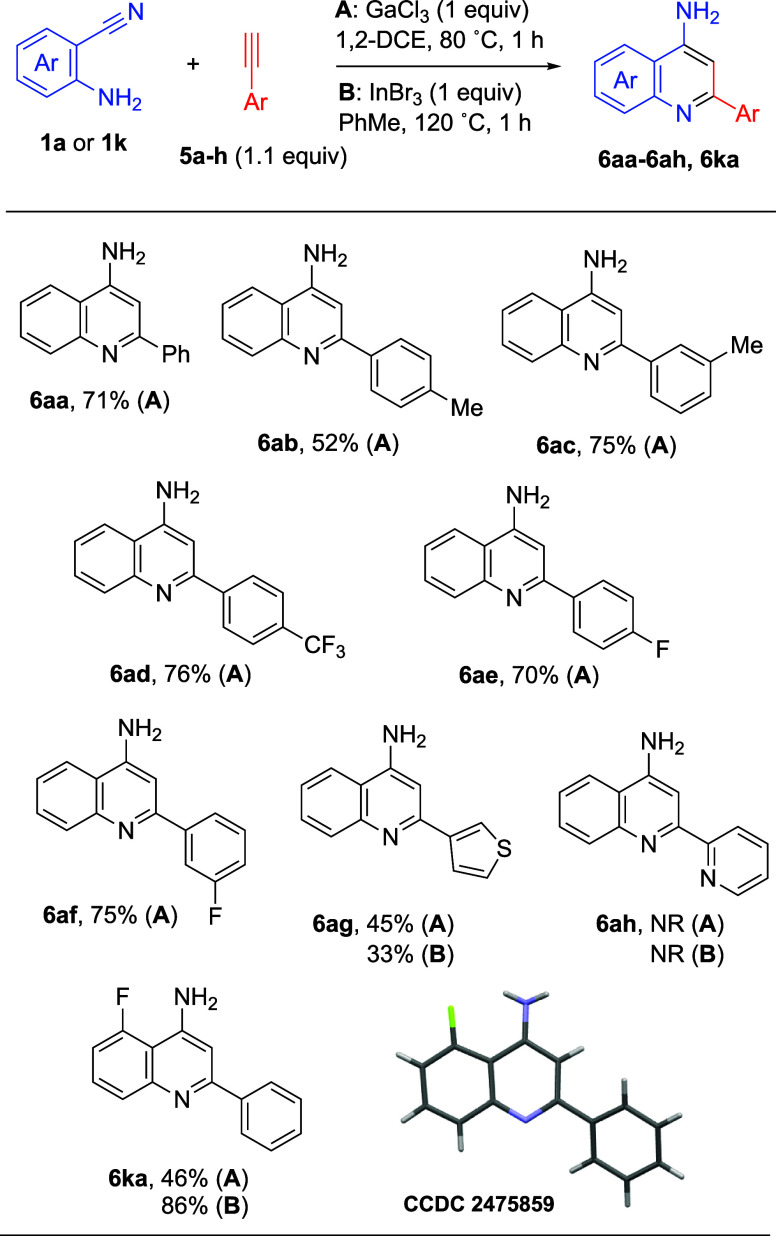
Substrate Scope of the Aryl Alkynes^a^

a Isolated yield

Subsequently, the coupling reactions of 2-aminobenzonitrile (**1a**) with various alkynes **7a**–**7e** were examined under the optimal conditions involving GaCl_3_ and InBr_3_ (Scheme 4). Initially, the reaction of **1a** with 6-chloro-1-hexyne (**7a**) was performed in GaCl_3_ in 1,2-DCE at 80 °C, contrary to expectations, producing 4-amino-3-(4-chlorobutyl)-2-methylquinoline (**8**) in 23% yield. This result was comparable to the above results that yielded stereoisomers **3d′** and **3f′** (see Scheme 2). There is no clear explanation of this result at this stage. Under the optimal conditions with GaCl_3_, the coupling of **1a** with ethyl propionate (**7b**) yielded 3-substituted quinoline **9** in a regioselective manner in 91% yield. Notably, the conventional studies have reported that the coupling of **1a** with electron-deficient alkynes proceeded only in the presence of typical bases.^11b,11c^ Thus, assuming that 2-aminobenzonitrile itself functions as a base, the annulation of **1a** with **7b** was attempted in the absence of a Lewis acid. However, the desired quinoline derivative **9** was not produced. This result demonstrated that GaCl_3_ is essential for the activating reaction substrates. Interestingly, the coupling reaction between **1a** and trimethylsilylacetylene (**7c**) in the presence of GaCl_3_ produced a 2,3-unsubstituted quinoline, 4-aminoquinoline (**10**), in good yield. Furthermore, as an example of an internal aliphatic alkyne, 6-dodecyne (**6e**) was reacted in the presence of GaCl_3_ affording the expected 2,3-disubstituted quinoline derivative **11**; using InBr_3_ improved the yield slightly to 60%. Moreover, the reaction between **1a** and an internal aromatic alkyne, diphenylacetylene (**6f**), in InBr_3_/toluene at 120 °C produced the desired 4-amino-2,3-diphenylquinoline (**12**) in moderate yield. Under the optimal conditions involving InBr_3_, employment of 1-octyne (**7f**), which have a longer carbon chain than 1-hexyne, afforded 4-amino-2-hexylquinoline (**13**) was obtained as the sole product. Moreover, when the reaction of **1a** with an unsymmetrical alkyne, 2-hexyne (**7g**), was carried out using InBr_3_, complete consumption of the starting material **1a** was observed; however, 2-methyl-substituted quinoline derivative **14** was obtained in only 8% yield along with a trace amount of regioisomer **14′**. At this stage, there is no clear explanation for the reason for the low yield of the annulation with an unsymmetrical alkyne. Unfortunately, 2-amino-3-cyanothionphene (**1l**) was not applicable to this type of [4 + 2] annulation, and most of the starting material was recovered.

**Scheme 4:**
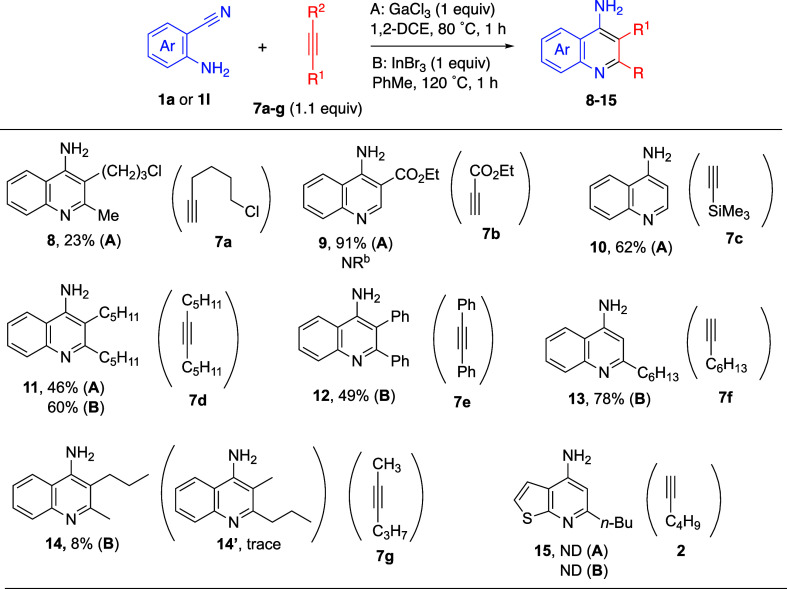
Substrate Scope of Various Alkynes^a^

a Isolated yield.
b Performed in the absence of a Lewis acid.

Subsequently, the developed method was applied to the large-scale synthesis of 4-aminoquinoline derivative **6aa** (Scheme 5). Specifically, the coupling of **1a** with phenylacetylene (**5a**) in the presence of InBr_3_ afforded **6aa** in 67% yield (1.25 g) after purification by column chromatography.

**Scheme 5:**
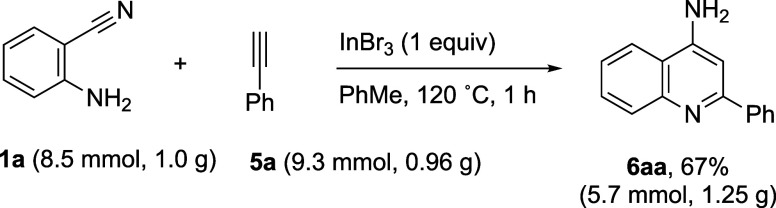
Gram-Scale Synthesis of Quinoline **6aa**

Notably, during the synthesis of 4-amino-2-butyl-7-methylquinoline (**3d**) via the GaCl_3_-promoted coupling of 2-amino-4-methylbenzonitrile (**1d**) with alkyne **2a**, a small amount of quinazoline derivative **4** was observed (see Scheme 2). Although this type of Lewis acid-promoted dimerization of 2-aminobenzonitriles has not been studied extensively, our group previously reported the related dimerization, in which InBr_3_ effectively promoted the dimerization of 2-ethynylanilines to afford the corresponding quinoline derivatives.^16^ Considering the importance of the quinazoline-4-amine/imine skeleton in various biologically active substances,^20^ this result motivated the development of a unique and direct preparation of valuable quinazoline derivatives.^21^ Following optimization of the dimerization conditions, it was found that the protocol for 4-amino-2-arylquinoline synthesis could be extended to the direct construction of a quinazoline skeleton. Specifically, the treatment of **1a** with GaCl_3_ in toluene at 120 °C for 20 h promoted the desired dimerization, yielding the corresponding quinazoline derivative, 2-(2-aminophenyl)-4-aminoquinazoline (**16**), in 64% isolated yield; the use of InBr_3_ led to a slightly reduced yield (Scheme 6a). Similarly, the reaction of 2-amino-4-methylbenzonitrile (**1d**) and 2-amino-4-chlorobenzonitrile (**1h**) under identical GaCl_3_-promoted conditions produced 2-(2-amino-4-methylphenyl)-4-amino-7-methylquinazoline (**4**) and 2-(2-amino-4-chlorophenyl)-4-amino-7-chloroquinazoline (**17**) in yields of 53% and 69% yields, respectively (Scheme 6b,c). These results indicate that the electronic effect of the aromatic substituent did not influence the reaction outcome. The structures of these quinazoline skeletons were unequivocally established by X-ray crystallographic analysis using a single crystal of quinazoline **4**.^22^

**Scheme 6:**
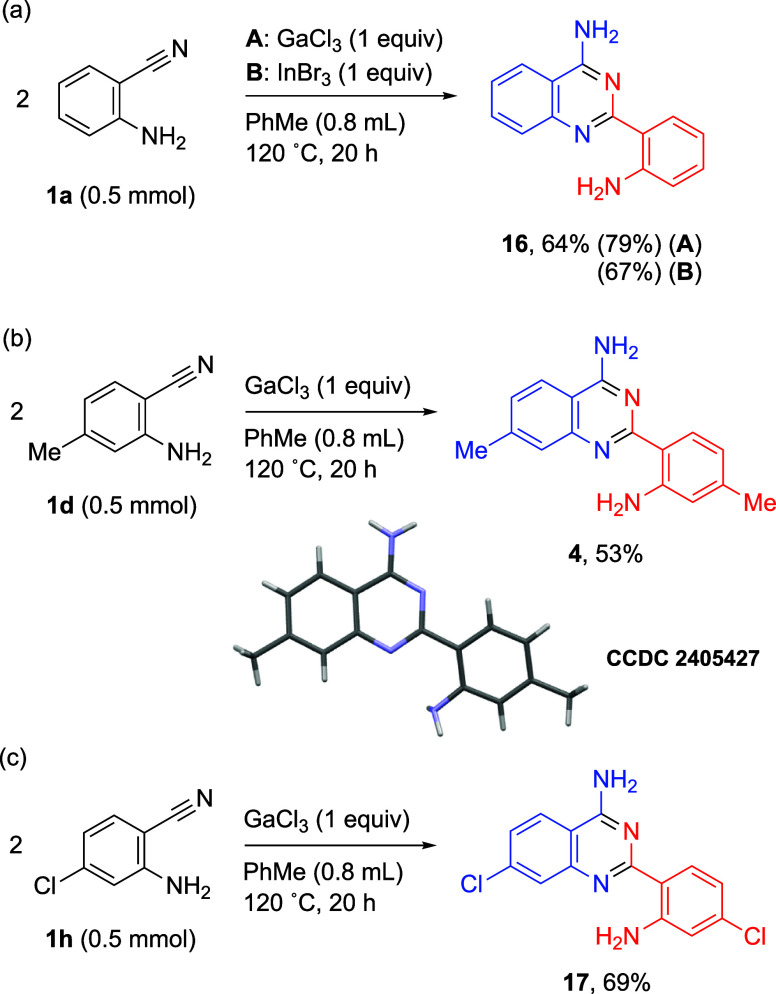
Group 13 Metal-Promoted Dimerization of 2-Aminobenzonitriles to Generate 4-Amino-2-arylquinazolines^a^

a Isolated (NMR) yield.

Based on our previous work,^23^ in which it was found that the indium-catalyzed hydroamination of aryl or alkyl alkynes with anilines/hydrazones proceeded in a Markovnikov-type manner, it was considered that the annulation reactions between 2-aminobenzonitriles and alkynes would proceed similarly (Scheme 7). As propose in Scheme 7a, the reaction of aryl alkynes with 2-aminobenzonitrile begins with Lewis acid-activated Markovnikov-type hydroamination, affording the corresponding enamine intermediate **A**. Subsequently, enamine intermediate **A** cyclizes to form cycloadduct **B**, followed by aromatization to produce 4-amino-2-arylquinoline **C**. For 2-aminobenzonitriles bearing an electron-donating group (Scheme 7b), in most cases, an initial hydroamination proceeds in a manner analogous to that shown in Scheme 7a to generate enamine intermediate **D**, then enamine **D** cyclizes to yield 4-amino-2-alkylquinoline **E** as a major product. However, a portion of enamine **D** bearing an electron-rich aryl group tends to isomerize to a more stable disubstituted enamine intermediate **G** through imine intermediate **F**, followed by a rapid cyclization of enamine **G** to give 4-amino-3-alkyl-2-methylquinoline **H** as a minor product. Notably, this type of isomerization has also been observed in the presence of titanium and zinc catalysts.^24^ For the dimerization of 2-aminobenzonitrile (Scheme 7c), considering on our previous work on the indium-promoted dimerization of 2-ethynylanilines,^16^ it was proposed that an amino group of 2-aminobenzonitrile adds to a nitrile moiety of another 2-aminobenzonitrile, forming cycloadduct **I**. Subsequently, aromatization of **I** affords 4-amino-2-arylquinazoline **J**.

**Scheme 7:**
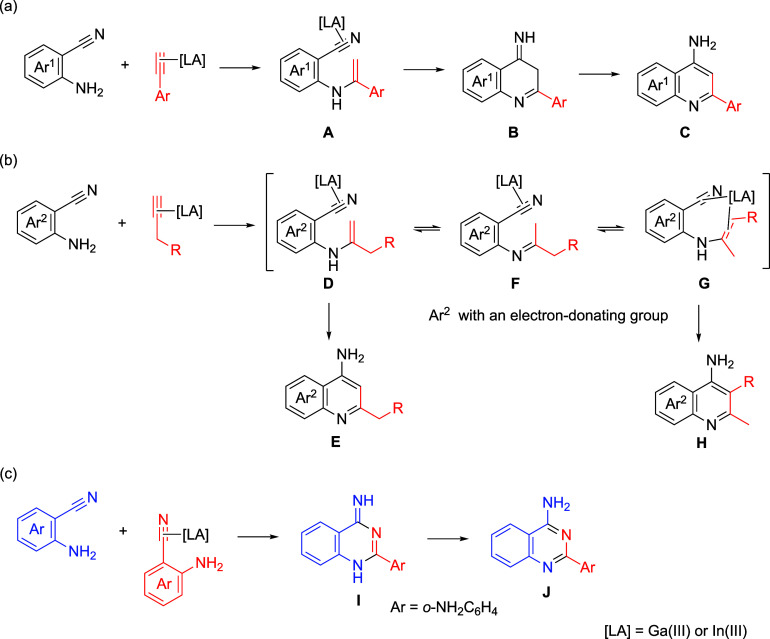
Plausible Reaction Mechanisms

## Conclusion

This study demonstrated that the gallium- or indium-promoted coupling of 2-aminobenzonitriles with various terminal or internal alkynes produced a variety of 4-amino-2-substituted quinoline derivatives in moderate to good yields. It was also confirmed that GaCl_3_ effectively promoted the dimerization of 2-aminobenzonitriles, leading to the direct production of 4-amino-2-arylquinazoline derivatives in good yields. Notably, the group 13 Lewis acids investigated in this work (i.e., GaCl_3_ and InBr_3_) effectively activated the triple bonds of both a simple alkyne and a 2-aminobenzonitrile, facilitating subsequent annulation to produce valuable nitrogen-containing heterocycles in practical yields. Ongoing efforts in our group are focused on the coupling reaction using other unsaturated compounds to further survey in this reaction system.

## Experimental Section

### General Experimental Methods

All reactions were carried out under a nitrogen atmosphere, unless otherwise noted. Toluene and dioxane were distilled over Na-benzophenone prior to use. 1,2-DCE was dried over calcium chloride and calcium hydride prior to subsequent distillation. *O*-DCB was dried over calcium chloride and calcium hydride prior to distillation under reduced pressure. 2-Aminobenzonitriles **1a**–**k**, 2-amino-3-cyanothionphene (**1l**), 1-hexyne (**2**), alkynes **5a**–**h**, and alkynes **7a**–**g**, were commercially available and were purified according to conventional distillation and recrystallization protocols prior to use. The Lewis acids (AlCl_3_, AlBr_3_, GaCl_3_, InCl_3_, InBr_3_, and InI_3_) were also commercially available and were used without further purification. Column chromatography was performed using silica gel. The ^1^H NMR spectra were measured at 500 or 400 MHz using tetramethylsilane as an internal standard (0.00 ppm) or using the center peak of dimethyl sulfoxide (2.5 ppm). The ^13^C­{^1^H} NMR spectra were measured at 125 or 100 MHz using the center peak of chloroform (77.0 ppm) or dimethyl sulfoxide (39.5 ppm) as a reference. The ^19^F NMR spectra were measured at 470 or 376 MHz using trifluorotoluene as an external standard (−62.6 ppm) as a reference. High-resolution mass spectra (HRMS) were performed using NBA (3-nitrobenzyl alcohol) as the matrix.

### General Procedure for the Synthesis of 4-Aminoquinoline Derivatives (Method **A**)

To a screw-capped vial with a stir bar in a N_2_-filled glovebox, GaCl_3_ (88.0 mg, 0.500 mmol) was added. The vial was then sealed and removed from the glovebox, and 1,2-DCE (0.8 mL), a 2-aminobenzonitrile derivative (0.5 mmol), and an alkyne (0.55 mmol) were successively added. The mixture was heated at 80 °C (bath temperature) for 1 h prior to quenching with a saturated aqueous Na_2_CO_3_ solution (3 mL). Subsequently, the aqueous layer was extracted with chloroform (5 × 5 mL). The combined organic layer was dried over anhydrous Na_2_SO_4_, filtered, and evaporated under reduced pressure. The crude material was purified via silica gel column chromatography (eluent: hexane/ethyl acetate/triethylamine = 4:1:0.1) or preparative thin-layer chromatography to yield the corresponding 4-aminoquinoline derivative.

### General Procedure for the Synthesis of 4-Aminoquinoline Derivatives (Method **B**)

To a screw-capped vial with a stir bar in a N_2_-filled glovebox, InBr_3_ (177 mg, 0.500 mmol) was added. The vial was then sealed and removed from the glovebox, and toluene (0.8 mL), a 2-aminobenzonitrile derivative (0.5 mmol), and an alkyne (0.55 mmol) were successively added. The mixture was heated at 120 °C (bath temperature) for 1 h prior to quenching with a saturated aqueous Na_2_CO_3_ solution (3 mL). Subsequently, the aqueous layer was extracted with chloroform (5 × 5 mL). The combined organic layer was dried over anhydrous Na_2_SO_4_, filtered, and evaporated under reduced pressure. The crude material was purified via silica gel column chromatography (eluent: hexane/ethyl acetate/triethylamine = 5:5:0.2) or preparative thin-layer chromatography to yield the corresponding 4-aminoquinoline derivative.

#### 4-Amino-2-butylquinoline (**3a**)

General procedure was followed with 2-aminobenzonitrile (59.1 mg, 0.500 mmol) and 1-hexyne (45.2 mg, 0.550 mmol) for 1 h. Silica gel column chromatography (eluent: hexane/EtOAc/NEt_3_ = 4:1:0.1) afforded **3a** as a brown oil (Method **A**: 69.0 mg, 69%; Method **B**: 80.0 mg, 80%); ^1^H NMR (400 MHz, CDCl_3_): δ 7.96 (d, *J* = 8.2 Hz, 1H), 7.73 (d, *J* = 8.2 Hz, 1H), 7.58 (t, *J* = 8.2 Hz, 1H), 7.34 (t, *J* = 8.2 Hz, 1H), 6.48 (s, 1H), 5.01 (br s, 2H), 2.79 (t, *J* = 7.8 Hz, 2H), 1.73 (quint, *J* = 7.8 Hz, 2H), 1.39 (sext, *J* = 7.4 Hz, 2H), 0.91 (t, *J* = 7.4 Hz, 3H); ^13^C­{^1^H} NMR (100 MHz, CDCl_3_): δ 163.3, 149.9, 148.4, 129.2, 128.7, 123.9, 120.2, 117.5, 103.1, 38.8, 32.1, 22.6, 13.9; HRMS (FAB-Magnetic Sector): calcd for C_13_H_17_N_2_ [M + H]^+^, 201.1392; found, 201.1409.

#### 4-Amino-2-butyl-6-methylquinoline (**3b**)

General procedure was followed with 2-amino-5-methylbenzonitrile (66.1 mg, 0.500 mmol) and 1-hexyne (45.2 mg, 0.550 mmol) for 1 h. Silica gel column chromatography (eluent: hexane/EtOAc/NEt_3_ = 4:1:0.1) afforded **3b** as a pale yellow solid (Method **A**: 72.2 mg, 67%; Method **B**: 73.6 mg, 69%): ^1^H NMR (400 MHz, CDCl_3_): δ 7.83 (d, *J* = 8.8 Hz, 1H), 7.47–7.41 (m, 2H), 6.46 (s, 1H), 4.76 (br s, 2H), 2.78 (t, *J* = 7.8 Hz, 2H), 2.46 (s, 3H), 1.76–1.68 (m, 2H), 1.45–1.35 (m, 2H), 0.92 (t, *J* = 7.3 Hz, 3H); ^13^C­{^1^H} NMR (100 MHz, CDCl_3_): δ 162.5, 149.1, 147.0, 133.6, 131.3, 128.7, 119.1, 117.4, 103.3, 38.9, 32.2, 22.7, 21.5, 13.9; HRMS (FAB-Magnetic Sector): calcd for C_14_H_19_N_2_ [M + H]^+^, 215.1548; found, 215.1552.

#### 4-Amino-2-butyl-7-methylquinoline (**3c**)

General procedure was followed with 2-amino-4-methylbenzonitrile (66.1 mg, 0.500 mmol) and 1-hexyne (45.2 mg, 0.550 mmol) for 1 h. Silica gel column chromatography (eluent: hexane/EtOAc/NEt_3_ = 5:5:0.2) afforded **3c** as a white solid (Method **A**: 24.6 mg, 23%; Method **B**: 70.8 mg, 66%); ^1^H NMR (400 MHz, CDCl_3_): δ 7.74 (s, 1H), 7.60 (d, *J* = 8.7 Hz, 1H), 7.20 (dd, *J* = 8.7, 1.4 Hz, 1H), 6.44 (s, 1H), 4.73­(br s, 2H), 2.78 (t, *J* = 7.8 Hz, 2H), 2.49 (s, 3H), 1.73 (quint, *J* = 7.8 Hz, 2H), 1.40 (sext, *J* = 7.8 Hz, 2H), 0.93 (t, *J* = 7.3 Hz, 3H); ^13^C­{^1^H} NMR (100 MHz, CDCl_3_): δ 163.4, 149.5, 148.8, 139.3, 128.2, 126.0, 119.7, 115.4, 102.8, 39.0, 32.2, 22.7, 21.6, 14.0; HRMS (FAB-Magnetic Sector): calcd for C_14_H_19_N_2_ [M + H]^+^, 215.1548; found, 215.1547.

#### 4-Amino-2-butyl-5-methylquinoline (**3d**) and 4-Amino-3-propyl-2,5-methylquinoline (**3d′**)

General procedure was followed with 2-amino-6-methylbenzonitrile (66.1 mg, 0.500 mmol) and 1-hexyne (45.2 mg, 0.550 mmol) for 1 h. Silica gel column chromatography (eluent: hexane/EtOAc/NEt_3_ = 5:5:0.2) afforded **3d** + **3d′** as a white solid (Method **A**: 53 mg, 49% (**3d**/**3d′** = 77/23); Method **B**: 73.4 mg, 69%, **3d**/**3d′** = 59/41); For **3d**; ^1^H NMR (400 MHz, CDCl_3_): δ 7.77 (d, *J* = 8.7 Hz, 1H), 7.39 (t, *J* = 7.5 Hz, 1H), 7.04 (d, *J* = 6.9 Hz, 1H), 6.38 (s, 1H), 4.95 (br s, 2H), 2.89 (s, 3H), 2.74 (t, *J* = 7.8 Hz, 2H), 1.75–1.67 (m, 2H), 1.40 (td, *J* = 14.9, 7.5 Hz, 2H), 0.93 (t, *J* = 7.3 Hz, 3H); For **3d′**; ^1^H NMR (400 MHz, CDCl_3_): δ 7.72 (d, *J* = 8 Hz, 1H), 7.34 (t, *J* = 6.6 Hz, 1H), 7.03 (s, 1H), 4.88 (br s, 2H), 2.99 (s, 3H), 2.87 (s, 2H), 2.56 (t, *J* = 8 Hz, 1H), 1.62–1.53 (m, 2H), 1.04 (t, *J* = 7.4 Hz, 3H); ^13^C­{^1^H} NMR (100 MHz, CDCl_3_): δ 162.4, 157.1, 152.3, 150.7, 148.7, 148.3, 133.0, 132.4, 128.5, 127.7, 127.6, 127.5, 127.4, 127.0, 118.2, 117.9, 114.4, 104.9, 38.5, 32.0, 29.2, 24.7, 24.5, 23.6, 22.6, 20.6, 14.4, 13.9; HRMS (FAB-Magnetic Sector): calcd for C_14_H_19_N_2_ [M + H]^+^, 215.1548; found, 215.1570.

#### 4-Amino-2-butyl-8-methylquinoline (**3e**)

General procedure was followed with 2-amino-3-methylbenzonitrile (66.1 mg, 0.500 mmol) and 1-hexyne (45.2 mg, 0.550 mmol) for 1 h. Silica gel column chromatography (eluent: hexane/EtOAc/NEt_3_ = 4:1:0.1) afforded **3e** as a white solid (Method **A**: 51.4 mg, 48%; Method **B**: 88.3 mg, 83% (**3e** + **3e′**)): ^1^H NMR (500 MHz, CDCl_3_): δ 7.57 (d, *J* = 8.2 Hz, 1H), 7.47 (d, *J* = 6.9 Hz, 1H), 7.27 (dd, *J* = 8.2, 7.3 Hz, 1H), 6.51 (s, 1H), 4.58 (br s, 2H), 2.84 (t, *J* = 7.8 Hz, 2H), 2.77 (s, 3H), 1.82–1.74 (m, 2H), 1.43 (sext, *J* = 7.5 Hz, 2H), 0.96 (t, *J* = 7.3 Hz, 3H); ^13^C­{^1^H} NMR (125 MHz, CDCl_3_): δ 162.2, 149.6, 147.7, 137.2, 129.5, 123.6, 117.8, 117.5, 103.6, 39.1, 31.9, 22.7, 18.7, 14.2; HRMS (FAB-Magnetic Sector): calcd for C_14_H_19_N_2_ [M + H]^+^, 215.1548; found, 215.1544.

#### 4-Amino-2-butyl-6,7-dimethoxyquinoline (**3f**) and 4-Amino-2-methyl-3-propyl-6,7-dimethoxyquinoline (**3f′**)

General procedure was followed with 2-amino-4,5-dimethoxybenzonitrile (98 mg, 0.50 mmol) and 1-hexyne (45.2 mg, 0.550 mmol) for 1 h. Silica gel column chromatography (eluent: hexane/EtOAc/NEt_3_ = 4:1:0.1) afforded **3f** + **3f′** as a white solid (Method **B**: 38.6 mg, 30%, **3f**/**3f′** = 55/45): For **3f**; ^1^H NMR (400 MHz, CDCl_3_): δ 7.36 (s, 1H), 7.16 (s, 1H), 6.43 (s, 1H), 5.56 (br s, 2H), 3.95 (s, 3H), 3.94 (s, 3H), 2.74 (t, *J* = 8 Hz, 2H), 1.73–1.65 (m, 2H), 1.36 (sext, 2H, *J* = 7.2 Hz), 0.89 (t, 3H, *J* = 7.2 Hz); For **3f′**; ^1^H NMR (400 MHz, CDCl_3_): δ 7.32 (s, 1H), 7.16 (s, 1H), 5.45 (br s, 2H), 3.98 (s, 3H x 2, overlap), 2.60 (s, 3H), 2.58–2.54 (m, 2H), 1.61–1.51 (m, 2H), 1.05 (s, 3H, *J* = 7.2 Hz); ^13^C NMR (100 MHz, CDCl_3_): δ 159.5, 153.4, 152.5, 152.0, 150.5, 148.2, 148.0, 147.6, 143.0, 140.0, 112.8, 111.4, 111.2, 106.0, 105.3, 102.2, 99.9, 99.8, 56.22 (x2), 56.16, 56.0, 37.4, 32.0, 28.8, 22.5, 21.8, 21.1, 14.3, 13.8; HRMS (FAB-Magnetic Sector): calcd for C_15_H_21_N_2_O_2_ [M + H]^+^, 261.1603; found, 261.1603.

#### 4-Amino-2-butyl-5-chloroquinoline (**3g**)

General procedure was followed with 2-amino-6-chlorobenzonitrile (76.3 mg, 0.500 mmol) and 1-hexyne (45.2 mg, 0.550 mmol) for 1 h. Silica gel column chromatography (eluent: hexane/EtOAc/NEt_3_ = 4:1:0.1) afforded **3g** as a white solid (Method **A**: 7 mg, 6%; Method **B**: 28.1 mg, 24%): ^1^H NMR (500 MHz, CDCl_3_): δ 7.81 (d, *J* = 8.8 Hz, 1H), 7.39 (t, *J* = 8.8 Hz, 1H), 7.26 (d, *J* = 8.8 Hz, 1H), 6.41 (s, 1H), 5.78 (br s, 2H), 2.73 (t, *J* = 7.6 Hz, 2H), 1.74–1.66 (m, 2H), 1.38 (sext, *J* = 7.6 Hz, 2H), 0.94 (t, *J* = 7.6 Hz, 3H); ^13^C­{^1^H} NMR (125 MHz, CDCl_3_): δ 163.3, 151.2, 151.1, 128.9, 128.7, 128.5, 126.4, 115.1, 105.2, 38.4, 31.9, 22.6, 14.0; HRMS (FAB-Magnetic Sector): calcd for C_13_H_16_ClN_2_ [M + H]^+^, 235.1002; found, 235.1026.

#### 4-Amino-2-butyl-7-chloroquinoline (**3h**)

General procedure was followed with 2-amino-4-chlorobenzonitrile (76.3 mg, 0.500 mmol) and 1-hexyne (45.2 mg, 0.550 mmol) for 1 h. Silica gel column chromatography (eluent: hexane/EtOAc/NEt_3_ = 4:1:0.1) afforded **3h** as a white solid (Method **A**: 78.4 mg, 67%; Method **B**: 64.2 mg, 55%); ^1^H NMR (500 MHz, DMSO-*d*_6_): δ 8.14 (d, *J* = 8.6 Hz, 1H), 7.70 (d, *J* = 2.3 Hz, 1H), 7.34 (dd, *J* = 8.6, 2.3 Hz, 1H), 6.85 (br s, 2H), 6.47 (s, 1H), 2.66 (t, *J* = 7.7 Hz, 2H), 1.66 (quint, *J* = 7.6 Hz, 2H), 1.34 (sext, *J* = 7.3 Hz, 2H), 0.91 (t, *J* = 7.2 Hz, 3H); ^13^C­{^1^H} NMR (125 MHz, DMSO-*d*_6_): δ 163.5, 151.9, 149.0, 133.5, 126.6, 124.4, 123.1, 115.9, 101.9, 37.9, 31.3, 22.0, 13.9; HRMS (FAB-Magnetic Sector): calcd for C_13_H_16_ClN_2_ [M + H]^+^, 235.1002; found, 235.0998.

#### 4-Amino-2-butyl-6-nitroquinoline (**3i**)

General procedure for method **B** was followed with 2-amino-5-nitrobenzonitrile (81.6 mg, 0.500 mmol) and 1-hexyne (45.2 mg, 0.550 mmol) for 1 h. Silica gel column chromatography (eluent: hexane/EtOAc/NEt_3_ = 5:5:0.2) afforded **3i** as an orange oil (66.8 mg, 55%); ^1^H NMR (400 MHz, CDCl_3_): δ 8.85 (d, *J* = 2.7 Hz, 1H), 8.33 (dd, *J* = 9.1, 2.3 Hz, 1H), 7.98 (d, *J* = 9.6 Hz, 1H), 6.62 (s, 1H), 5.37 (br s, 2H), 2.83 (t, *J* = 7.8 Hz, 2H), 1.75 (quint, *J* = 7.7 Hz, 2H), 1.41 (sext, *J* = 7.4 Hz, 2H), 0.94 (t, *J* = 7.3 Hz, 3H); ^13^C­{^1^H} NMR (100 MHz, CDCl_3_): δ 167.5, 151.6, 151.2, 143.1, 130.2, 122.8, 118.4, 116.2, 104.3, 39.0, 31.8, 22.6, 13.9; HRMS (FAB-Magnetic Sector): calcd for C_13_H_16_N_3_O_2_ [M + H]^+^, 246.1243; found, 246.1243.

#### 4-Amino-2-butyl-7-trifluoromethylquinoline (**3j**)

General procedure for method **B** was followed with 2-amino-5-trifluoromethylbenzonitrile (163 mg, 1.00 mmol) and 1-hexyne (90 mg, 1.1 mmol) for 1 h. Silica gel column chromatography (eluent: hexane/EtOAc/NEt_3_ = 5:5:0.2) afforded **3j** as a yellow solid (229 mg, 87%); ^1^H NMR (400 MHz, CDCl_3_): δ 8.26 (s, 1H), 7.83 (d, *J* = 8.4 Hz, 1H), 7.50 (d, *J* = 8.4 Hz, 1H), 6.58 (s, 1H), 5.05 (br s, 2H), 2.81 (t, *J* = 8.0 Hz, 2H), 1.75–1.71 (m, 2H), 1.42–1.37 (m, 2H), 0.92 (t, *J* = 7.6 Hz, 3H); ^13^C­{^1^H} NMR (100 MHz, CDCl_3_): δ 165.0, 149.7, 147.7, 130.9 (*J*_C–F_ = 32.2 Hz), 126.6, 123.9 (*J*_C–F_ = 271.5 Hz), 121.5, 119.3 (*J*_C–F_ = 16.3 Hz), 104.5, 38.9, 31.9, 22.5, 13.8; ^19^F NMR (376 MHz, CDCl_3_): δ −62.6; HRMS (FAB-Magnetic Sector): calcd for C_14_H_16_F_3_N_2_ [M + H]^+^, 269.1266; found, 269.1261.

#### Gram-Scale Synthesis of **6aa**

To a screw-capped tube (30 mL) with a stir bar in a N_2_-filled glovebox, toluene (17 mL), ethynylbenzene (0.96 g, 9.3 mmol), InBr_3_ (3.0 g, 8.5 mmol), and 2-aminobenzonitrile (1.0 g, 8.5 mmol) were successively added. The tube was then sealed and removed from the glovebox, and heated at 120 °C (bath temperature) for 1 h. After this time, the reaction mixture was quenched using a saturated aqueous Na_2_CO_3_ solution (30 mL). The aqueous layer was extracted with chloroform (3 × 30 mL), and the combined organic layer was dried over Na_2_SO_4_, filtered, evaporated under reduced pressure. The crude material was purified by recrystallization to yield 2-phenyl-4-aminoquinoline (**6aa**) as a white solid (1.25 g, 67%).

#### 4-Amino-2-phenylquinoline (**6aa**)^1d^

General procedure for method **A** was followed with 2-aminobenzonitrile (59.1 mg, 0.500 mmol) and ethynylbenzene (56.2 mg, 0.550 mmol) for 1 h. Silica gel column chromatography (eluent: hexane/EtOAc/NEt_3_ = 4:1:0.1) afforded **6aa** as a white solid (Method **A**: 78.1 mg, 71%); ^1^H NMR (500 MHz, DMSO-*d*_6_): δ 8.16 (d, *J* = 7.4 Hz, 1H), 8.09 (dd, *J* = 7.4, 7.4 Hz, 2H), 7.84 (d, *J* = 7.4 Hz, 1H), 7.61 (t, *J* = 7.4 Hz, 1H), 7.49 (t, *J* = 7.4 Hz, 2H), 7.44–7.41 (m, 1H), 7.38 (t, *J* = 7.4 Hz, 1H), 7.12 (s, 1H), 6.85 (br s, 2H); ^13^C­{^1^H} NMR (125 MHz, DMSO-*d*_6_): δ 156.2, 152.4, 148.8, 140.0, 129.3, 129.2, 128.8, 128.5, 126.8, 123.5, 122.2, 117.8, 99.1; HRMS (FAB-Magnetic Sector): calcd for C_15_H_13_N_2_ [M + H]^+^, 221.1079; found, 221.1073.

#### 4-Amino-2-(4-methylphenyl)­quinoline (**6ab**)

General procedure for method **A** was followed with 2-aminobenzonitrile (59.1 mg, 0.500 mmol) and 1-ethynyl-4-methylbenzene (63.9 mg, 0.550 mmol) for 1 h. Silica gel column chromatography (eluent: hexane/EtOAc/NEt_3_ = 4:1:0.1) afforded **6ab** as a white solid (60.9 mg, 52%): ^1^H NMR (500 MHz, CDCl_3_): δ 8.07 (d, *J* = 8.0 Hz, 1H), 7.97 (d, *J* = 8.0 Hz, 2H), 7.75 (d, *J* = 7.6 Hz, 1H), 7.66 (t, *J* = 6.8 Hz, 1H), 7.43 (t, *J* = 6.8 Hz, 1H), 7.29 (d, *J* = 7.6 Hz, 2H), 7.06 (s, 1H), 4.73 (br s, 2H), 2.42 (s, 3H); ^13^C­{^1^H} NMR (100 MHz, CDCl_3_): δ 158.0, 149.9, 149.1, 139.0, 137.4, 130.1, 129.5, 129.3, 127.3, 124.5, 119.9, 117.9, 101.4, 21.3; HRMS (FAB-Magnetic Sector): calcd for C_16_H_15_N_2_ [M + H]^+^, 235.1235; found, 235.1261.

#### 4-Amino-2-(3-methylphenyl)­quinoline (**6ac**)

General procedure for method **A** was followed with 2-aminobenzonitrile (59.1 mg, 0.500 mmol) and 1-ethynyl-3-methylbenzene (63.9 mg, 0.550 mmol) for 1 h. Silica gel column chromatography (eluent: hexane/EtOAc/NEt_3_ = 4:1:0.1) afforded **6ac** as a white solid (87.8 mg, 75%); ^1^H NMR (500 MHz, DMSO-*d*_6_): δ 8.20 (d, *J* = 8.4 Hz, 1H), 7.96 (s, 1H), 7.89 (dd, *J* = 8.4, 7.6 Hz, 2H), 7.66 (t, *J* = 7.6 Hz, 1H), 7.45–7.41 (m, 2H), 7.30 (d, *J* = 7.6 Hz, 1H), 7.16 (s, 1H), 6.84 (br s, 2H), 2.46 (s, 3H); ^13^C­{^1^H} NMR (125 MHz, DMSO-*d*_6_): δ 156.3, 152.3, 148.8, 139.9, 137.5, 129.4, 129.2, 129.1, 128.4, 127.4, 123.9, 123.4, 122.2, 117.8, 99.1, 21.2; HRMS (FAB-Magnetic Sector): C_16_H_15_N_2_ [M + H]^+^, 235.1235; found, 235.1256.

#### 4-Amino-2-(4-trifluoromethylphenyl)­quinoline (**6ad**)

General procedure for method **A** was followed with 2-aminobenzonitrile (59.1 mg, 0.500 mmol) and 1-ethynyl-4-(trifluoromethyl)­benzene (93.6 mg, 0.550 mmol) for 1 h. Silica gel column chromatography (eluent: hexane/EtOAc/NEt_3_ = 4:1:0.1) afforded **6ad** as a white solid (109.5 mg, 76%): ^1^H NMR (400 MHz, CDCl_3_): δ 8.18 (d, *J* = 8.2 Hz, 2H), 8.09 (d, *J* = 8.2 Hz, 1H), 7.79–7.68 (m, 4H), 7.48 (t, *J* = 8.9 Hz, 1H), 7.07 (d, *J* = 7.3 Hz, 1H), 4.82 (br s, 2H); ^13^C­{^1^H} NMR (100 MHz, CDCl_3_): δ 156.4, 150.3, 149.0, 143.6 (q, *J*_C–F_ = 1.4 Hz), 130.7 (q, *J*_C–F_ = 32.1 Hz), 130.3, 129.9, 127.7, 125.5 (q, *J*_C–F_ = 3.8 Hz), 125.1, 124.4 (q, *J*_C–F_ = 271 Hz), 119.9, 117.9, 101.3; ^19^F NMR (376 MHz, CDCl_3_): δ −62.4; HRMS (FAB-Magnetic Sector): calcd for C_16_H_12_F_3_N_2_ [M + H]^+^, 289.0953; found, 289.0979.

#### 4-Amino-2-(4-fluorophenyl)­quinoline (**6ae**)^25^

General procedure for method **A** was followed with 2-aminobenzonitrile (59.1 mg, 0.500 mmol) and 1-ethynyl-4-fluorobenzene (66.1 mg, 0.550 mmol) for 1 h. Silica gel column chromatography (eluent: hexane/EtOAc/NEt_3_ = 4:1:0.1) afforded **6ae** as a white solid (83.3 mg, 70%); ^1^H NMR (400 MHz, DMSO-*d*_6_): δ 8.17–8.11 (m, 3H), 7.83 (d, *J* = 8 Hz, 1H), 7.61 (t, *J* = 7.5 Hz, 1H), 7.37 (t, *J* = 7.5 Hz, 1H), 7.31 (t, *J* = 8.5 Hz, 2H), 7.09 (s, 1H), 6.89 (br s, 2H); ^13^C­{^1^H} NMR (100 MHz, DMSO-*d*_6_): δ 164.0 (d, *J*_C–F_ = 245.3 Hz), 156.9, 153.8, 148.7, 136.5 (d, *J*_C–F_ = 2.9 Hz), 131.3, 130.3 (d, *J*_C–F_ = 8.6 Hz), 128.8, 125.4, 122.8, 118.3, 116.5 (d, *J*_C–F_ = 21.1 Hz), 100.9; ^19^F NMR (376 MHz, CDCl_3_): δ −112.9; HRMS (FAB-Magnetic Sector): calcd for C_15_H_12_FN_2_ [M + H]^+^, 239.0985; found, 239.0989.

#### 4-Amino-2-(3-fluorophenyl)­quinoline (**6af**)

General procedure for method **A** was followed with 2-aminobenzonitrile (59.1 mg, 0.500 mmol) and 1-ethynyl-3-fluorobenzene (66.1 mg, 0.550 mmol) for 1 h. Silica gel column chromatography (eluent: hexane/EtOAc/NEt_3_ = 4:1:0.1) afforded **6af** as a white solid (89.3 mg, 75%); ^1^H NMR (500 MHz, DMSO-*d*_6_): δ 8.19 (d, *J* = 7.4 Hz, 1H), 7.91–7.89 (m, 2H), 7.86 (d, *J* = 7.4 Hz, 1H), 7.62 (t, *J* = 7.4 Hz, 1H), 7.52 (q, *J* = 7.4 Hz, 1H), 7.39 (t, *J* = 8.0 Hz, 1H), 7.27–7.23 (m, 1H), 7.15 (s, 1H), 6.92 (br s, 2H); ^13^C­{^1^H} NMR (125 MHz, DMSO-*d*_6_): δ 162.6 (d, *J*_C–F_ = 242.6 Hz), 154.7, 152.6, 148.7, 142.6 (d, *J*_C–F_ = 7.2 Hz), 130.5 (d, *J*_C–F_ = 8.4 Hz), 129.5, 129.2, 123.8, 122.7, 122.2, 117.9, 115.5 (d, *J*_C–F_ = 21.7 Hz), 113.3 (d, *J*_C–F_ = 22.9 Hz), 99.1; ^19^F NMR (470 MHz, CDCl_3_): δ −112.9; HRMS (FAB-Magnetic Sector): calcd for C_15_H_12_FN_2_ [M + H]^+^, 239.0985; found, 239.1008.

#### 4-Amino-2-(3-thienyl)­quinoline (**6ag**)

General procedure was followed with 2-aminobenzonitrile (59.1 mg, 0.500 mmol) and 3-ethynylthiophene (59.5 mg, 0.550 mmol) for 1 h. Silica gel column chromatography (eluent: hexane/EtOAc/NEt_3_ = 5:5:0.2) afforded **6ag** as a white solid (Method **A**: 50.9 mg, 45%; Method **B**: 37.3 mg, 33%): ^1^H NMR (400 MHz, CDCl_3_): δ 8.03 (d, *J* = 8.2 Hz, 1H), 7.92 (d, *J* = 1.8 Hz, 2H), 7.75 (d, *J* = 4.6 Hz, 1H), 7.70 (d, *J* = 8.2 Hz, 1H), 7.63 (t, *J* = 7.8 Hz, 1H), 7.40–7.37 (m, 2H), 6.93 (s, 1H), 4.78 (br s, 2H); ^13^C­{^1^H} NMR (100 MHz, CDCl_3_): δ 153.8, 149.9, 148.9, 143.0, 129.8, 129.5, 126.7, 125.9, 124.4, 119.9, 117.9, 101.3; HRMS (FAB-Magnetic Sector): calcd for C_13_H_11_N_2_S [M + H]^+^, 227.0643; found, 227.0643.

#### 4-Amino-5-fluoro-2-phenylquinoline (**6ka**)

General procedure for method **A** was followed with 2-amino-6-fluorobenzonitrile (74.9 mg, 0.500 mmol)­and ethynylbenzene (56.2 mg, 0.550 mmol) for 1 h. Silica gel column chromatography (eluent: hexane/EtOAc/NEt_3_ = 4:1:0.1) afforded **6ka** as a white solid (Method **A**: 54.5 mg, 46% Method **B** 102.9 mg, 86%); ^1^H NMR (500 MHz, CDCl_3_): δ 8.03 (d, *J* = 7.6 Hz, 2H), 8.09 (d, *J* = 8.8 Hz, 1H), 7.51–7.42 (m, 4H), 7.00 (d, *J* = 7.6 Hz, 1H), 6.92 (s, 1H, ArH), 5.39 (br s, 2H); ^13^C NMR­(125 MHz, CDCl_3_): δ 160.9, 158.6 (d, *J*_C–F_ = 288.0 Hz), 150.8 (d, *J*_C–F_ = 105.4 Hz), 139.7, 129.2, 128.9, 128.7, 128.6, 127.4, 125.9 (d, *J*_C–F_ = 3.9 Hz), 109.0, 108.9, 108.5, 108.5, 101.8; ^19^F NMR (470 MHz, CDCl_3_): δ −115.3, HRMS (FAB-Magnetic Sector): calcd for C_15_H_12_FN_2_ [M + H]^+^, 239.0885; found, 239.0989.

#### 4-Amino-3-(1-chloropropyl)-2-methylquinoline (**8**)

General procedure was followed with 2-aminobenzonitrile (59.1 mg, 0.500 mmol) and 6-chloro-1-hexyne (64.1 mg, 0.550 mmol) for 1 h. Silica gel column chromatography (eluent: hexane/EtOAc/NEt_3_ = 4:1:0.1) afforded **8** as a white solid (Method **A**: 27 mg, 23%); ^1^H NMR (500 MHz, CDCl_3_): δ 7.90 (d, *J* = 8.5 Hz, 1H), 7.70 (d, *J* = 8.5 Hz, 1H), 7.57 (dd, *J* = 8.5, 8.5 Hz, 1H), 7.40 (dd, *J* = 8.5, 8.5 Hz, 1H), 4.89 (br s, 2H), 3.67 (t, *J* = 6.0 Hz, 2H), 2.86 (t, *J* = 7.7 Hz, 2H), 2.66 (s, 3H), 2.08–2.02 (m, 2H); ^13^C­{^1^H}­NMR (125 MHz, CDCl_3_): δ 157.9, 146.6, 146.5, 128.7 (overlap), 124.3, 119.9, 117.6, 112.1, 45.2, 30.7, 24.0, 23.6; HRMS (FAB-Magnetic Sector): calcd for C_13_H_16_ClN_2_ [M + H]^+^, 235.1002; found, 235.1026.

#### Ethyl 4-Aminoquinoline-3-carboxylate (**9**)^3b^

General procedure was followed with 2-aminobenzonitrile (59.1 mg, 0.500 mmol) and ethyl propiolate (54 mg, 0.55 mmol) for 1 h. Silica gel column chromatography (eluent: hexane/EtOAc/NEt_3_ = 4:1:0.1) afforded **9** as a white solid (Method **A**: 98.3 mg, 91%); ^1^H NMR (500 MHz, DMSO-*d*_6_): δ 8.89 (s, 1H), 8.37 (d, *J* = 7.5 Hz, 1H), 8.35 (br s, 2H), 7.80 (d, *J* = 7.5 Hz, 1H), 7.73 (dd, *J* = 7.5, 7.5 Hz, 1H), 7.50 (dd, *J* = 7.5, 7.5 Hz, 1H), 4.32 (q, *J* = 7 Hz, 2H), 1.33 (t, *J* = 7 Hz, 3H); ^13^C­{^1^H} NMR (125 MHz, DMSO-*d*_6_): δ 167.5, 154.2, 151.2, 148.8, 131.5, 129.1, 125.2, 123.2, 118.1, 99.5, 60.2, 14.2; HRMS (FAB-Magnetic Sector): calcd for C_12_H_13_N_2_O_2_ [M + H]^+^, 217.0977; found, 217.0978.

#### 4-Aminoquinoline (**10**)^26^

General procedure was followed with 2-aminobenzonitrile (59.1 mg, 0.500 mmol) and trimethylsilylacetylene (54 mg, 0.55 mmol) for 1 h. Silica gel column chromatography (eluent: CHCl_3_/MeOH/NEt_3_ = 5:1:0.12) afforded **10** as a white solid (Method **A**: 44.3 mg, 62%; Method **B**: complex mixture); ^1^H NMR (400 MHz, CDCl_3_): δ 8.52 (d, *J* = 5.2 Hz, 1H), 8.00 (d, *J* = 8 Hz, 1H), 7.78 (d, *J* = 8 Hz, 1H), 7.65 (dd, *J* = 8, 8 Hz, 1H), 7.45 (dd, *J* = 8, 8 Hz, 1H), 6.59 (d, *J* = 5.2 Hz, 1H), 4.95 (br s, 2H); ^13^C­{^1^H} NMR (100 MHz, CDCl_3_): δ 150.6, 149.6, 148.7, 129.8, 129.4, 124.8, 120.2, 118.7, 103.7; HRMS (FAB-Magnetic Sector): calcd for C_9_H_9_N_2_ [M + H]^+^, 145.0766; found, 145.0768.

#### 4-Amino-2,3-dipentylquinoline (**11**)

General procedure was followed with 2-aminobenzonitrile (59.1 mg, 0.500 mmol) and 6-dodecyne (91.5 mg, 0.550 mmol) for 1 h. Silica gel column chromatography (eluent: hexane/EtOAc/NEt_3_ = 4:1:0.1) afforded **11** as a colorless oil (Method **A**: 65.3 mg, 46%; Method **B**: 84.6 mg, 60%); ^1^H NMR (400 MHz, CDCl_3_): δ 7.93 (d, *J* = 8 Hz, 1H), 7.68 (d, *J* = 8 Hz, 1H), 7.55 (dd, *J* = 8, 8 Hz, 1H), 7.35 (dd, *J* = 8, 8 Hz, 1H), 4.68 (br s, 2H), 2.91–2.87 (m, 2H), 2.68–2.63 (m, 2H), 1.76–1.71 (m, 2H), 1.62–1.55 (m, 2H), 1.48–1.33 (m, 8H), 1.01–0.89 (m, 6H); ^13^C­{^1^H} NMR (100 MHz, CDCl_3_): δ 161.9, 146.5, 146.2, 129.0, 128.2, 124.0, 119.8, 117.6, 113.8, 36.7, 32.2, 29.9, 28.2, 27.0, 22.6, 22.5, 14.0, 13.9 (overlap); HRMS (FAB-Magnetic Sector): calcd for C_19_H_29_N_2_ [M + H]^+^, 285.2331; found, 285.2336.

#### 4-Amino-2,3-diphenylquinoline (**12**)^27^

General procedure was followed with 2-aminobenzonitrile (59.1 mg, 0.500 mmol) and diphenylacetylene (98 mg, 0.55 mmol) for 1 h. Silica gel column chromatography (eluent: hexane/EtOAc/NEt_3_ = 5:5:0.2) afforded **12** as a colorless solid (Method **B**: 73.1 mg, 49%); ^1^H NMR (400 MHz, CDCl_3_): δ 8.11 (d, *J* = 9.2 Hz, 1H), 7.79 (d, *J* = 8.0 Hz, 1H), 7.69 (dd, *J* = 15.2, 1.2 Hz, 1H), 7.50 (dd, *J* = 15.2, 1.2 Hz, 1H), 7.36–7.25 (m, 5H), 7.21–7.15 (m, 5H), 4.75 (br s, 2H); ^13^C­{^1^H} NMR (100 MHz, CDCl_3_): δ 158.8, 147.5, 147.2, 141.2, 136.5, 131.1, 130.1, 129.7, 129.4, 129.0, 127.5, 127.4, 127.3, 125.0, 120.4, 117.4, 116.0; HRMS (FAB-Magnetic Sector): calcd for C_21_H_17_N_2_ [M + H]^+^, 297.1392; found, 297.1390.

#### 4-Amino-2-hexylquinoline (**13**)

General procedure was followed with 2-aminobenzonitrile (118 mg, 1.00 mmol) and 1-octyne (121 mg, 0.550 mmol) for 1 h. Silica gel column chromatography (eluent: hexane/EtOAc/NEt_3_ = 4:1:0.1) afforded **13** as a brown oil (Method **B**: 176 mg, 78%); ^1^H NMR (400 MHz, CDCl_3_): δ 7.96 (d, *J* = 7.8 Hz, 1H), 7.75 (d, *J* = 7.8 Hz, 1H), 7.59 (t, *J* = 6.8 Hz, 1H), 7.39 (t, *J* = 6.8 Hz, 1H), 6.52 (s, 1H), 4.89 (br s, 2H), 2.80 (t, *J* = 8 Hz, 2H), 1.76–1.72 (m, 2H), 1.45–1.39 (m, 2H), 1.35–1.30 (m, 4H), 0.86 (t, *J* = 7.4 Hz, 3H); ^13^C­{^1^H} NMR (100 MHz, CDCl_3_): δ 163.2, 149.9, 148.2, 129.4, 128.7, 124.1, 120.1, 117.5, 103.2, 39.1, 31.7, 30.0, 29.3, 22.5, 14.0; HRMS (FAB-Magnetic Sector): calcd for C_15_H_21_N_2_ [M + H]^+^, 229.1699; found, 229.1704.

#### 2-Methyl-3-propyl-4-aminoquinoline (**14**)

General procedure was followed with 2-aminobenzonitrile (118 mg, 1.00 mmol) and 2-hexyne (90 mg, 1.1 mmol) for 1 h. Silica gel column chromatography (eluent: hexane/EtOAc/NEt_3_ = 4:1:0.1) afforded **14** as a pale yellow solid (Method **B**: 15 mg, 8%); ^1^H NMR (400 MHz, CDCl_3_): δ 7.90 (d, *J* = 8 Hz, 1H), 7.69 (d, *J* = 8 Hz, 1H), 7.57 (dd, *J* = 7.2 Hz, 6 Hz, 1H), 7.38 (dd, *J* = 7.2 Hz, 6 Hz, 1H), 4.62 (br s, 2H), 2.69–2.65 (m, 2H), 2.67 (s, 3H), 1.63 (sext, *J* = 7.6 Hz, 2H), 1.09 (t, *J* = 7.6 Hz, 3H); ^13^C­{^1^H} NMR (100 MHz, CDCl_3_) d 158.3, 146.4, 140.0, 129.0, 128.4, 124.2, 119.7, 117.8, 114.1, 29.5, 23.9, 21.3, 14.5; HRMS (FAB-Magnetic Sector): *m*/*s* Calcd for C_13_H_17_N_2_ (M^+^+H): 201.1392; Found: 201.1391.

### General Procedure for the Synthesis of 4-Aminoquinazoline Derivatives (Method **A**)

To a screw-capped vial with a stir bar in a N_2_-filled glovebox, a 2-amino-4-methylbenzonitrile derivative (0.5 mmol), GaCl_3_ (88.4 mg, 0.500 mmol), and toluene (0.8 mL) were successively added. The vial was then sealed and removed from the glovebox, and the mixture was heated at 120 °C (bath temperature) for 20 h. After this time, the resulting mixture was quenched using a saturated aqueous Na_2_CO_3_ solution (5 mL), and the aqueous layer was extracted with chloroform (10 × 2 mL). The combined organic layer was dried over Na_2_SO_4_, filtered, and evaporated. The crude material was purified by silica gel column chromatography (eluent: hexane/ethyl acetate = 4:1) to yield the corresponding 4-aminoquinazoline derivative.

### General Procedure for the Synthesis of 4-Aminoquinazoline Derivatives (Method **B**)

To a screw-capped vial with a stir bar in a N_2_-filled glovebox, a 2-amino-4-methylbenzonitrile derivative (0.5 mmol), InBr_3_ (177 mg, 0.500 mmol), and toluene (0.8 mL) were successively added. The vial was then sealed and removed from the glovebox, and the mixture was then heated at 120 °C (bath temperature) for 20 h. After this time, the resulting mixture was quenched using a saturated aqueous Na_2_CO_3_ solution (5 mL), and the aqueous layer was extracted with chloroform (10 × 2 mL). The combined organic layer was dried over Na_2_SO_4_, filtered, and evaporated. The crude material was purified by silica gel column chromatography (eluent: hexane/ethyl acetate = 4:1) to yield the corresponding 4-aminoquinazoline derivative.

#### 4-Amino-2-(2-aminophenyl)­quinazoline (**16**)^28^

General procedure was followed with 2-aminobenzonitrile (59.1 mg, 0.500 mmol) for 20 h. Silica gel column chromatography (eluent: hexane/EtOAc/NEt_3_ = 4:1:0.1) afforded **16** as a colorless solid (Method **A**: 37.6 mg, 64%; Method **B**: 5.0 mg, 8%); ^1^H NMR (400 MHz, CDCl_3_): δ 8.44 (dd, *J* = 7.8, 1.6 Hz, 1H), 7.82 (d, *J* = 8.0 Hz, 1H), 7.70 (ddd, *J* = 8.2, 6.8, 1.2 Hz, 2H) 7.65 (d, *J* = 8.4 Hz, 1H), 7.37­(ddd, *J* = 8.2, 6.8, 1.2 Hz, 1H) 7.21 (ddd, *J* = 8.2, 6.8, 1.2 Hz, 1H) 6.77 (m, 2H), 6.49 (s, 2H), 5.74 (s, 2H); ^13^C­{^1^H} NMR (100 MHz, CDCl_3_): δ 162.1, 160.6, 149.9, 148.7, 133.1, 131.1, 130.9, 128.1, 125.4, 121.6, 119.7, 116.9, 116.7, 112.4; MS­(EI) *m*/*z* 236; HRMS (FAB-Magnetic Sector): calcd for C_14_H_13_N_4_ [M + H]^+^, 237.1140; found, 237.1141.

#### 4-Amino-2-(2-amino-4-methylphenyl)-7-methylquinazoline (**4**)

General procedure was followed with 2-amino-4-methylbenzonitrile (66.1 mg, 0.500 mmol) for 20 h. Silica gel column chromatography (eluent: hexane/EtOAc/NEt_3_ = 4:1:0.1) afforded **4** as a colorless solid (Method **A**: 35 mg, 53%; Method **B**: 36 mg, 53%); ^1^H NMR (400 MHz, CDCl_3_): δ 8.34 (d, *J* = 8 Hz, 1H), 7.62 (s, 1H), 7.59 (d, *J* = 8 Hz, 1H), 7.22 (d, *J* = 8 Hz, 1H), 6.58 (d, *J* = 8 Hz, 1H), 6.55 (s, 1H), 5.55 (br s, 2H), 2.51 (s, 3H), 2.29 (s, 3H); ^13^C­{^1^H} NMR (100 MHz, CDCl_3_): δ 162.3, 160.4, 150.2, 148.9, 143.9, 141.5, 131.0, 127.34, 127.26, 121.5, 118.1, 117.4, 117.2, 110.3, 22.0, 21.5; HRMS (FAB-Magnetic Sector): calcd for C_16_H_17_N_4_ [M + H]^+^, 265.1453; found, 265.1447.

#### 4-Amino-2-(2-amino-4-chlorophenyl)-7-chloroquinazoline (**17**)

General procedure was followed with 2-amino-4-chlorobenzonitrile (76.3 mg, 0.500 mmol) for 20 h. Silica gel column chromatography (eluent: hexane/EtOAc/NEt_3_ = 4:1:0.1) afforded **17** as a colorless solid (Method **A**: 52.9 mg, 69%; Method **B**: 8.3 mg, 11%); ^1^H NMR (400 MHz, DMSO-*d*_6_): δ 8.34 (d, *J* = 8 Hz, 1H), 8.18 (d, *J* = 8 Hz, 1H), 7.94 (s, 2H), 7.79 (s, 1H), 7.58 (s, 2H), 7.43 (d, *J* = 8.8 Hz, 1H), 6.78 (s, 1H), 6.52 (d, *J* = 8.8 Hz, 1H); ^13^C­{^1^H} NMR (100 MHz, DMSO-*d*_6_): δ 162.3, 160.8, 151.1, 150.4, 137.5, 135.2, 132.2, 125.8, 125.5, 125.0, 116.0, 114.9, 113.9, 110.9; HRMS (FAB-Magnetic Sector): calcd for C_14_H_11_Cl_2_N_4_ [M + H]^+^, 305.0361; found, 306.0355.

## Supplementary Material







## Data Availability

The data underlying this study are available in the published article and its online Supporting Information.

## Notes

Crystal data of **4** and **6ka**, and NMR spectra of the prepared products (PDF)
Data (CIF)
Data (CIF)
