# Supplementary material for: Gallium/Indium-Promoted [4 + 2] Annulation of 2‑Aminobenzonitriles with Alkynes and Dimerization of 2‑Aminobenzonitriles: One-Pot Access to 4‑Aminoquinolines and 4‑Amino-2-Arylquinazolines
Source: ACS Omega. 2026 May 28;11(22):32813–24. doi: 10.1021/acsomega.6c01857 (PMC13261441; doi:10.1021/acsomega.6c01857)

Supporting Information

Gallium/Indium-Promoted [4+2] Annulation of 2-Aminobenzonitriles with Alkynes and Dimerization of 2-Aminobenzonitriles: One-Pot Access to 4-Aminoquinolines and 4-Amino-2-Arylquinazolines

Norio Sakai,* Mio Ishii, Shuji Yamauchi, Yohei Ogiwara, and Kento Ishida

Department of Pure and Applied Chemistry, Faculty of Science and Technology,
Tokyo University of Science (RIKADAI), Noda, Chiba 278-8510, Japan

E-mail: sakachem@rs.tus.ac.jp

Table of Contents

1. Crystal data for compound **6ka** and compound **4** S2-S3
2. NMR charts of the prepared compounds S4-S36

Figure S1. Crystal data for compound **6ka**

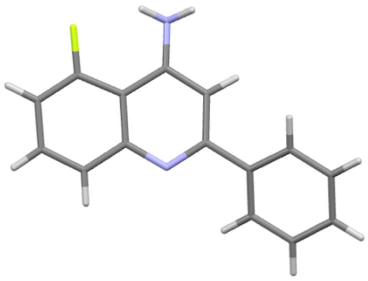

CCDC 2475859

Empirical formula	$C_{15}H_{11}FN_2$
Formula weight:	238.26
Temperature/K:	101(1)
Crystal system:	monoclinic
Space group:	P2 1/c
a/Å:	10.3210(2)
b/Å:	17.4949(3)
c/Å:	12.5336(2)
α /°:	90
β /°:	94.4840(10)
γ /°:	90
Volume/Å^3:	2256.20(7)
Z:	10
ρ_{calc} g/cm^3:	1.754
μ /mm^{-1}:	0.120
F (000):	1240.0
Crystal size/mm^3:	5 × 5 × 20
Radiation:	Mo Kα (λ = 0.71073)
2θ range for data collection/°:	4.592 to 60.648
Index ranges:	$-14 \leq h \leq 14$, $-24 \leq k \leq 23$, $-16 \leq l \leq 17$
Reflections collected:	52964
Independent reflections:	5989 [R_{int} = 0.0692, R_{sigma} = 0.0364]
Data/restraints/parameters:	5989/0/328
Goodness-of-fit on F^2:	1.062
Final R indexes [I>=2σ (I)]:	R_1 = 0.0494, wR_2 = 0.1297
Final R indexes [all data]:	R_1 = 0.0587, wR_2 = 0.1366

Figure S2. Crystal data for compound **4**

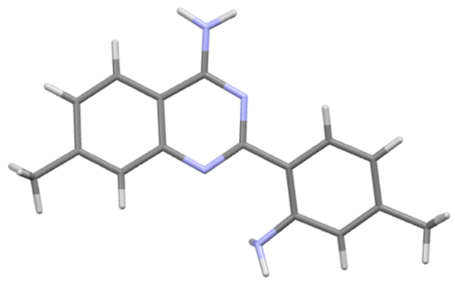

CCDC 2405427

Empirical formula:	$C_{16}H_{16}N_4$
Formula weight:	264.33
Temperature/K:	93.9(2)
Crystal system:	monoclinic
Space group:	I 2/a
a/Å:	22.7871(7)
b/Å:	10.2022(2)
c/Å:	27.1063(8)
α /°:	90
β /°:	119.000(4)
γ /°:	90
Volume/Å^3:	5511.5(3)
Z:	8
ρ_{calcg}/cm^3:	1.274
μ /mm^{-1}:	0.079
F (000):	2240.0
Crystal size/mm^3:	5 × 3 × 20
Radiation:	Mo Kα (λ = 0.71073)
2θ range for data collection/°:	5.012 to 60.586
Index ranges:	$-30 \le h \le 31$, $-14 \le k \le 13$, $-36 \le l \le 37$
Reflections collected:	61482
Independent reflections:	7427 [R_{int} = 0.0418, R_{sigma} = 0.0232]
Data/restraints/parameters:	7427/0/368
Goodness-of-fit on F^2:	1.067
Final R indexes [I>=2σ (I)]:	R_1 = 0.0469, wR_2 = 0.1326
Final R indexes [all data]:	R_1 = 0.0558, wR_2 = 0.1383

Figure S3. ^{1}H NMR of **3a** (400 MHz, $CDCl_3$)

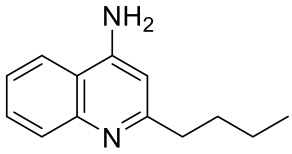

3a

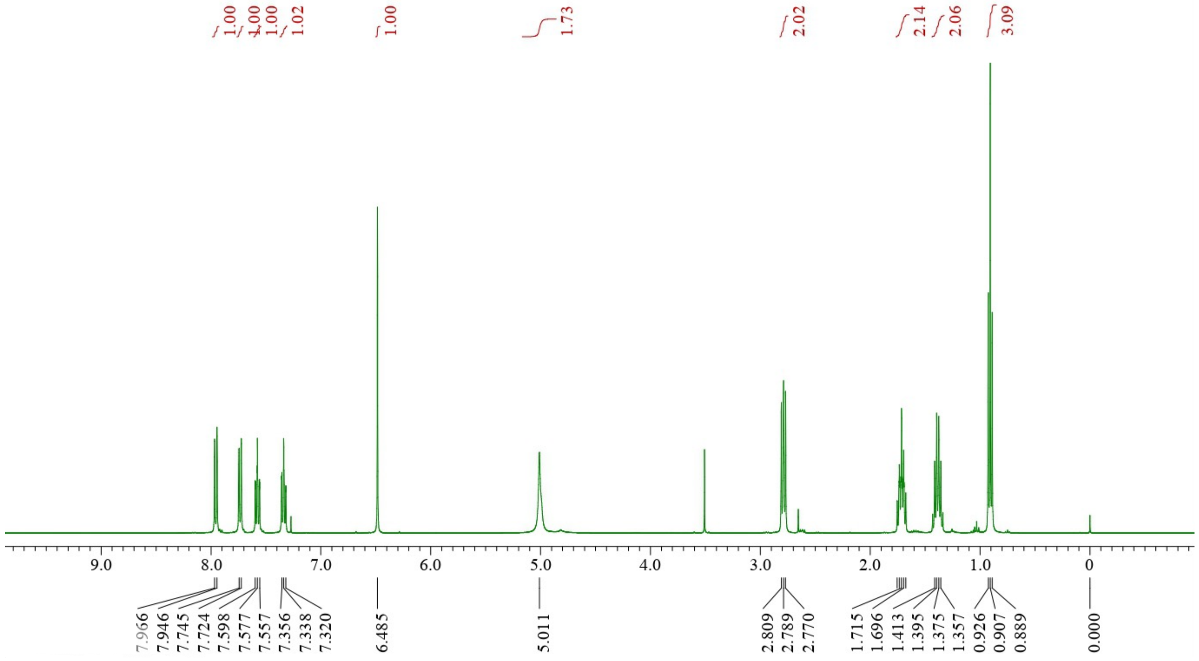

Figure S4. $^{13}C\{^{1}H\}$ NMR of **3a** (100 MHz, $CDCl_3$)

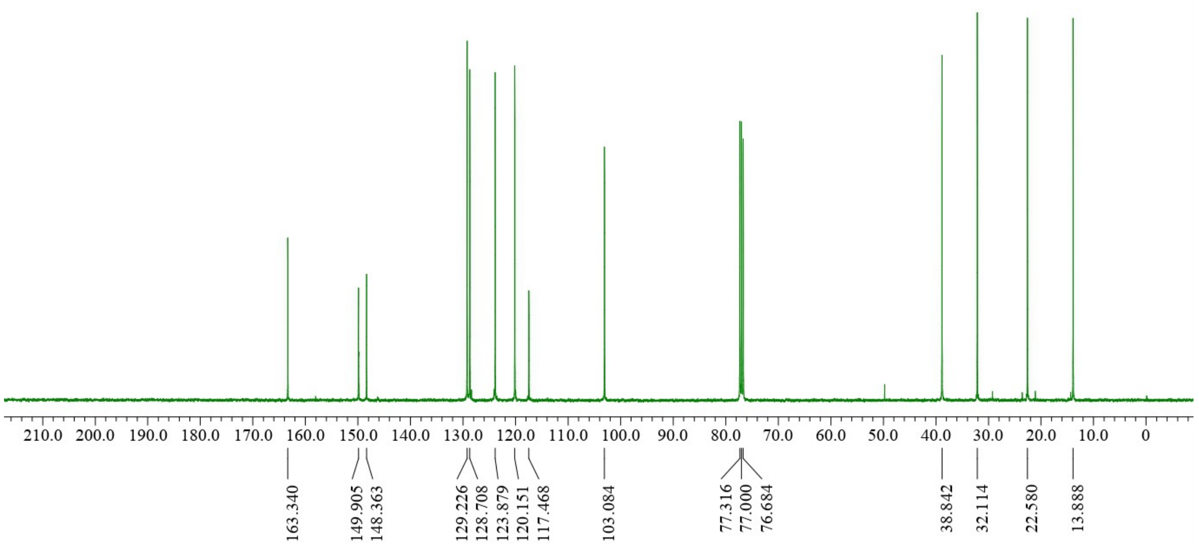

Figure S5. ^{1}H NMR of **3b** (400 MHz, $CDCl_3$)

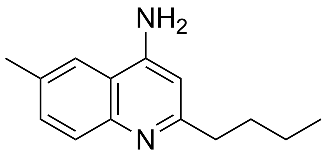

3b

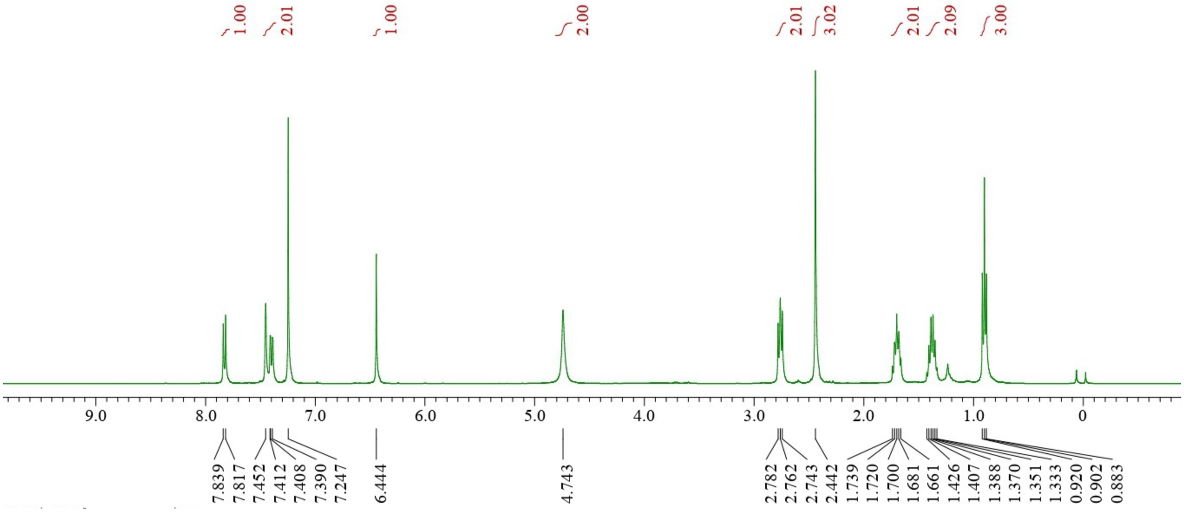

Figure S6. $^{13}C\{^{1}H\}$ NMR of **3b** (100 MHz, $CDCl_3$)

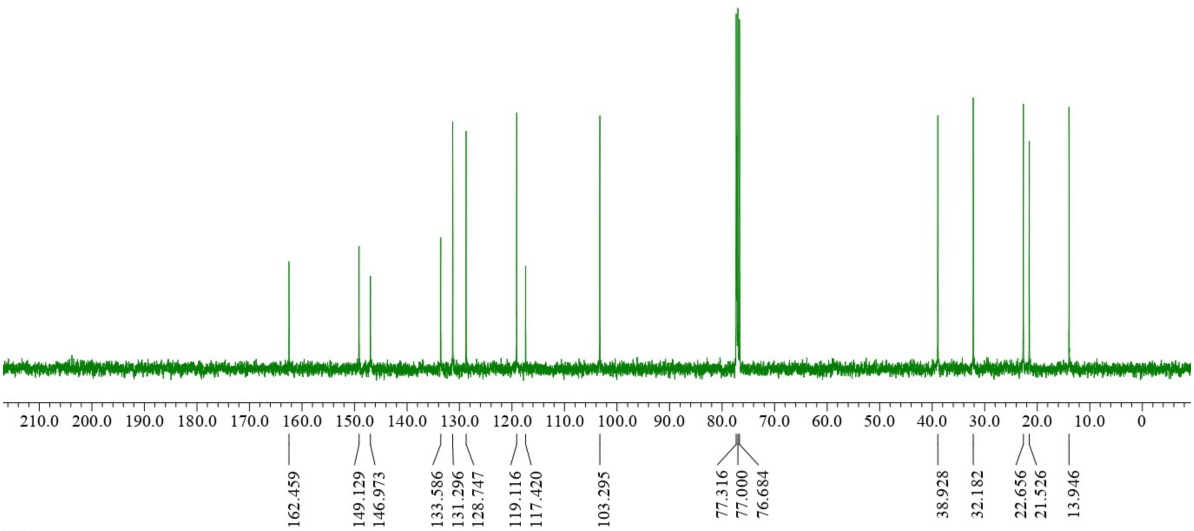

Figure S7. ^{1}H NMR of **3c** (400 MHz, $CDCl_3$)

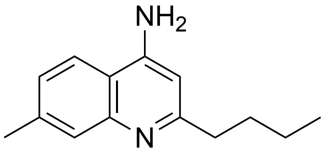

3c

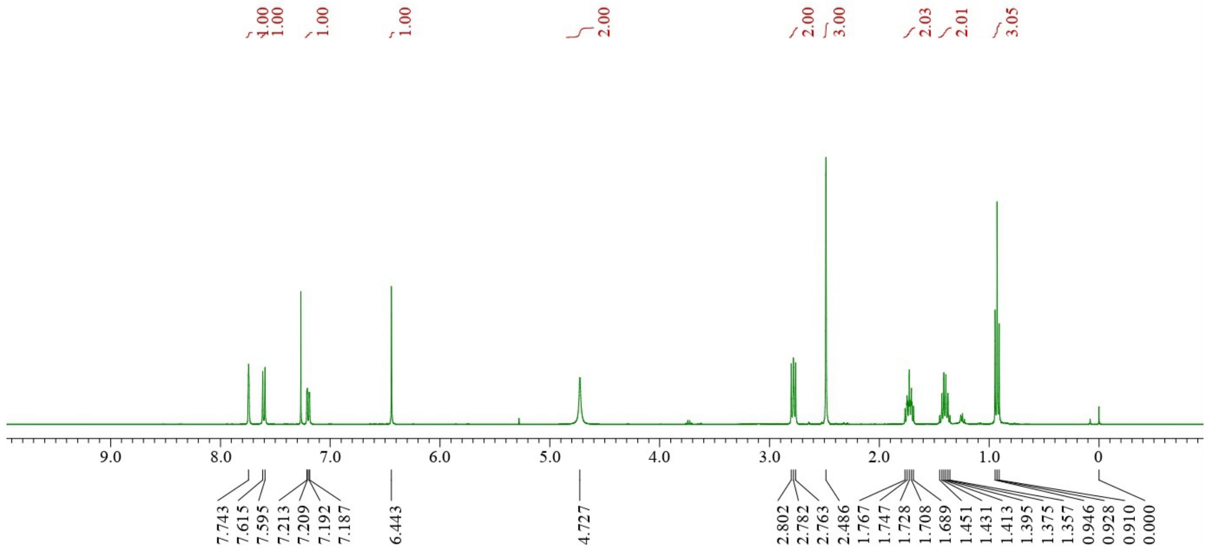

Figure S8. $^{13}C\{^{1}H\}$ NMR of **3c** (100 MHz, $CDCl_3$)

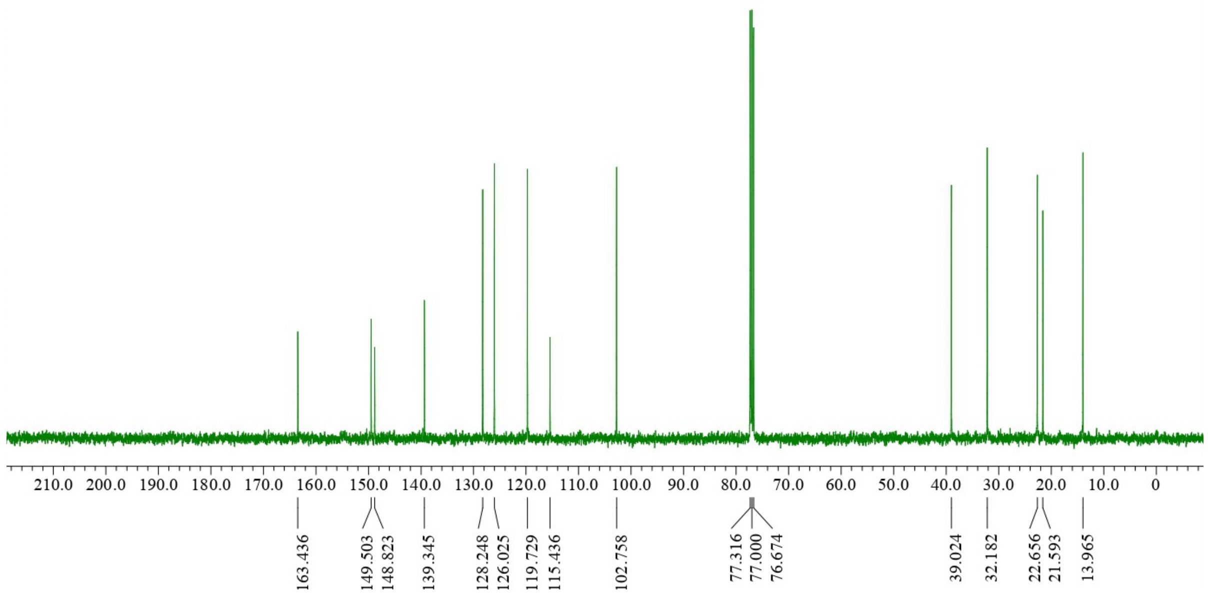

Figure S9. ^{1}H NMR of **3d + 3d'** (400 MHz, $CDCl_3$)

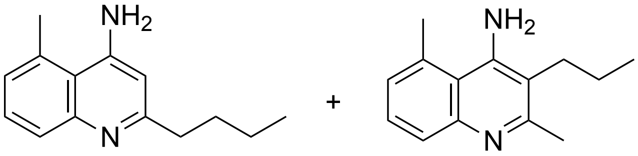

3d ▲ **3d'** ▲

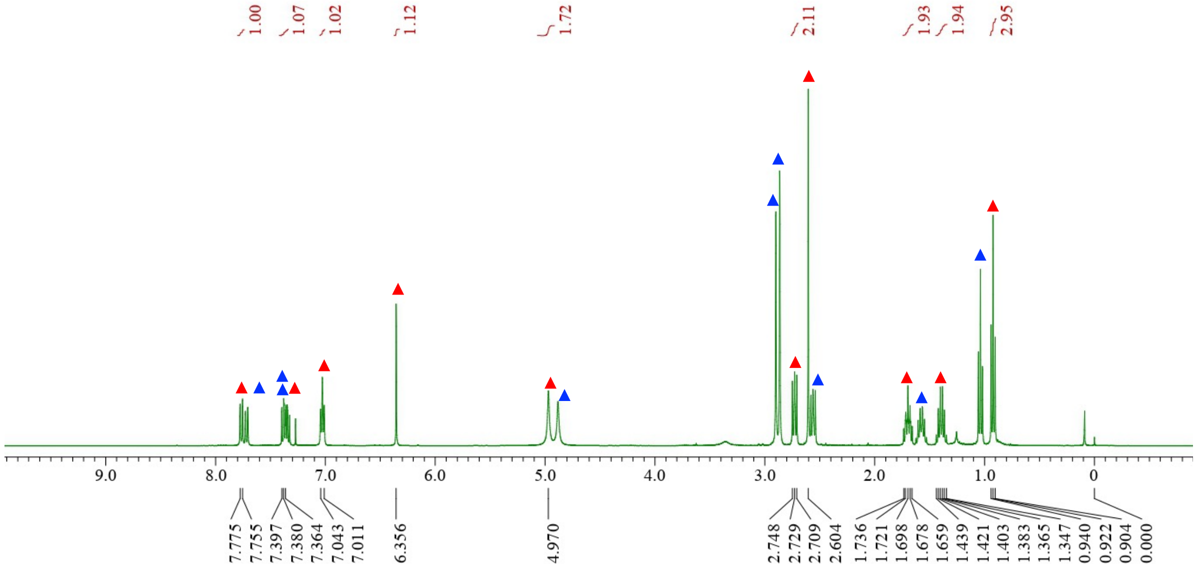

Figure S10. $^{13}C\{^1H\}$ NMR of **3d + 3d'** (100 MHz, $CDCl_3$)

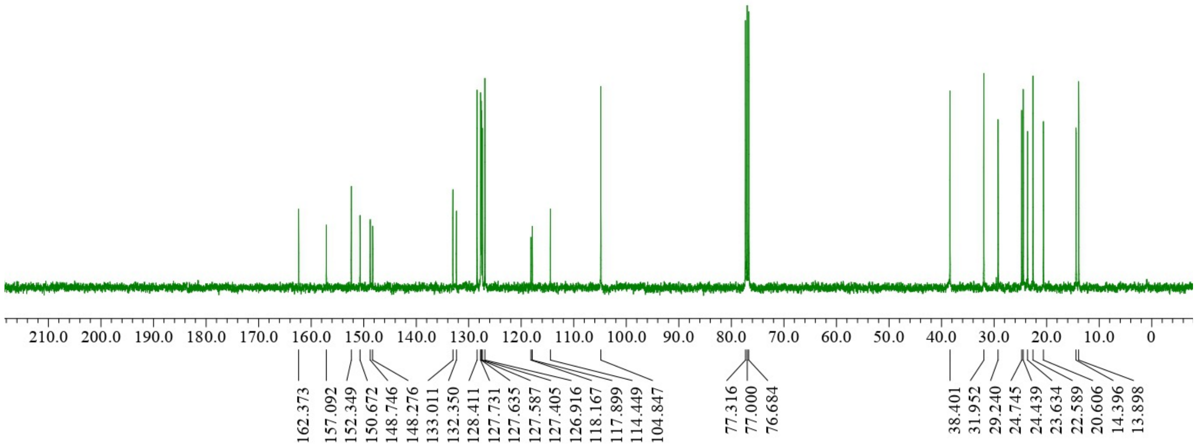

Figure S11. ^{1}H NMR of **3e** (500 MHz, $CDCl_3$)

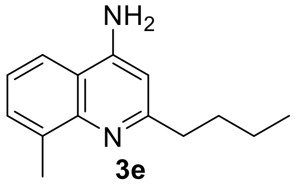

3e

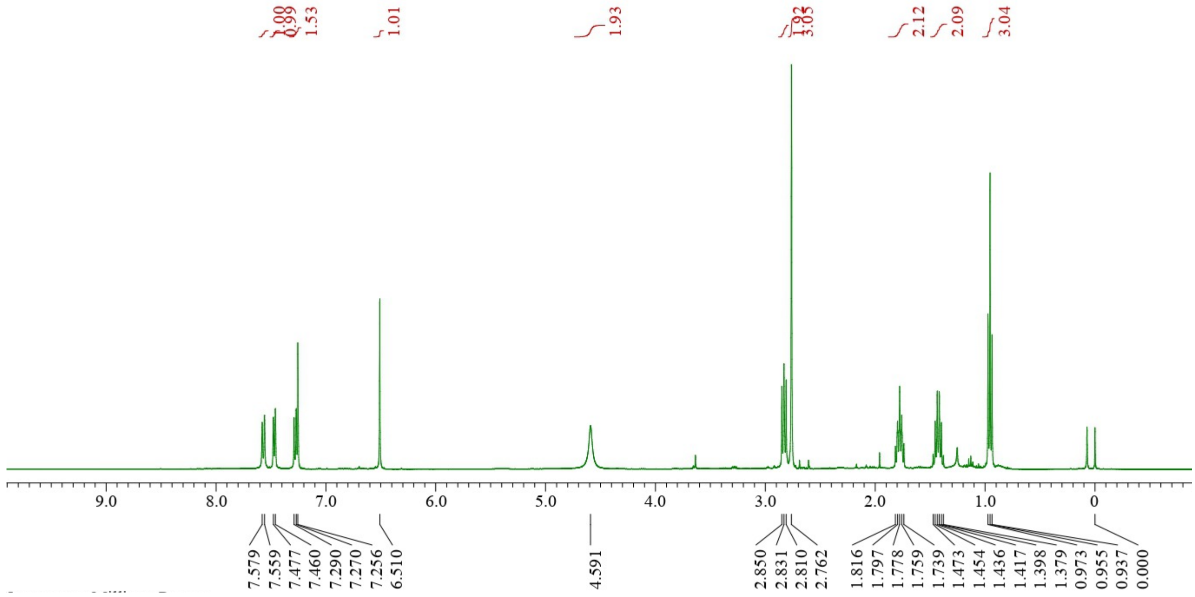

Figure S12. ^{13}C{^{1}H} NMR of **3e** (125 MHz, $CDCl_3$)

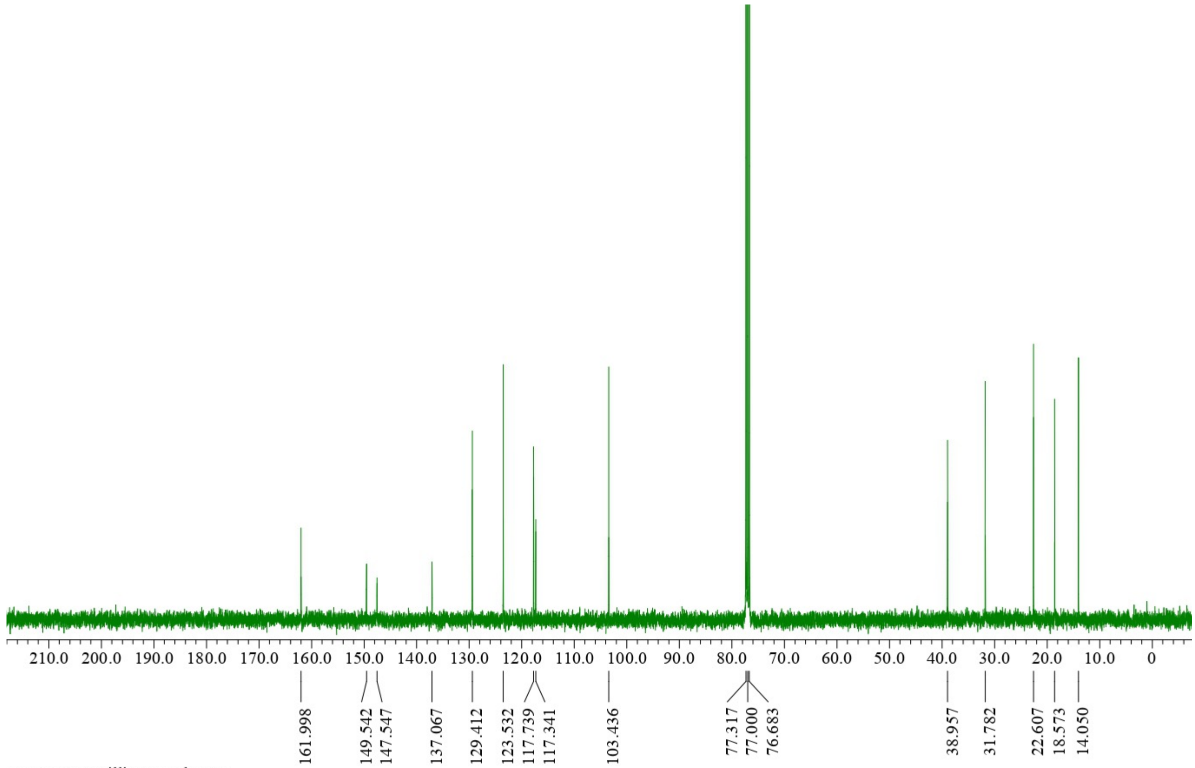

Figure S13. ^{1}H NMR of **3f + 3f'** (400 MHz, $CDCl_3$)

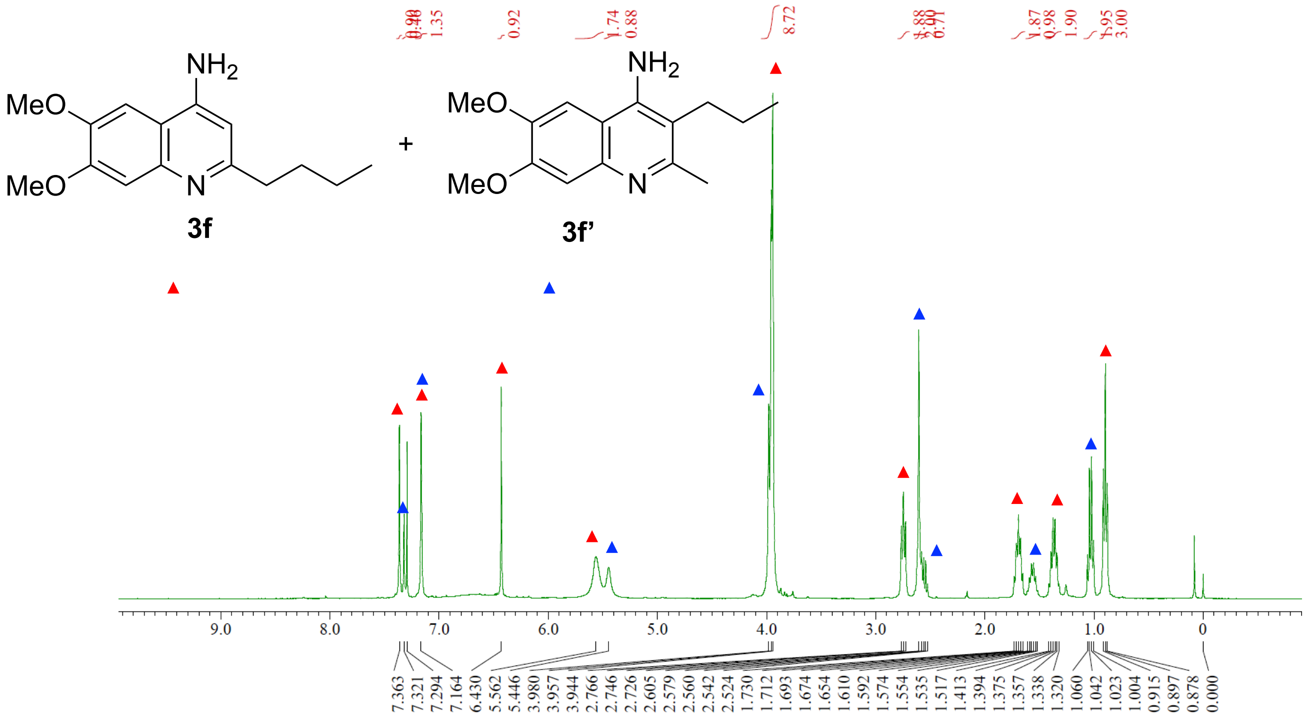

Figure S14. $^{13}C\{^1H\}$ NMR of **3f +3f'** (100 MHz, $CDCl_3$)

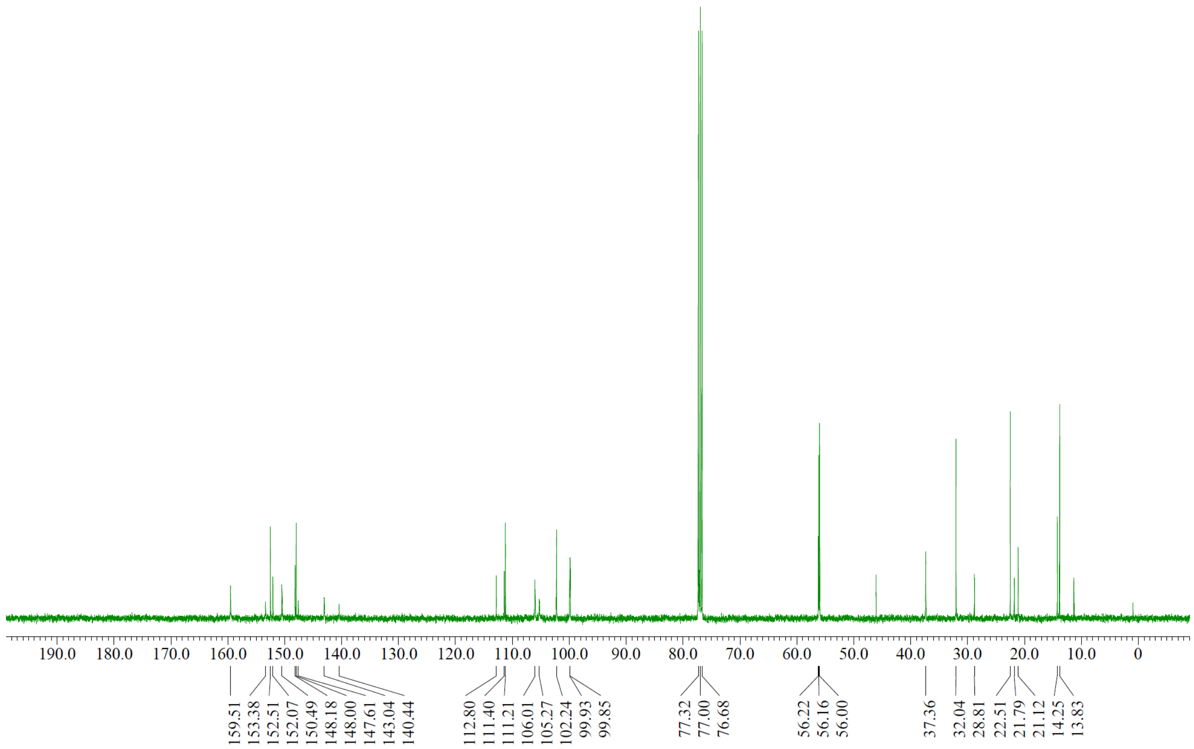

Figure S15. ^{1}H NMR of **3g** (500 MHz, $CDCl_3$)

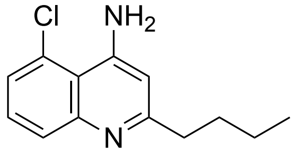

3g

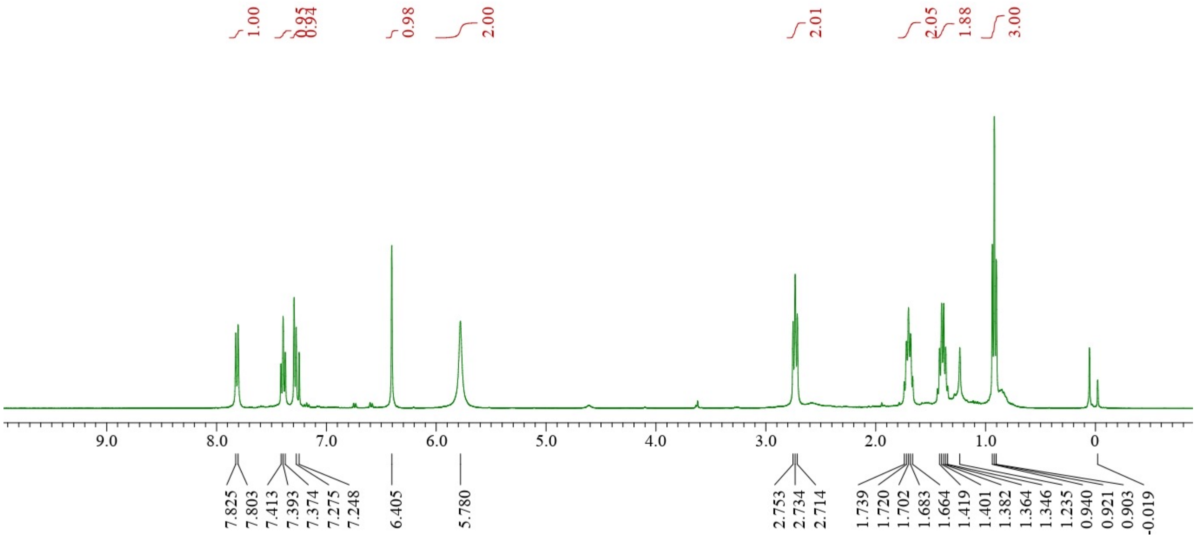

Figure S16. $^{13}C\{^{1}H\}$ NMR of **3g** (125 MHz, $CDCl_3$)

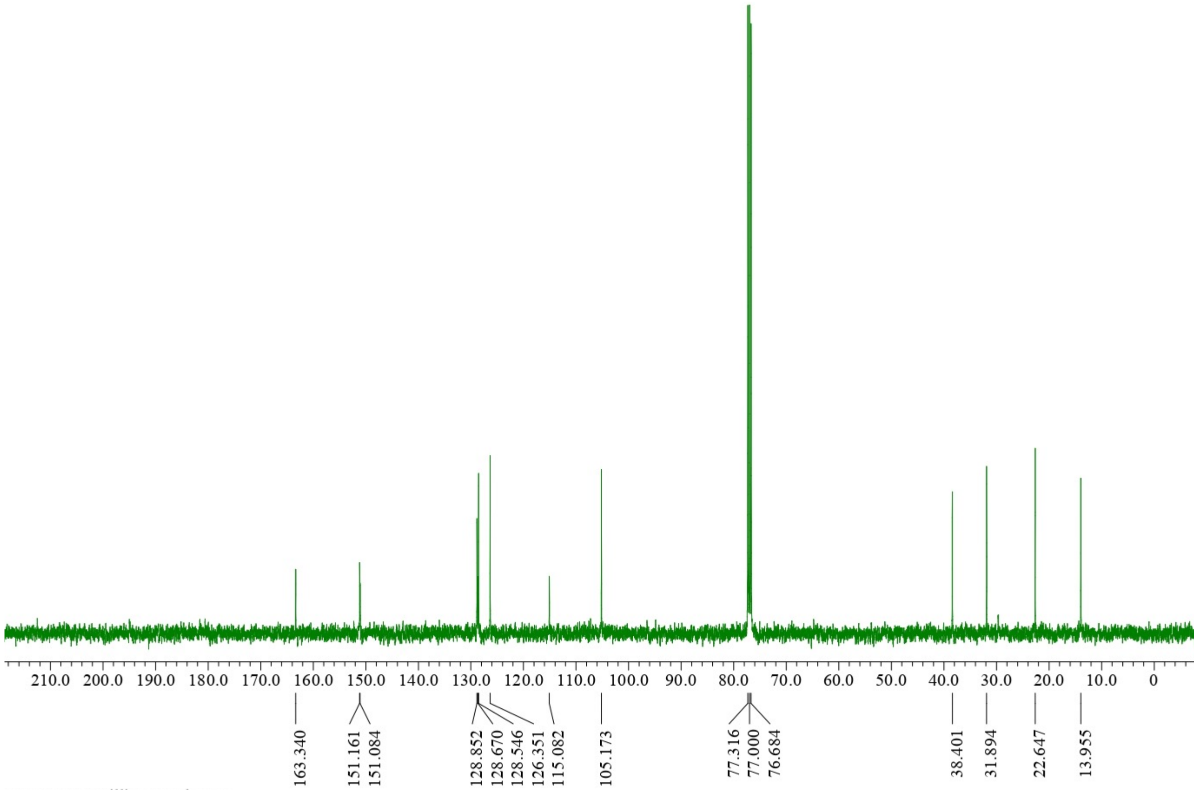

Figure S17. ^{1}H NMR of **3h** (500 MHz, DMSO-d_6)

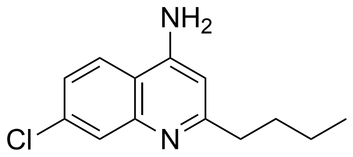

3h

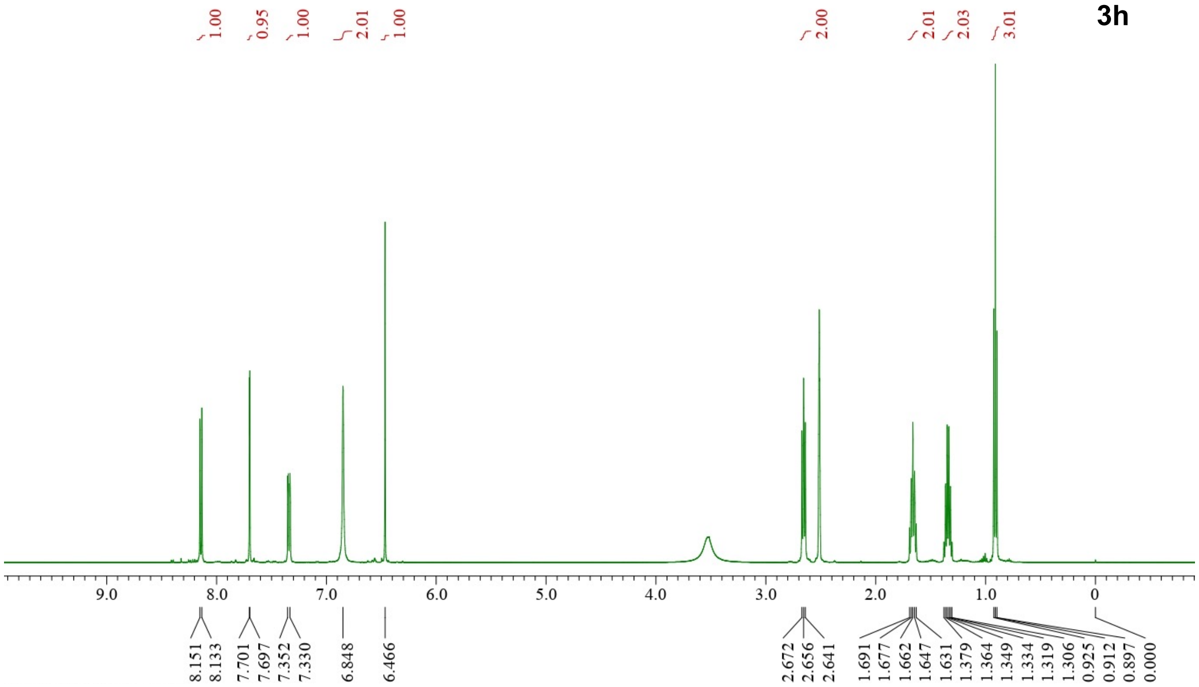

Figure S18. ^{13}C{^{1}H} NMR of **3h** (125 MHz, DMSO-d_6)

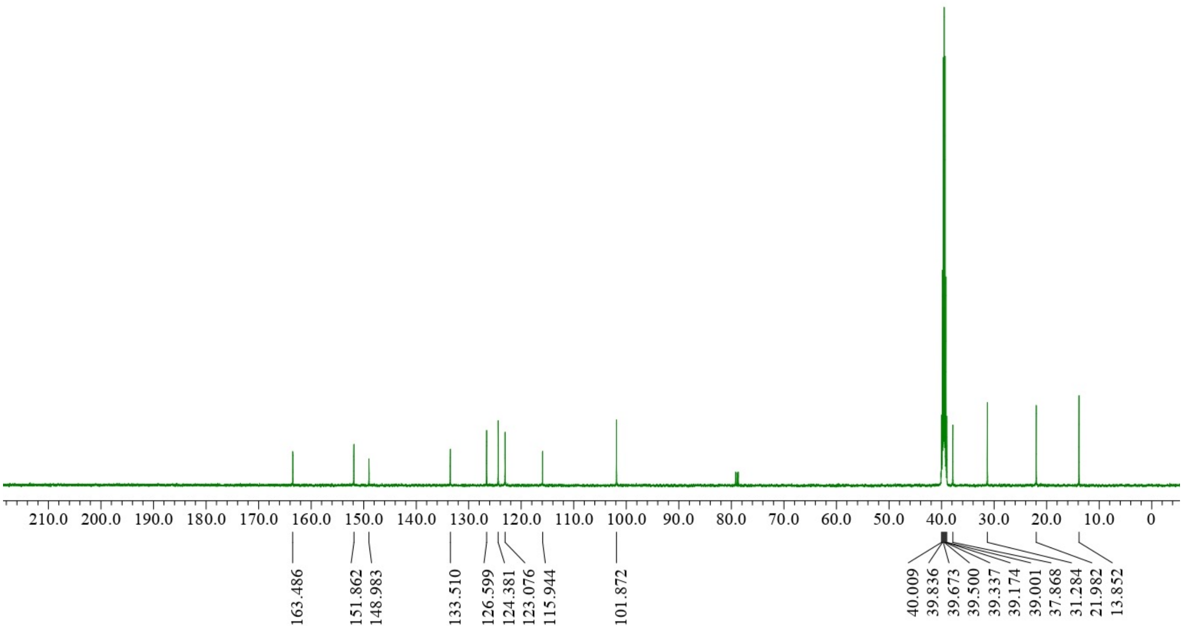

Figure S19. ^{1}H NMR of **3i** (400 MHz, $CDCl_3$)

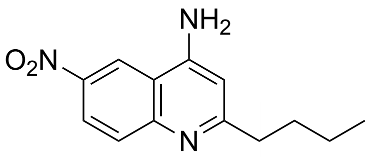

3i

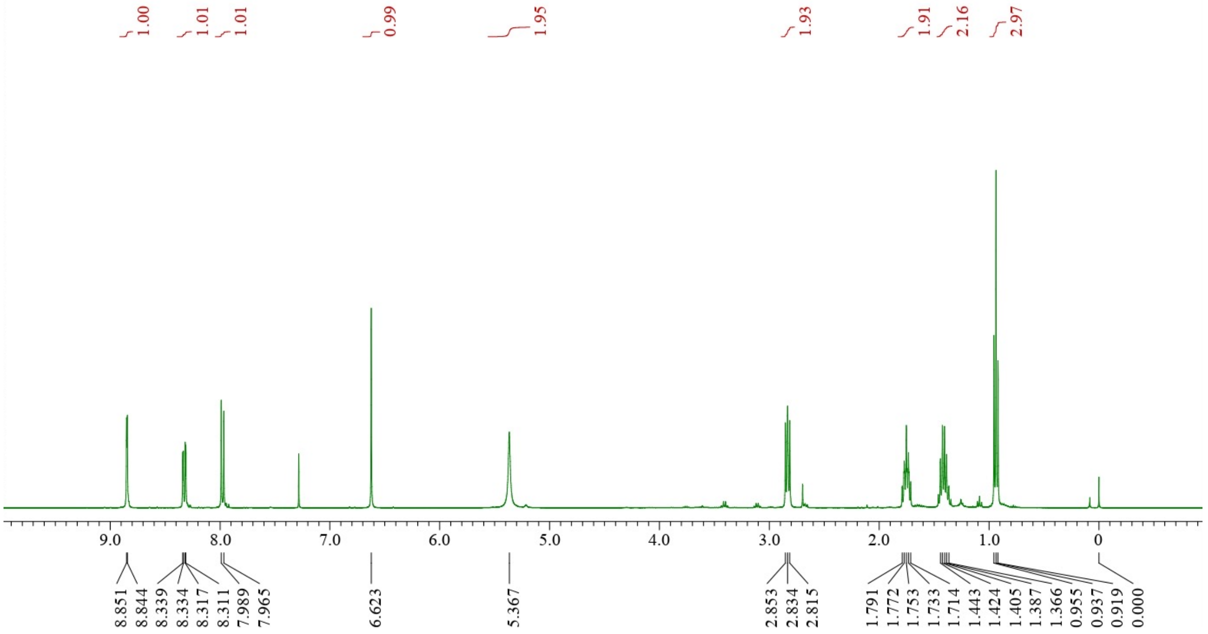

Figure S20. $^{13}C\{^{1}H\}$ NMR of **3i** (100 MHz, $CDCl_3$)

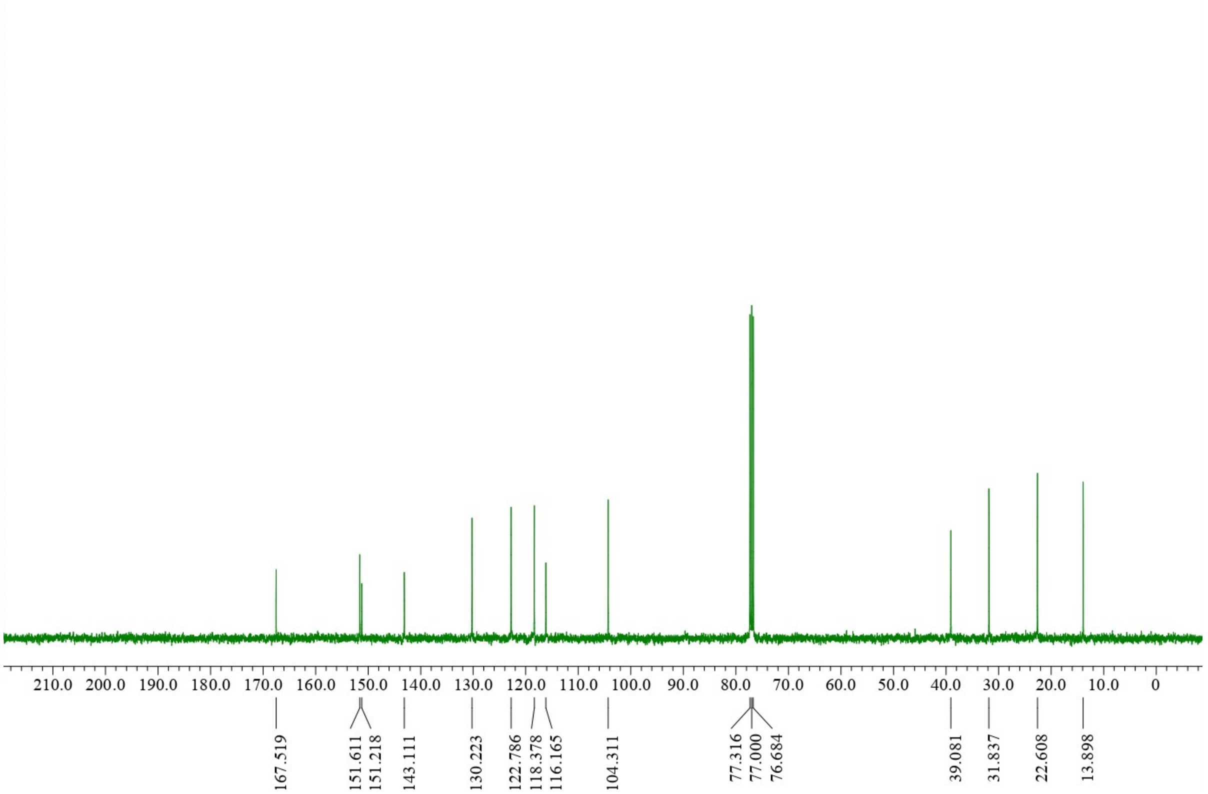

Figure S21. ^{1}H NMR of **3j** (400 MHz, $CDCl_3$)

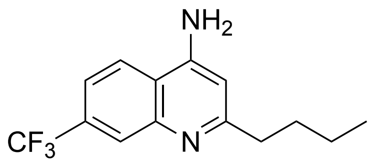

3j

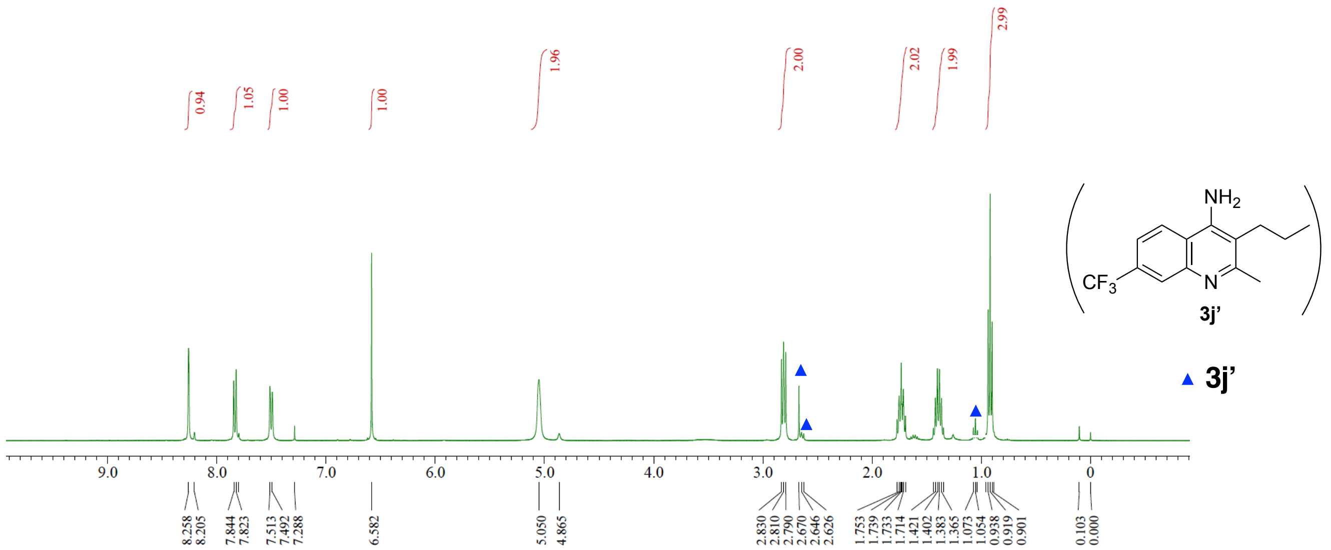

Figure S22. $^{13}C\{^{1}H\}$ NMR of **3j** (100 MHz, $CDCl_3$)

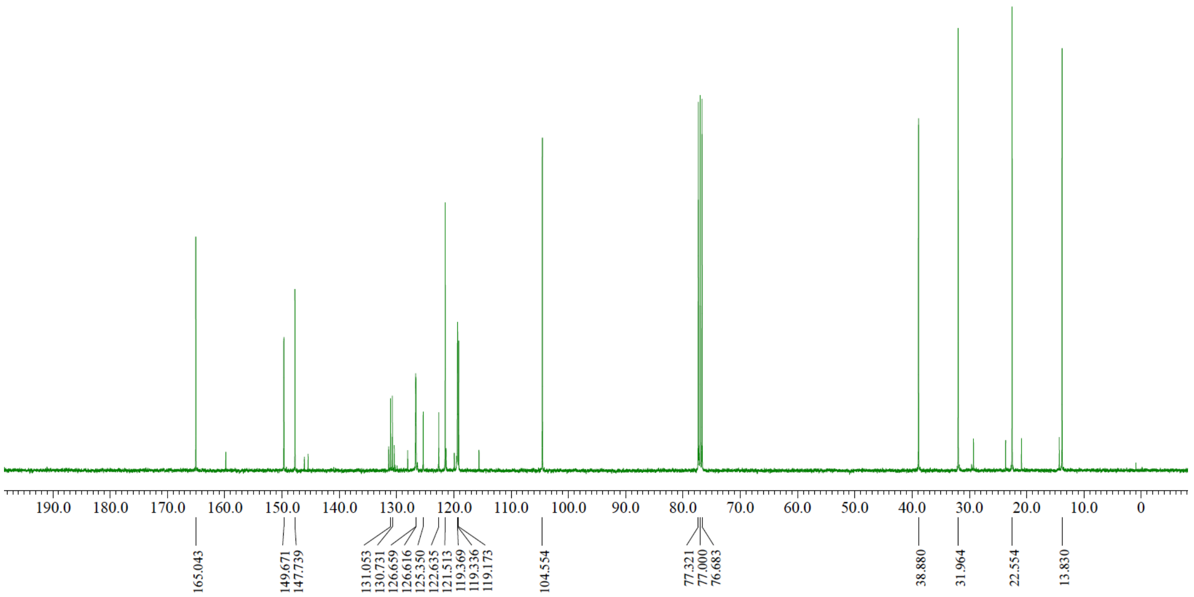

Figure S23. ^{19}F NMR of **3j** (376 MHz, $CDCl_3$)

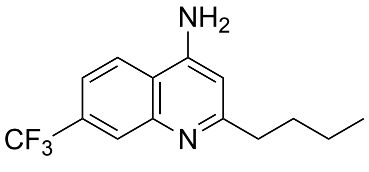

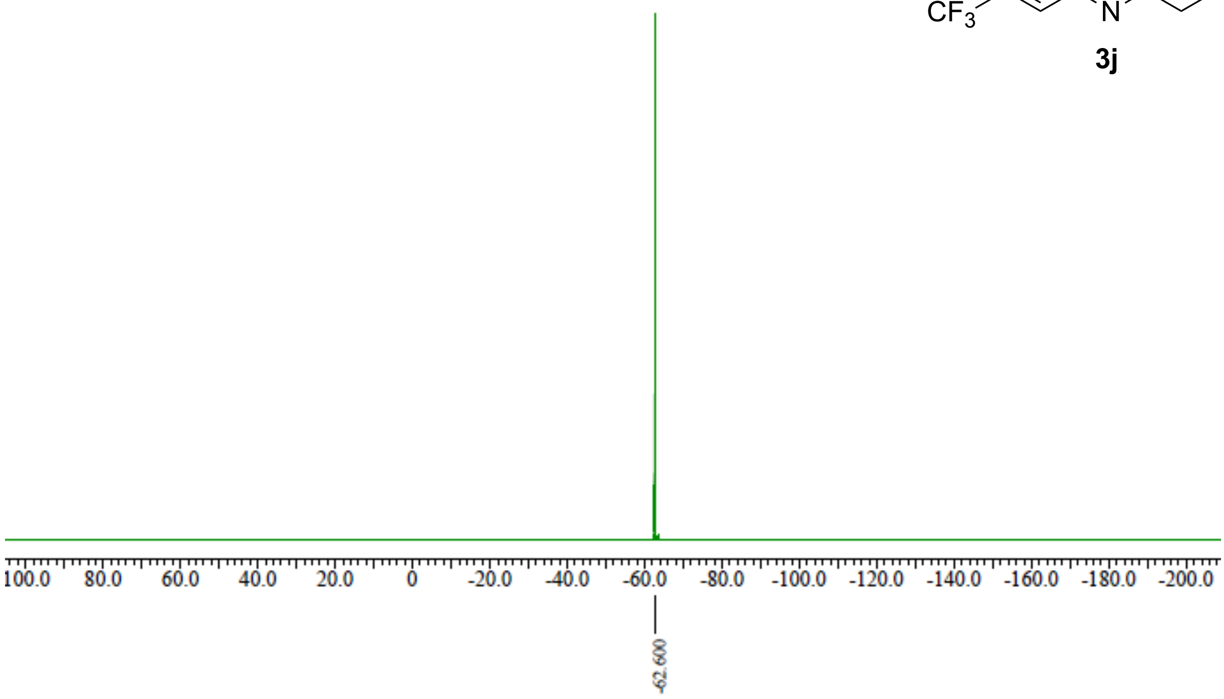

Figure S24. ^{1}H NMR of **4** (400 MHz, $CDCl_3$)

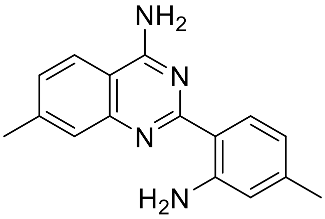

4

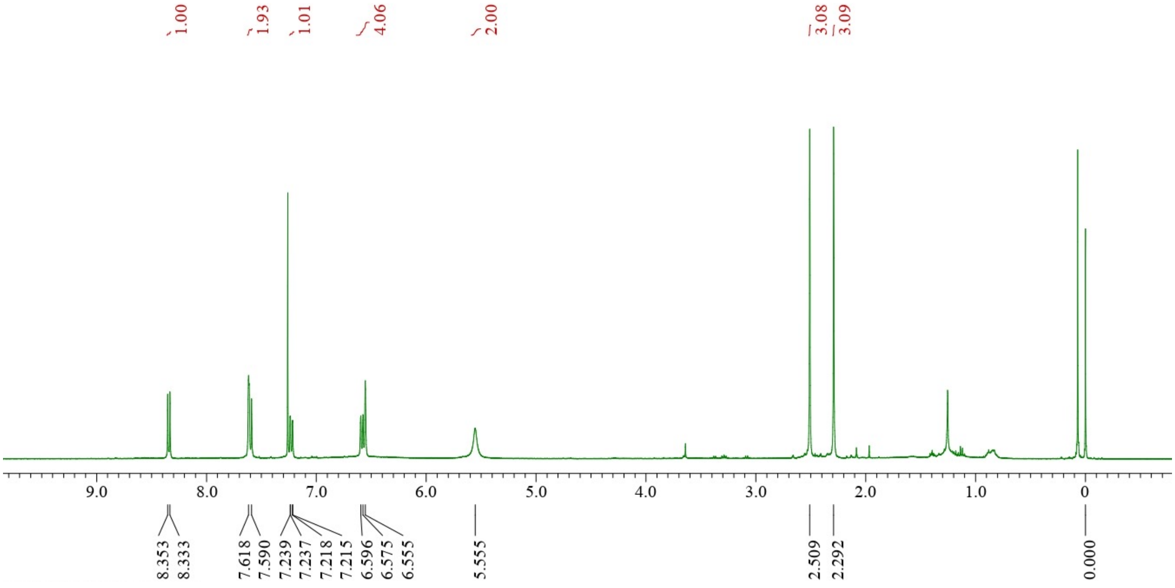

Figure S25. ^{13}C{^{1}H} NMR of **4** (100 MHz, $CDCl_3$)

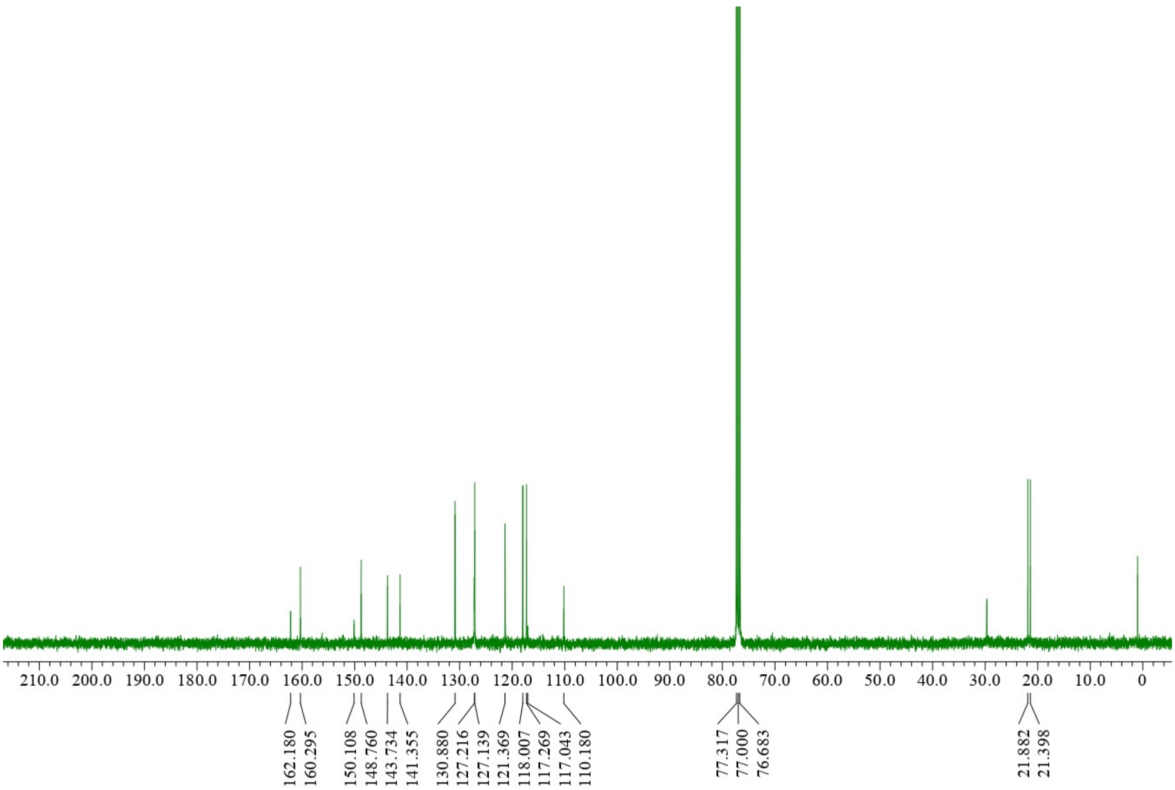

Figure S26. ^{1}H NMR of **6aa** (500 MHz, DMSO-d_6)

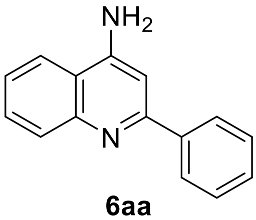

6aa

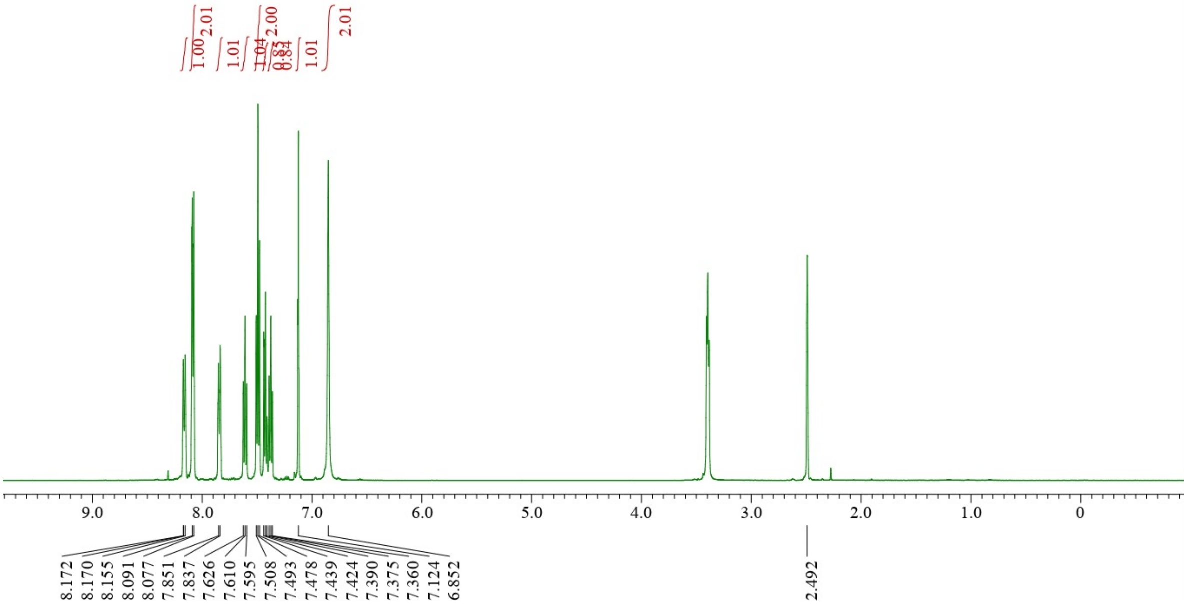

Figure S27. ^{13}C{^{1}H} NMR of **6aa**(125 MHz, DMSO-d_6)

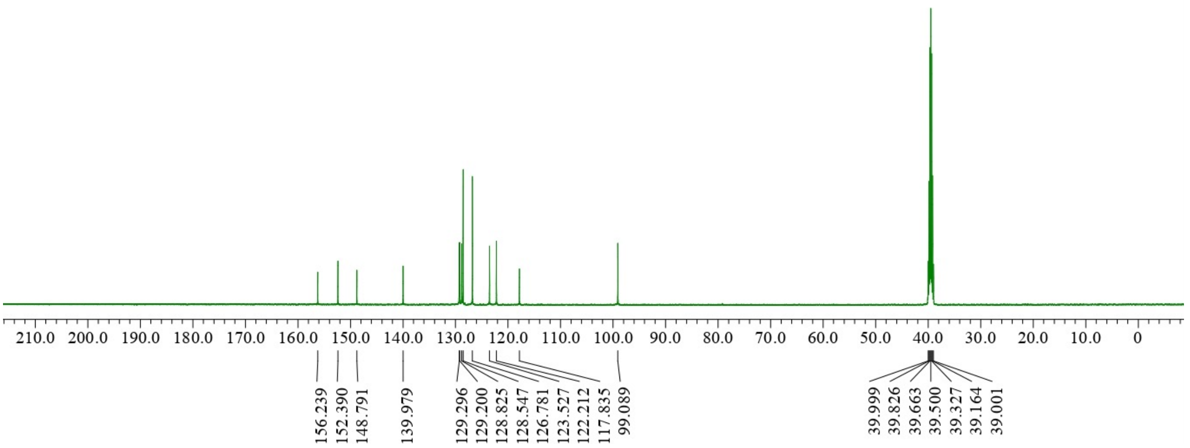

Figure S28. ^{1}H NMR of **6ab** (500 MHz, $CDCl_3$)

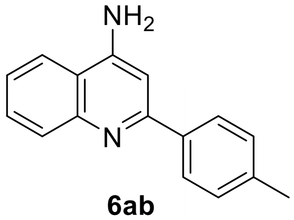

6ab

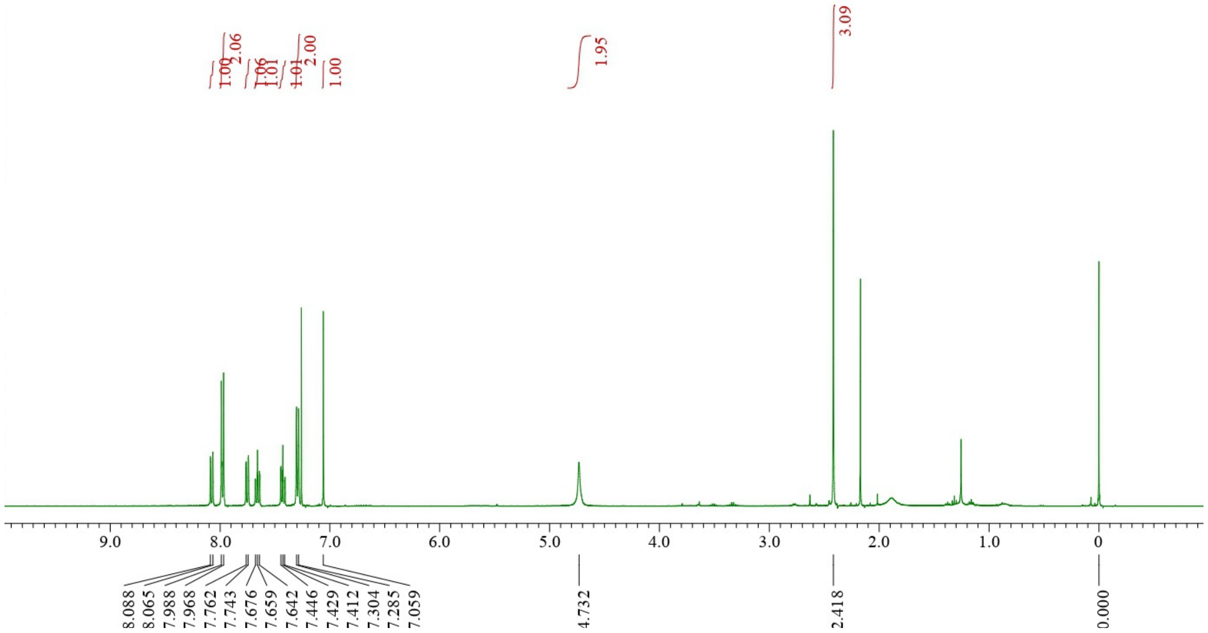

Figure S29. $^{13}C\{^{1}H\}$ NMR of **6ab** (125 MHz, $CDCl_3$)

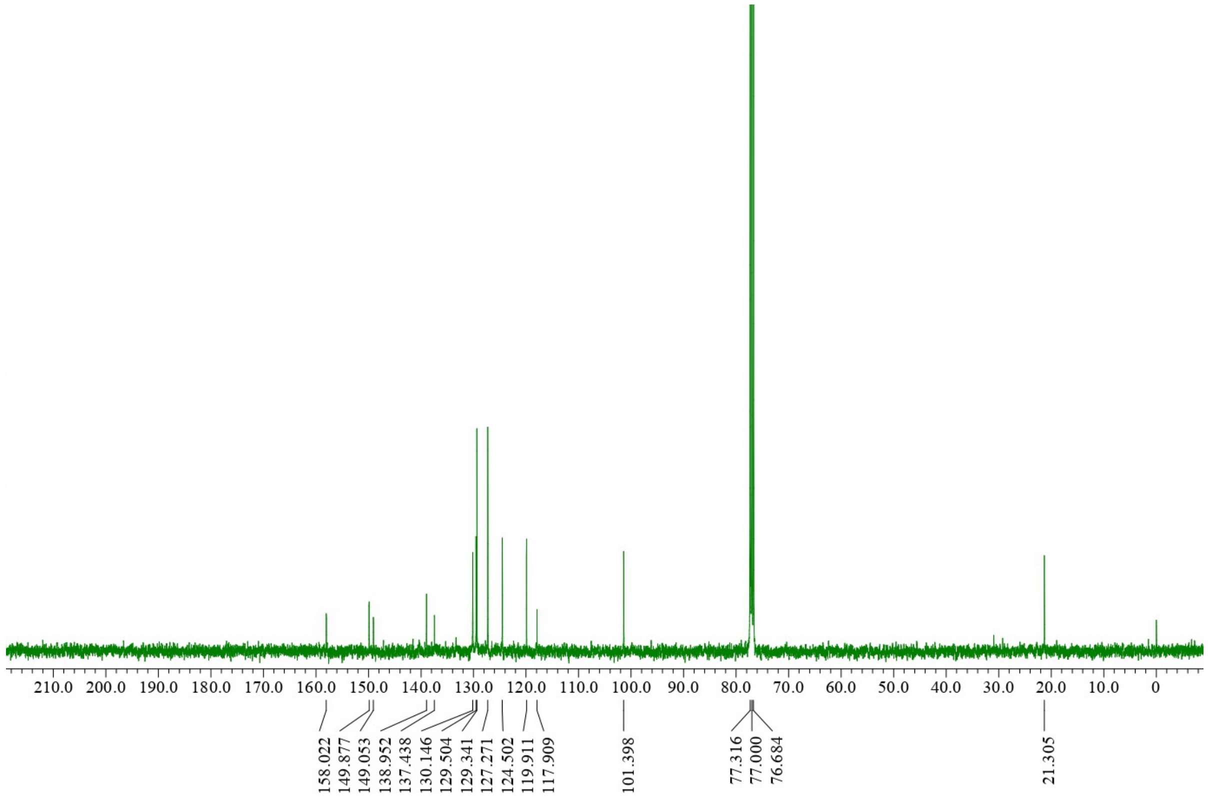

Figure S30. ^{1}H NMR of **6ac** (500 MHz, DMSO-d_6)

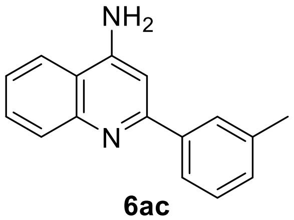

6ac

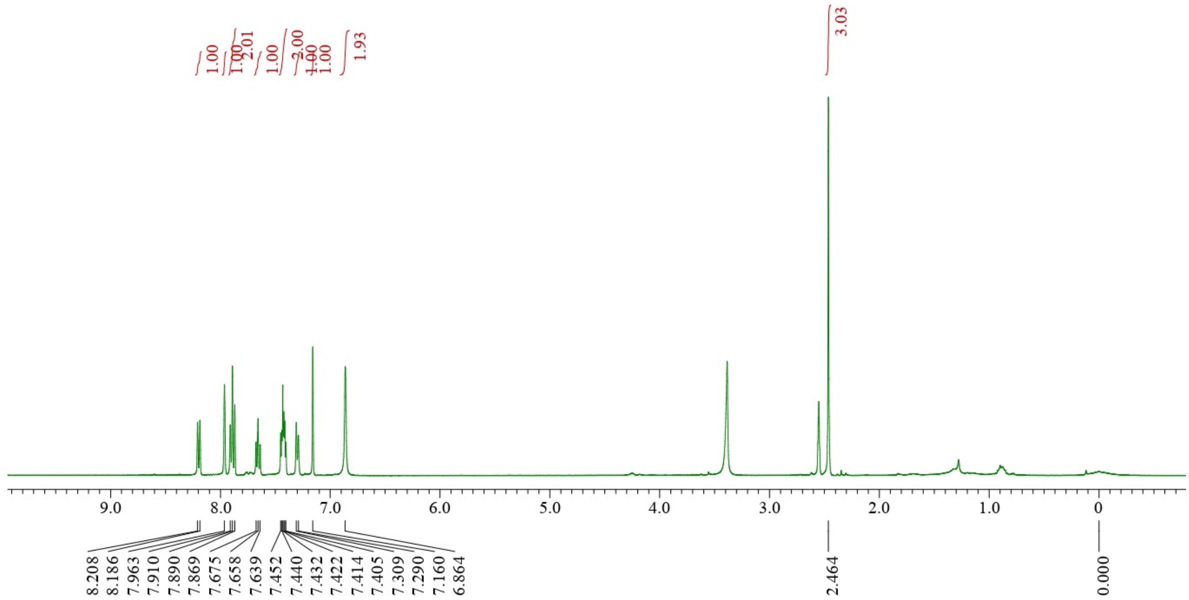

Figure S31. $^{13}C\{^{1}H\}$ NMR of **6ac** (125 MHz, DMSO-d_6)

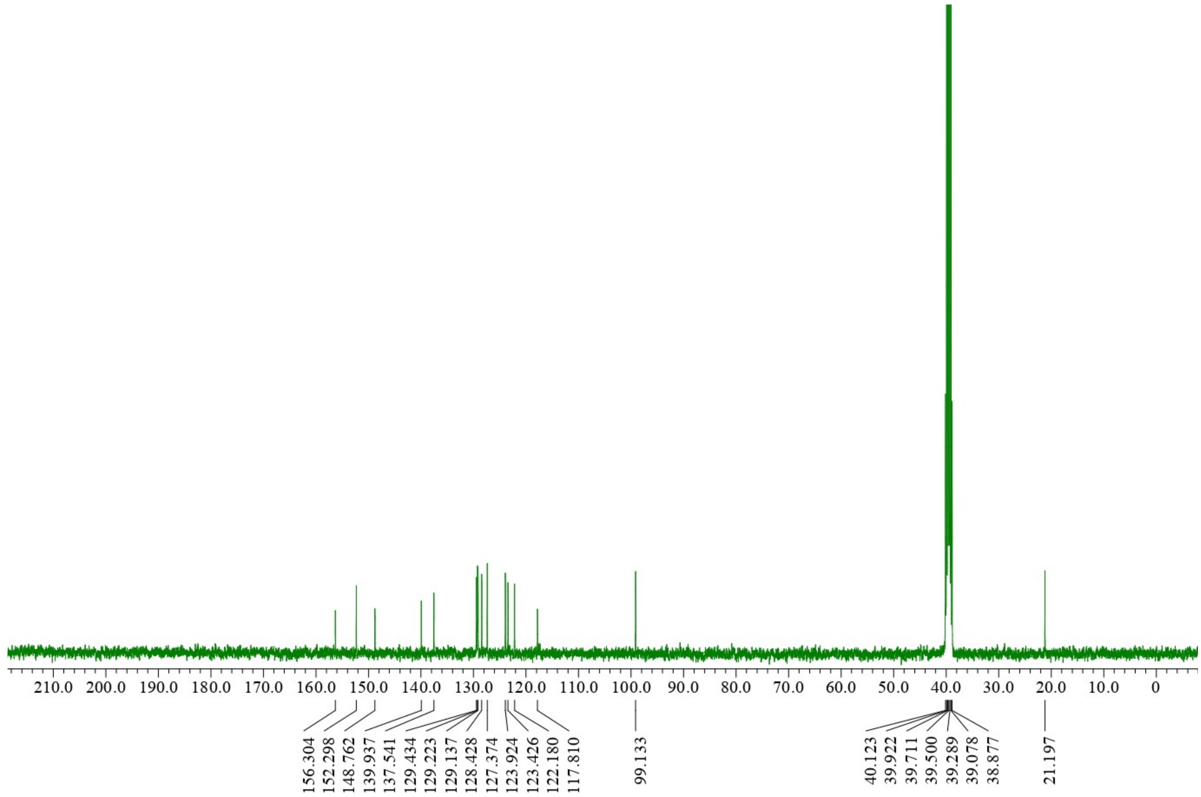

Figure S32. ^{1}H NMR of **6ad** (400 MHz, $CDCl_3$)

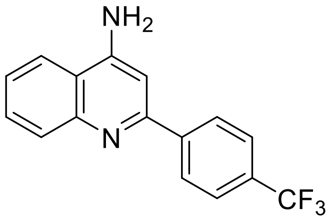

6ad

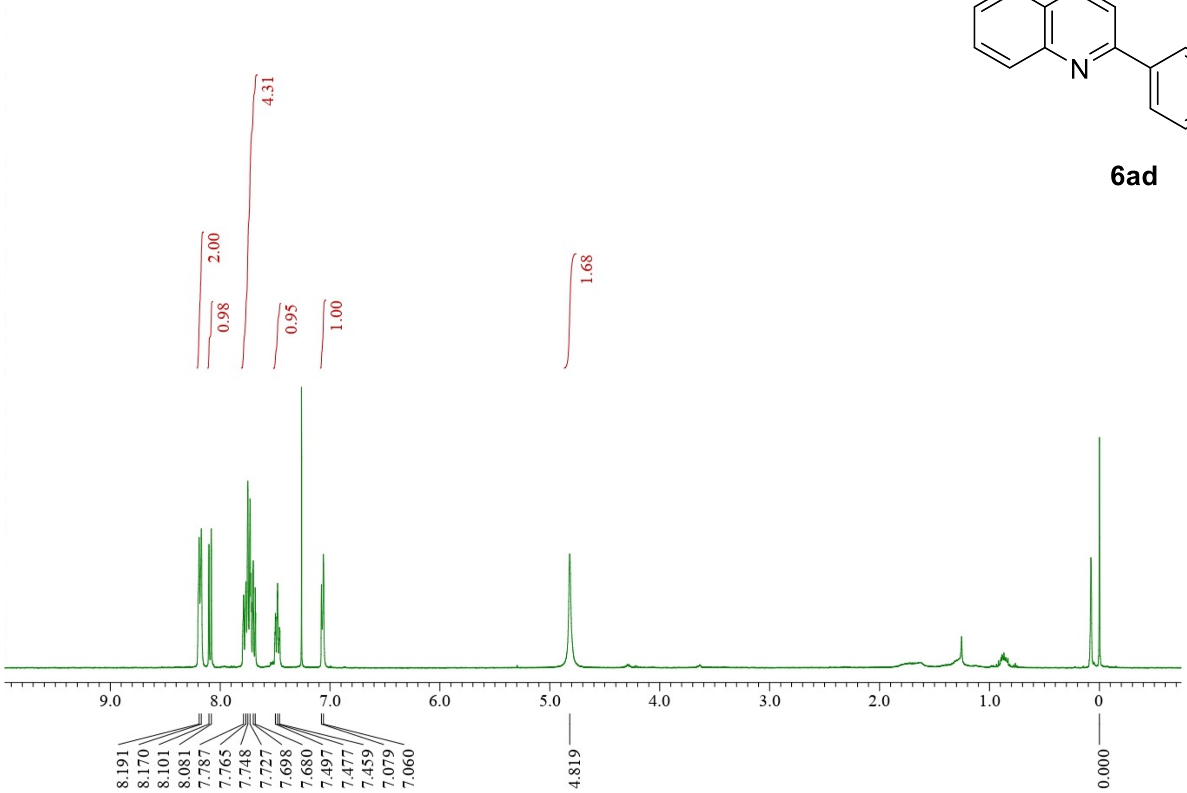

Figure S33. $^{13}C\{^{1}H\}$ NMR of **6ad** (100 MHz, $CDCl_3$)

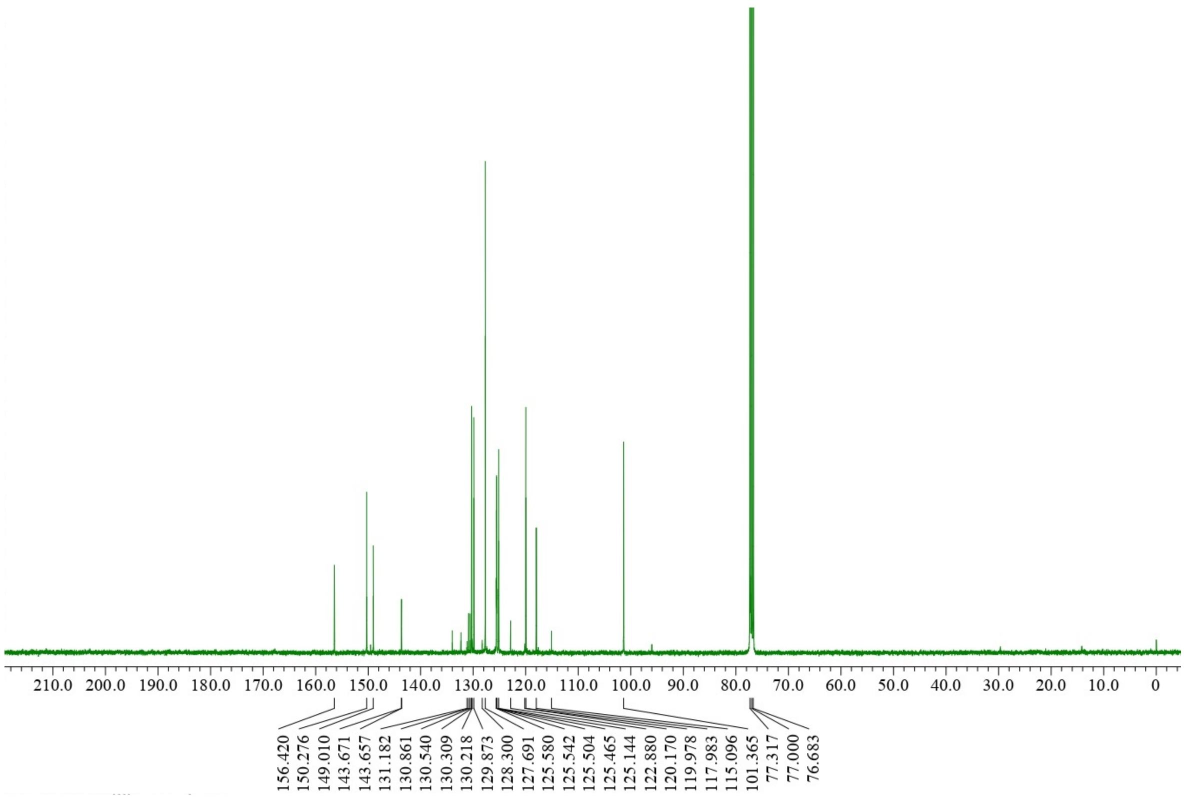

Figure S34. ^{19}F NMR of **6ad** (376 MHz, $CDCl_3$)

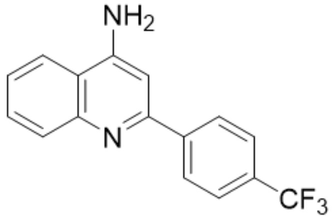

6ad

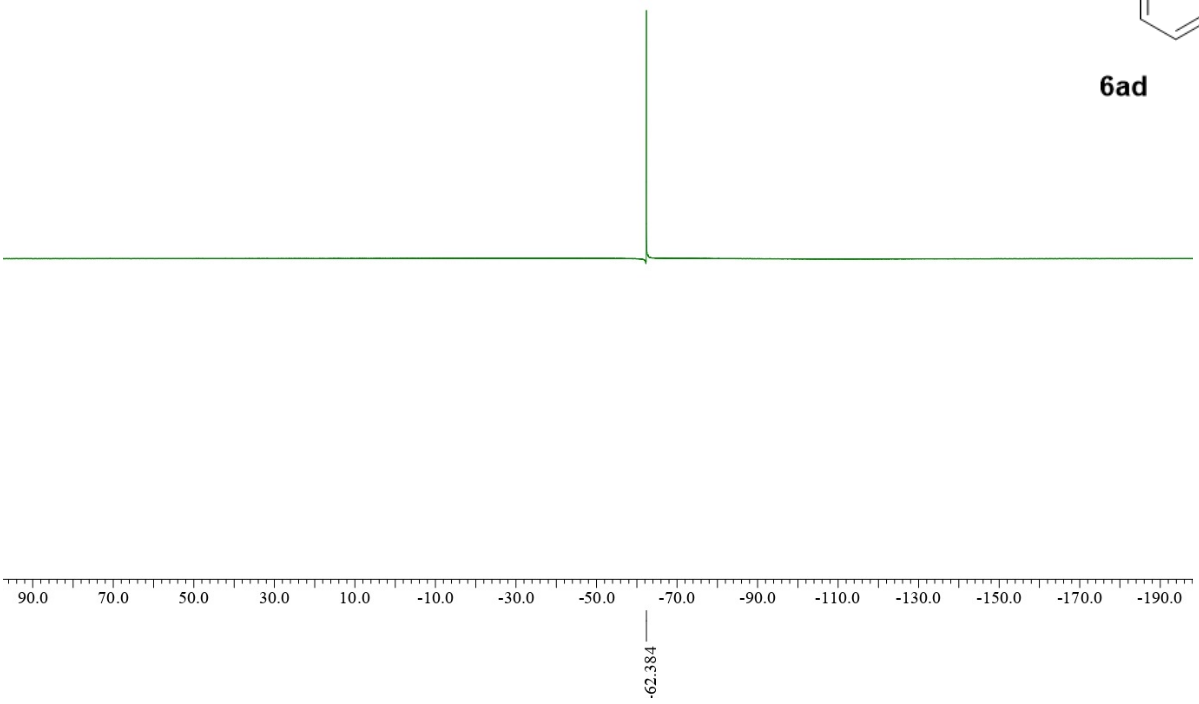

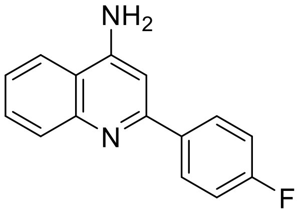

6ae

Figure S35. ^{1}H NMR of **6ae** (500 MHz, DMSO-d_6)

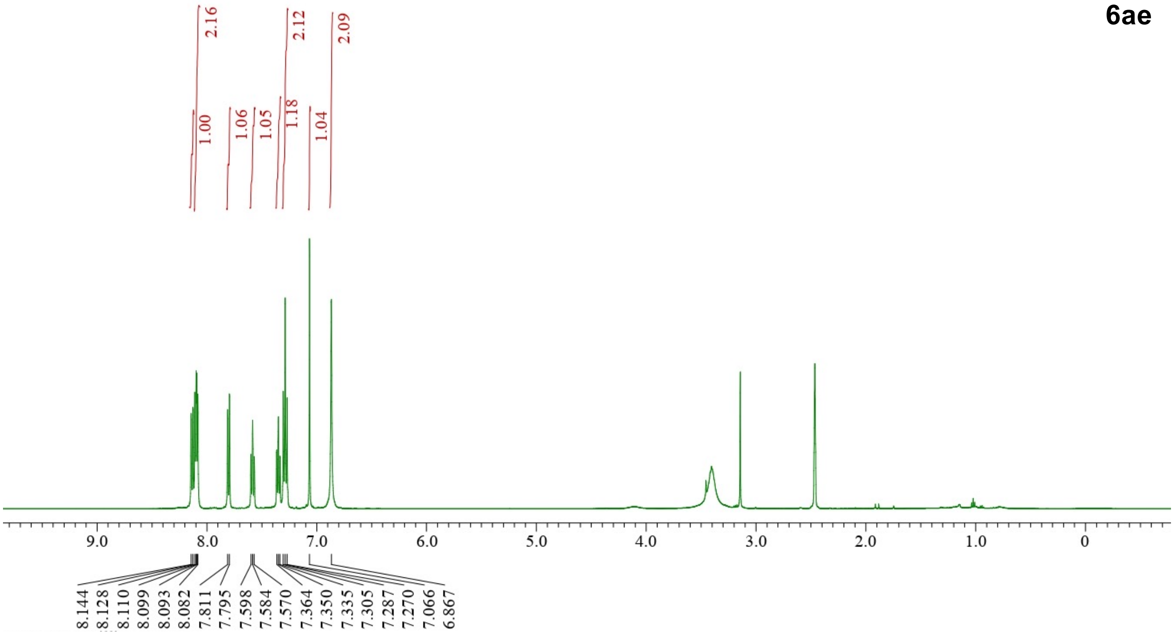

Figure S36. $^{13}C\{^{1}H\}$ NMR of **6ae** (125 MHz, DMSO-d_6)

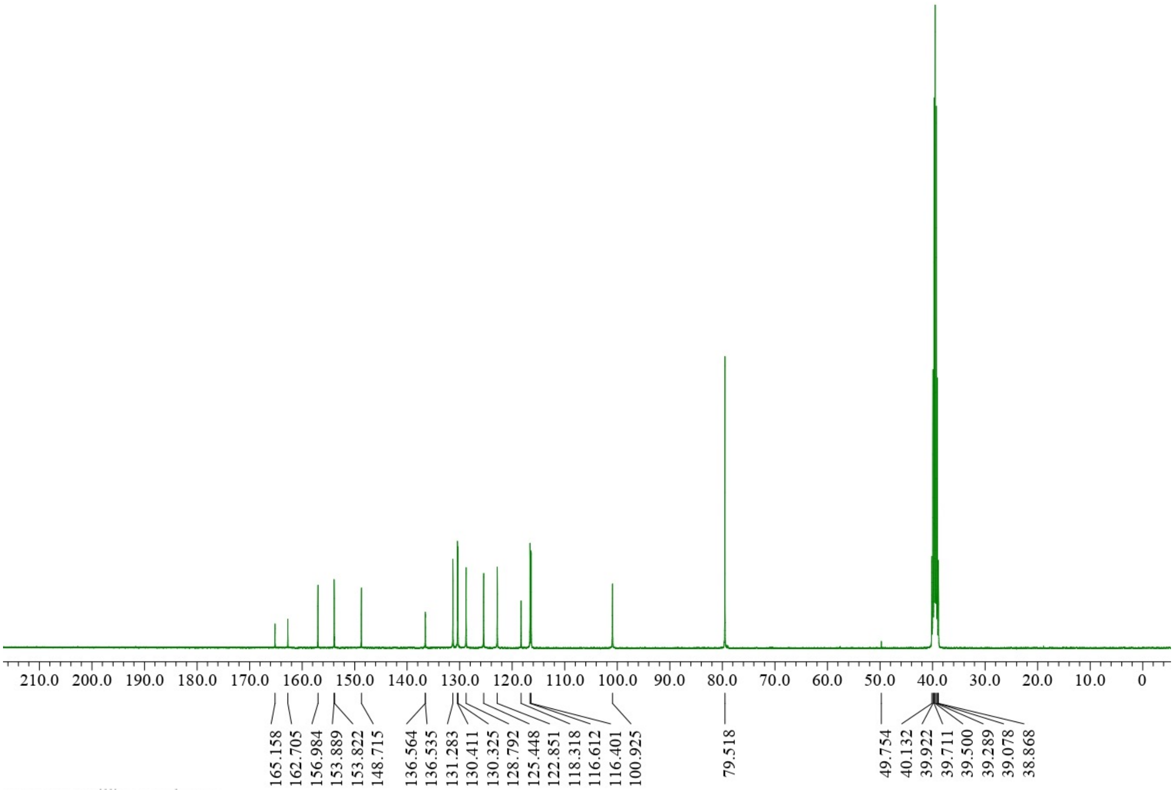

Figure S37. ^{19}F NMR of **6ae** (470 MHz, $CDCl_3$)

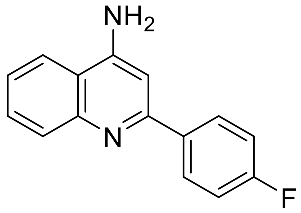

6ae

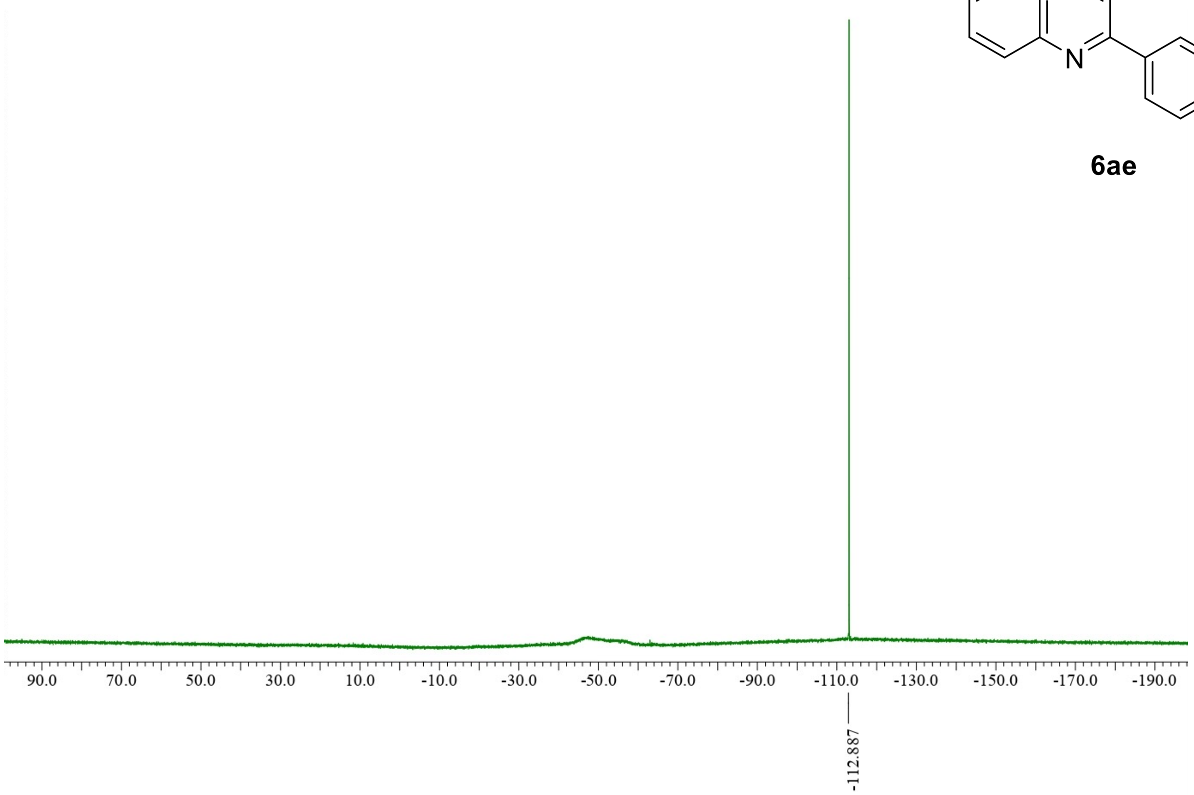

Figure S38. ^{1}H NMR of **6af** (500 MHz, DMSO-d_6)

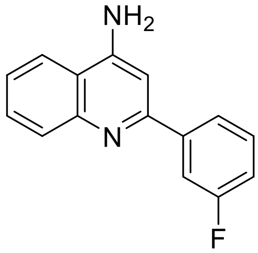

6af

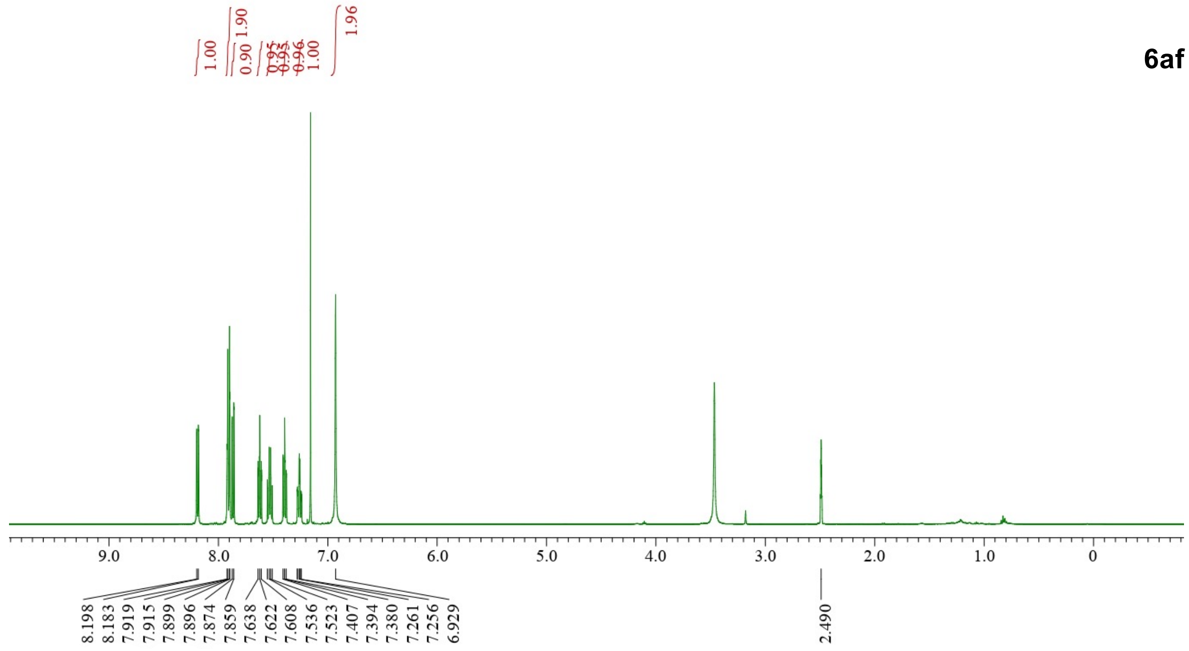

Figure S39. ^{13}C{^{1}H} NMR of **6af** (125 MHz, DMSO-d_6)

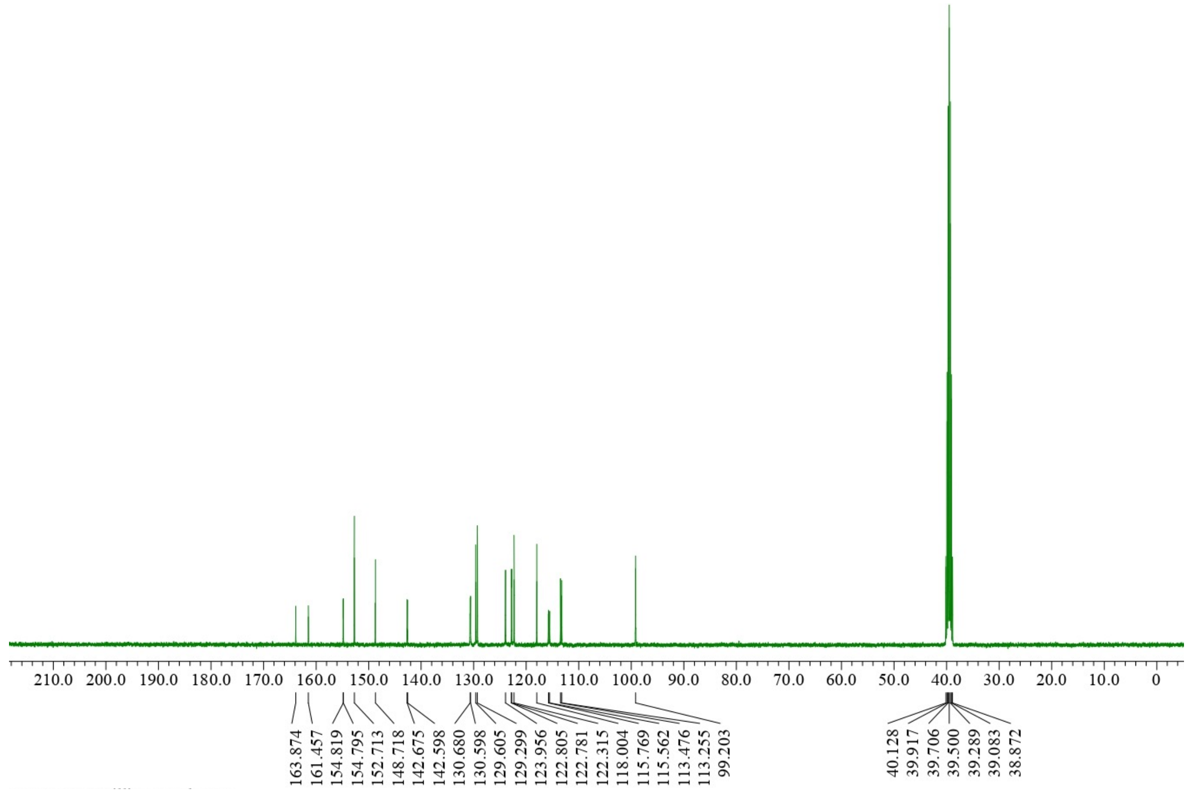

Figure S40. ^{19}F NMR of **6af** (470 MHz, $CDCl_3$)

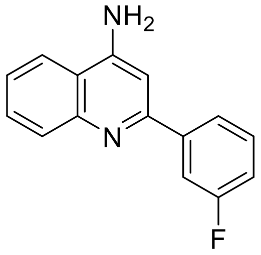

6af

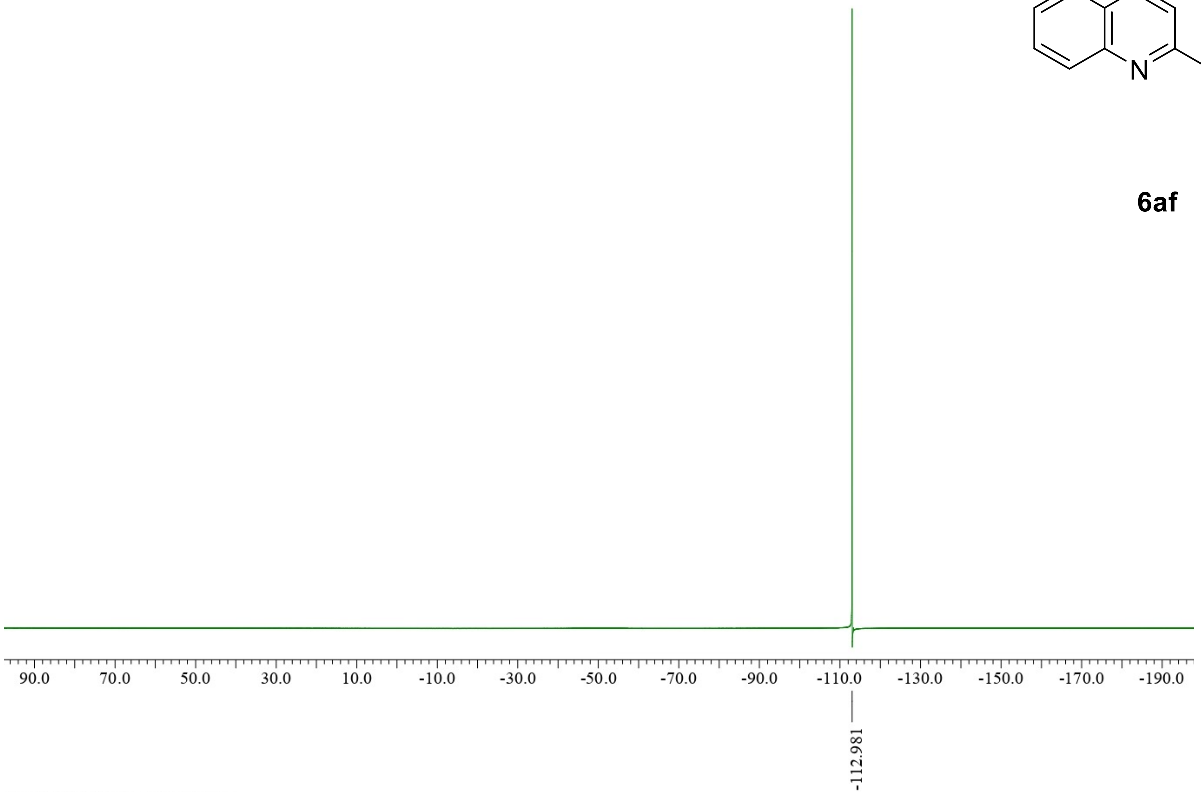

Figure S41. ^{1}H NMR of **6ag** (400 MHz, $CDCl_3$)

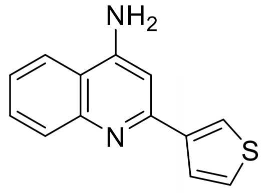

6ag

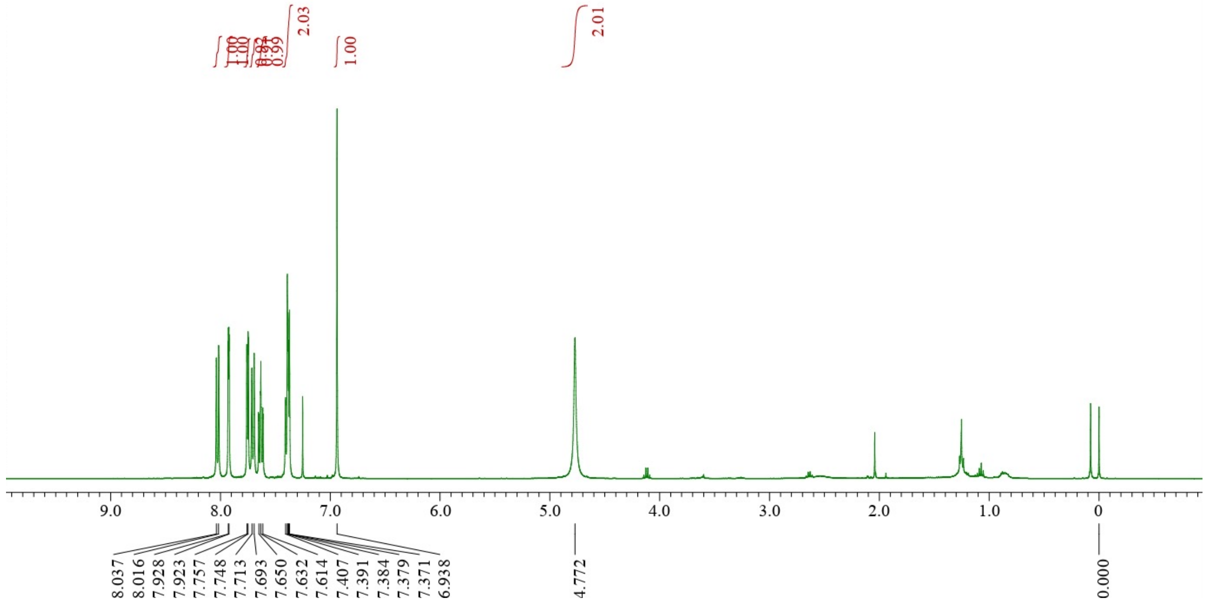

Figure S42. $^{13}C\{^{1}H\}$ NMR of **6ag** (100 MHz, $CDCl_3$)

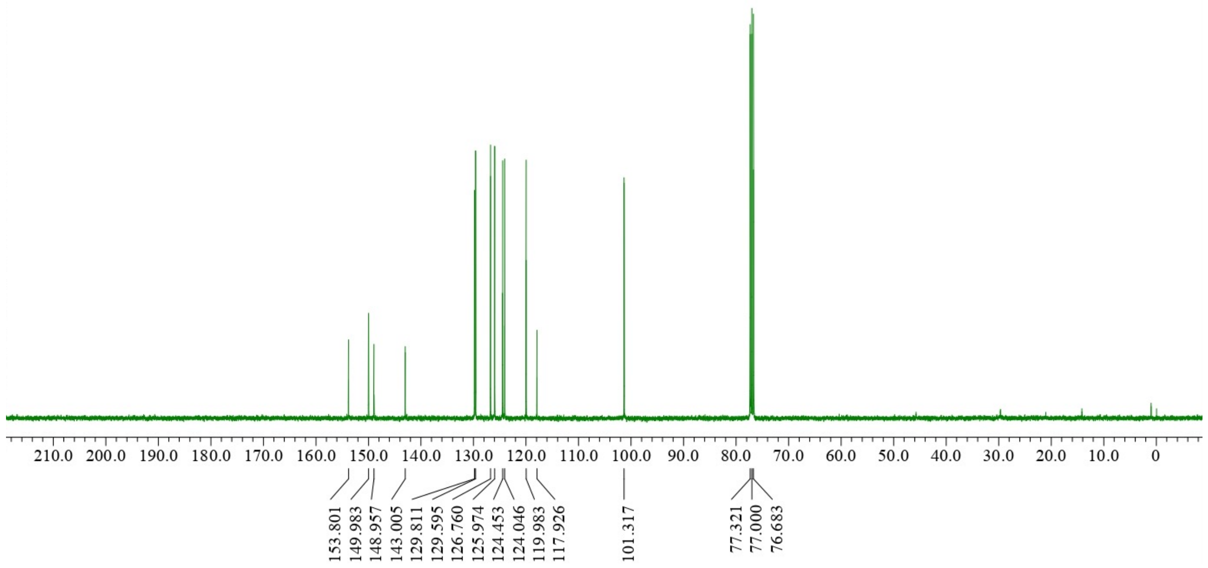

Figure S43. ^{1}H NMR of **6ka** (500 MHz, $CDCl_3$)

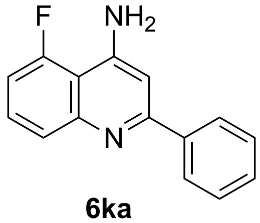

6ka

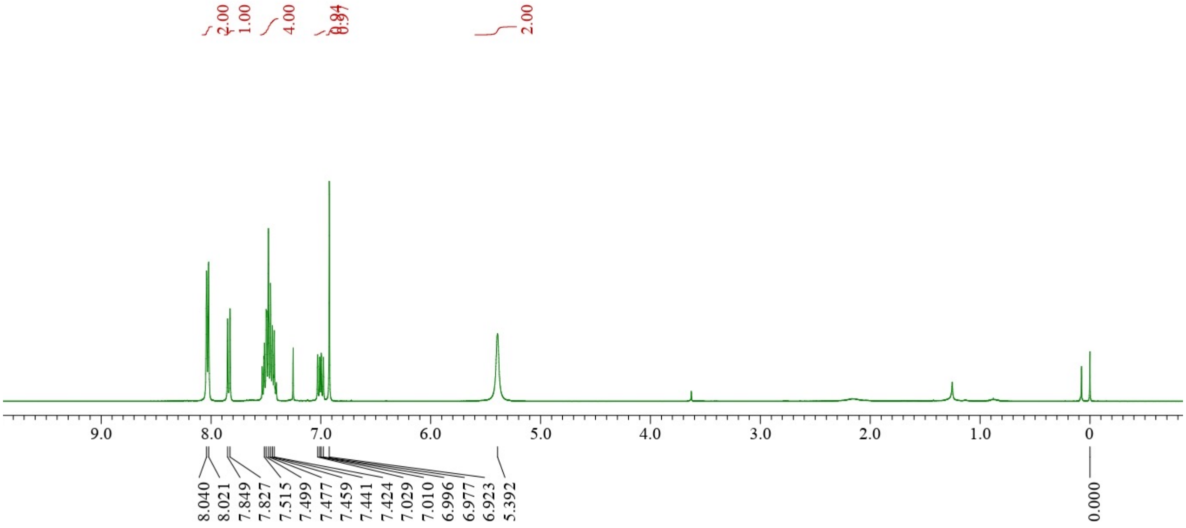

Figure S44. $^{13}C\{^{1}H\}$ NMR of **6ka** (125 MHz, $CDCl_3$)

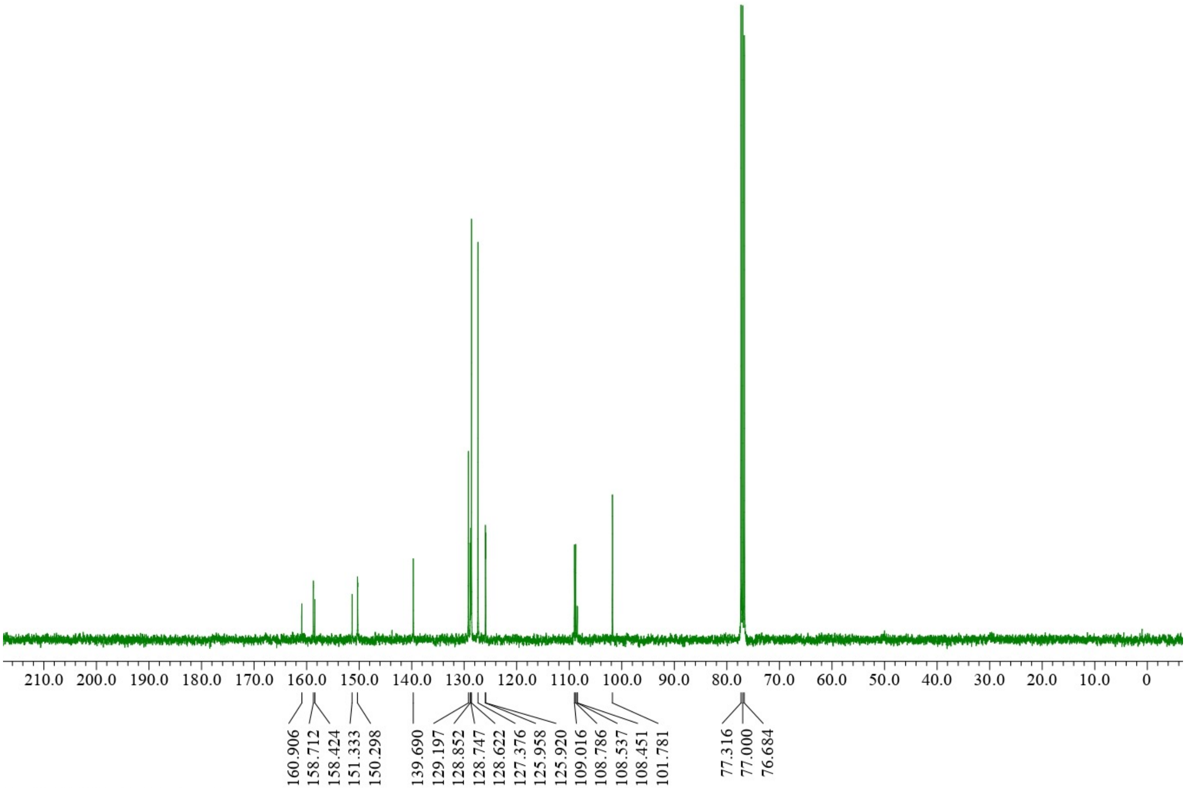

Figure S45. ^{19}F NMR of **6ha** (470 MHz, $CDCl_3$)

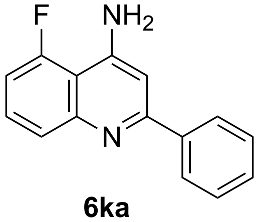

6ka

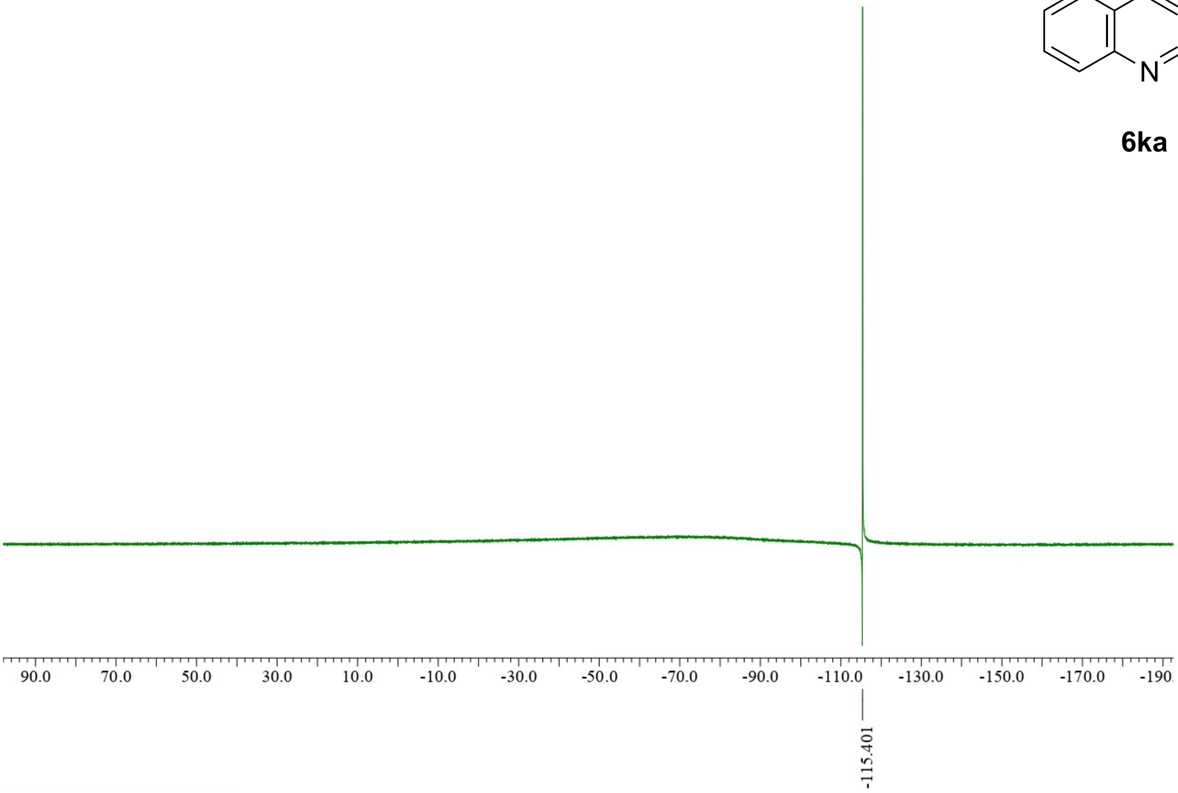

Figure S46. ^{1}H NMR of **8** (500 MHz, $CDCl_3$)

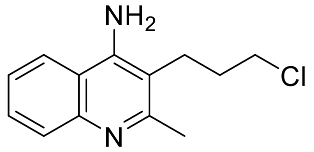

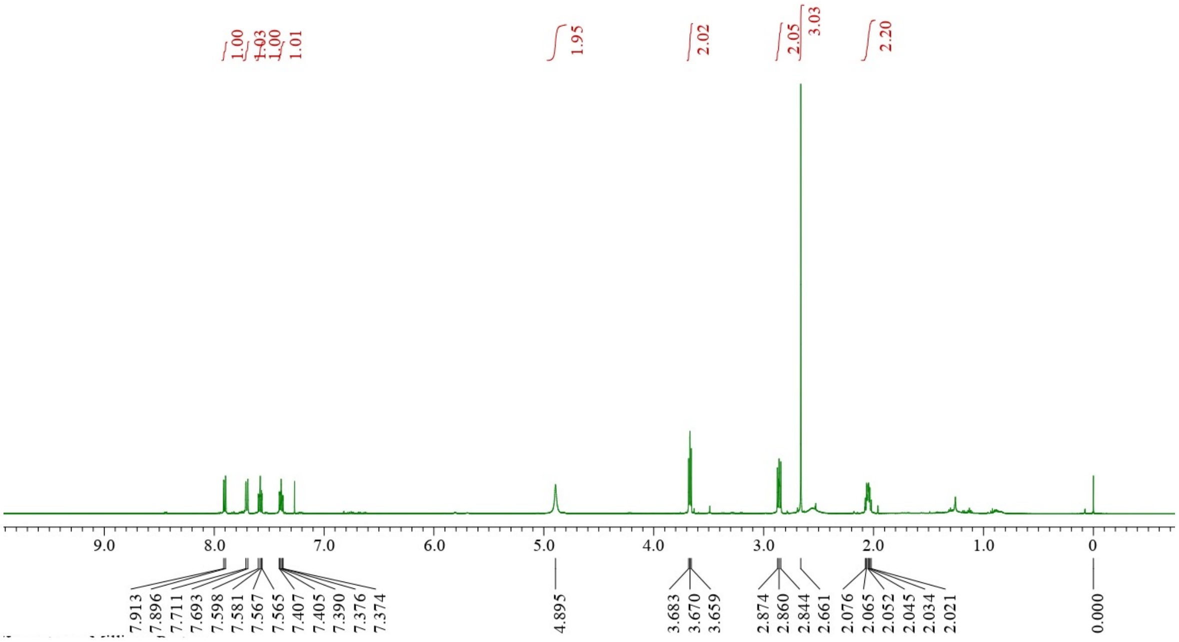

Figure S47. $^{13}C\{^1H\}$ NMR of **8** (125 MHz, $CDCl_3$)

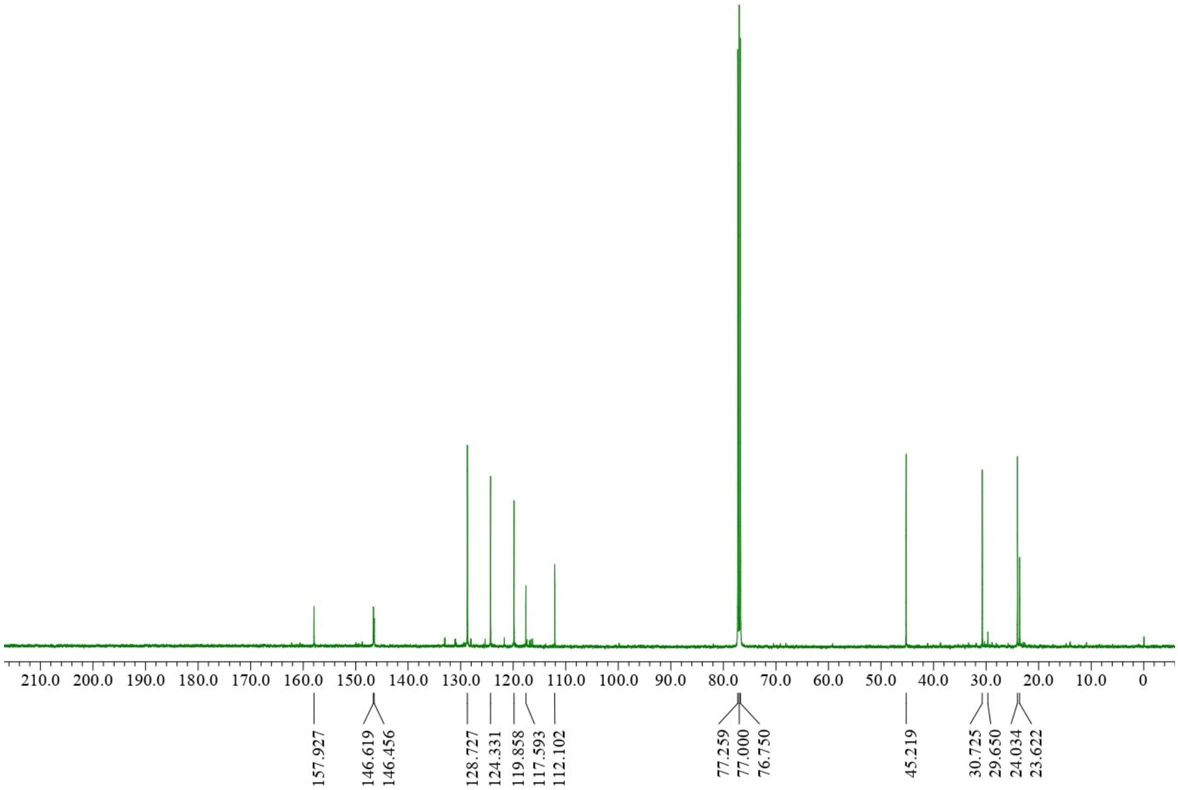

Figure S48. ^{1}H NMR of **9** (500 MHz, DMSO-d_6)

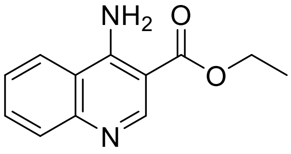

9

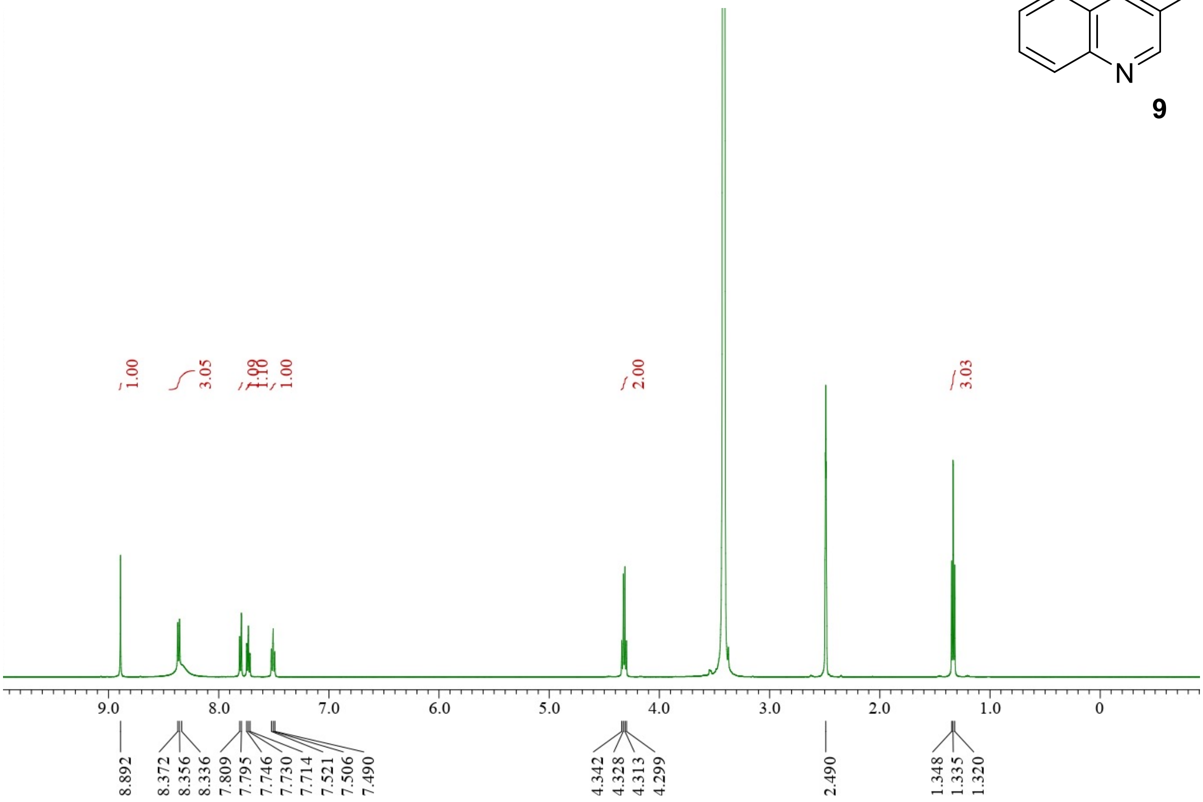

Figure S49. $^{13}C\{^{1}H\}$ NMR of **9** (125 MHz, DMSO-d_6)

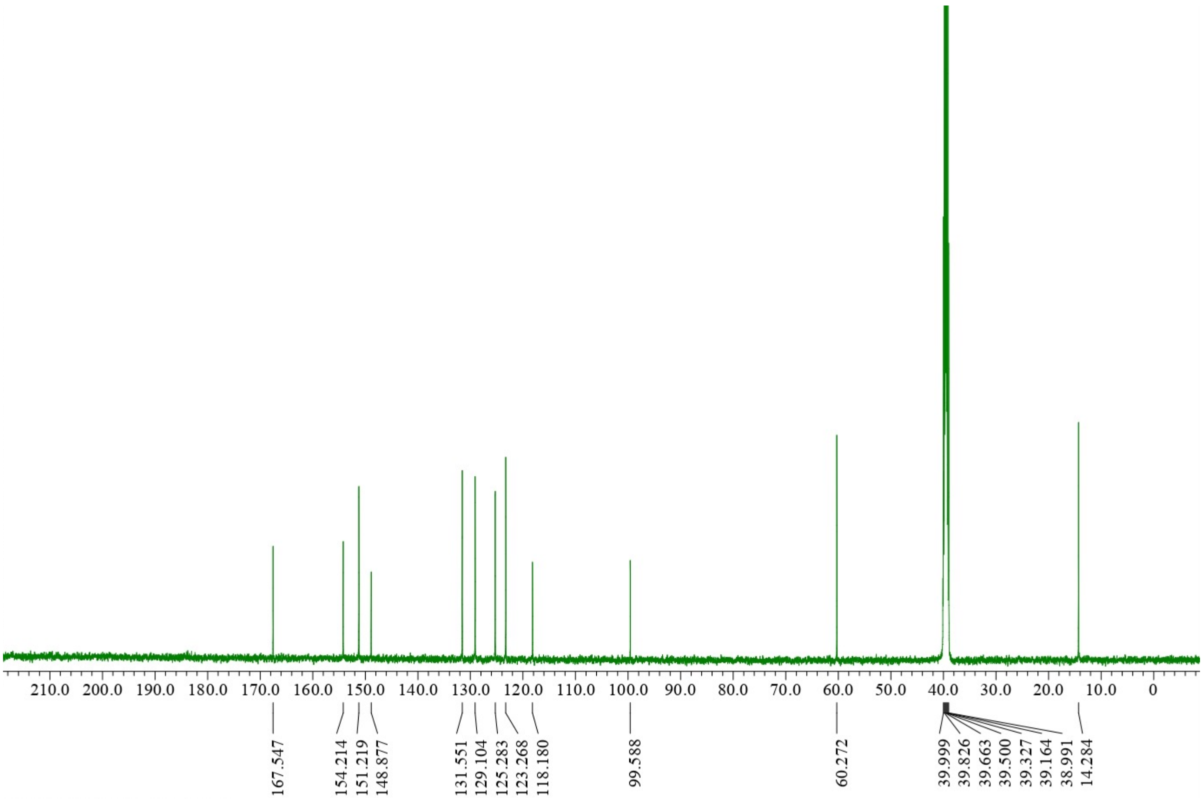

Figure S50. ^{1}H NMR of **10** (400 MHz, $CDCl_3$)

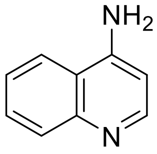

10

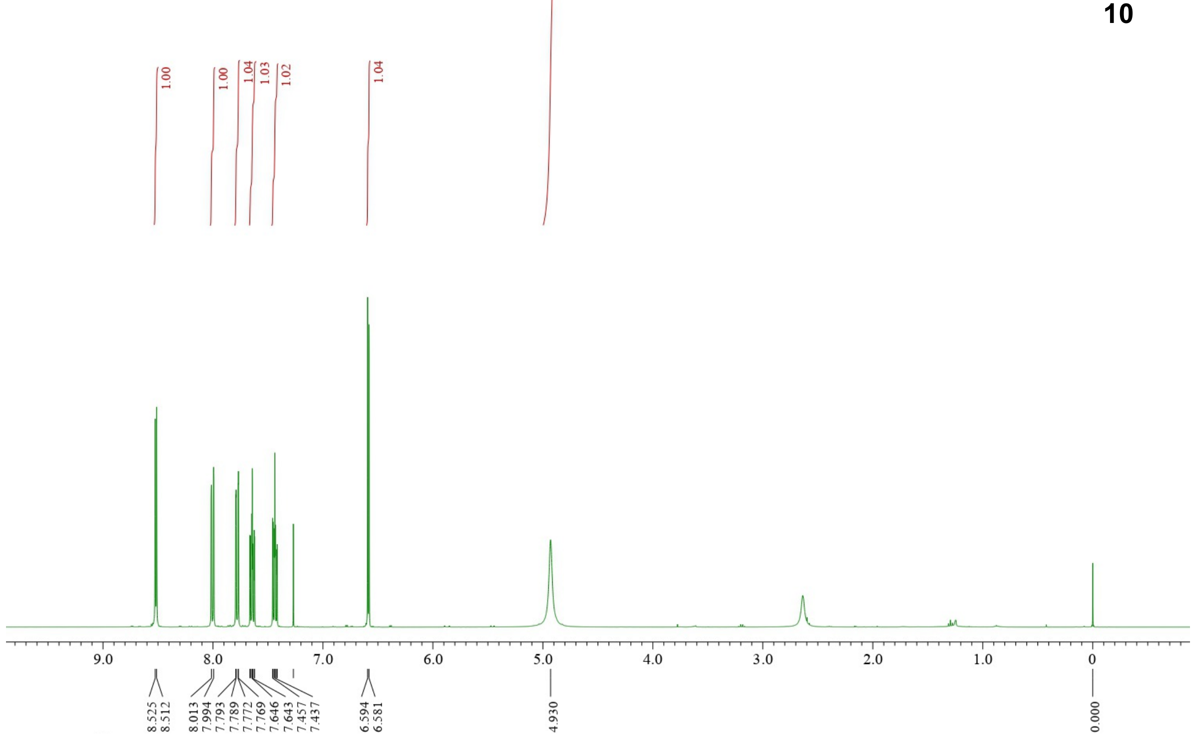

Figure S51. $^{13}C\{^{1}H\}$ NMR of **10** (100 MHz, $CDCl_3$)

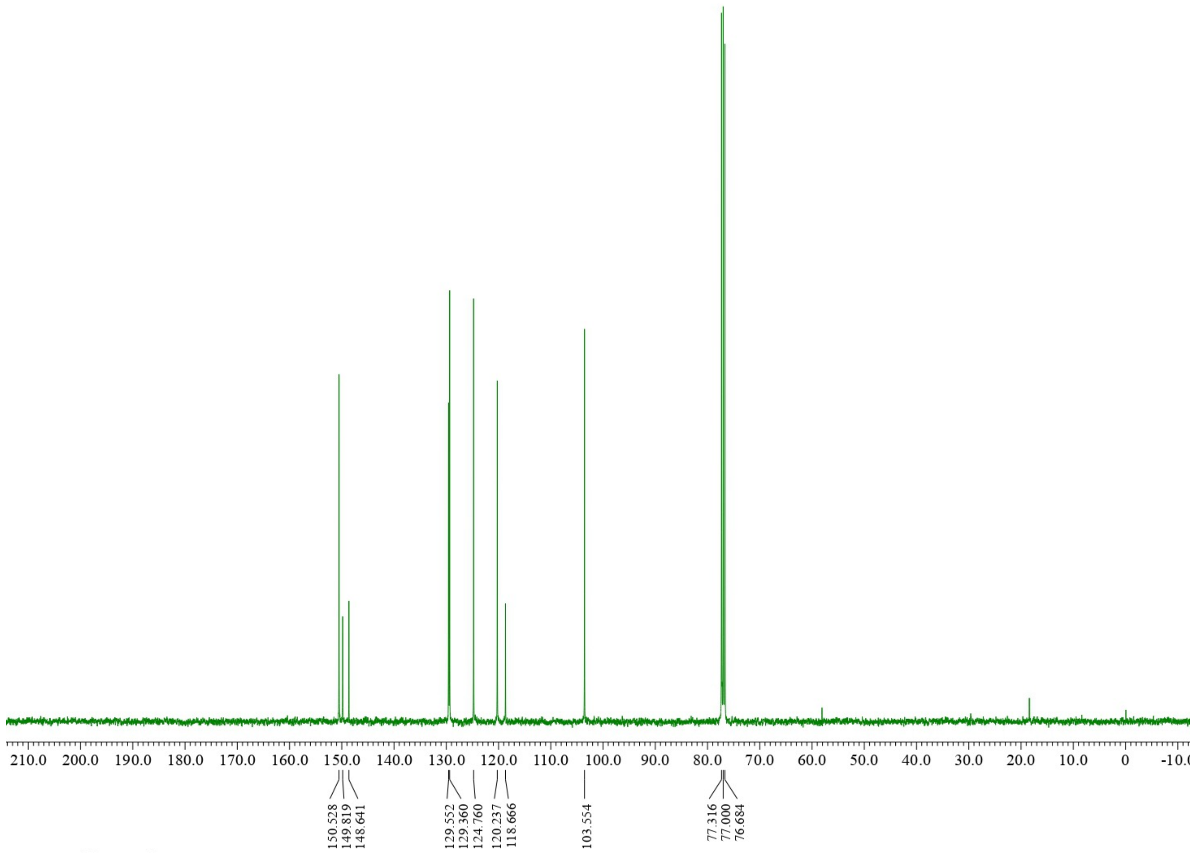

Figure S52. ^{1}H NMR of **11** (400 MHz, $CDCl_3$)

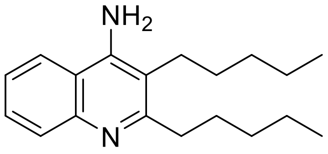

11

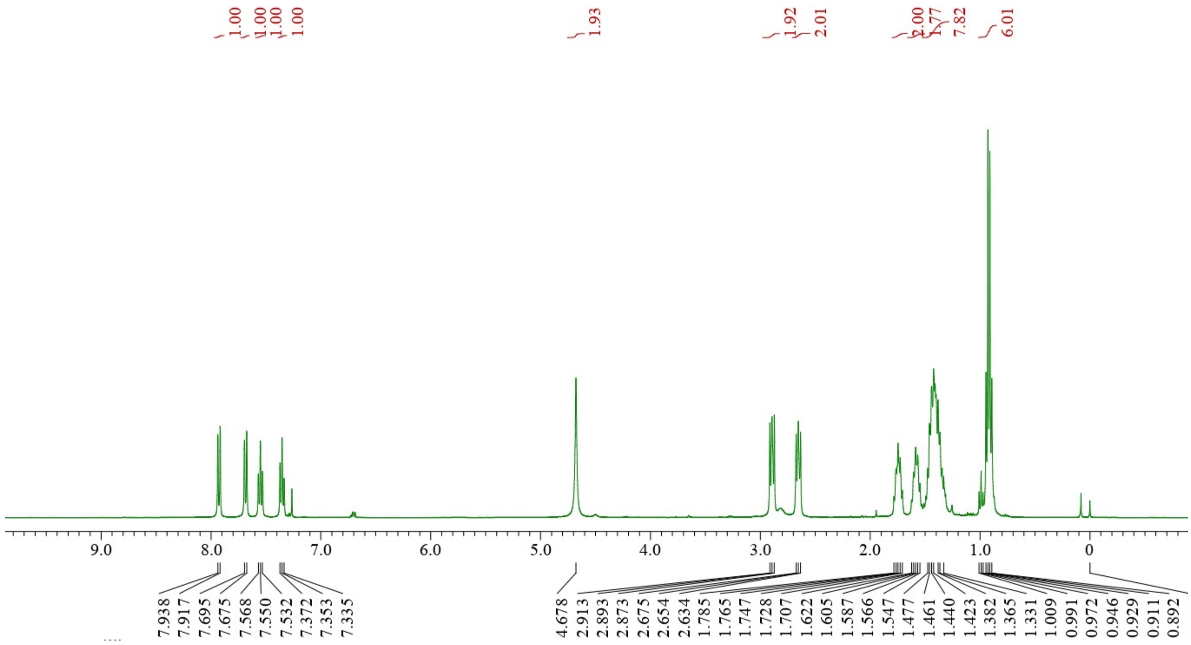

Figure S53. ^{13}C{^{1}H} NMR of **11** (100 MHz, $CDCl_3$)

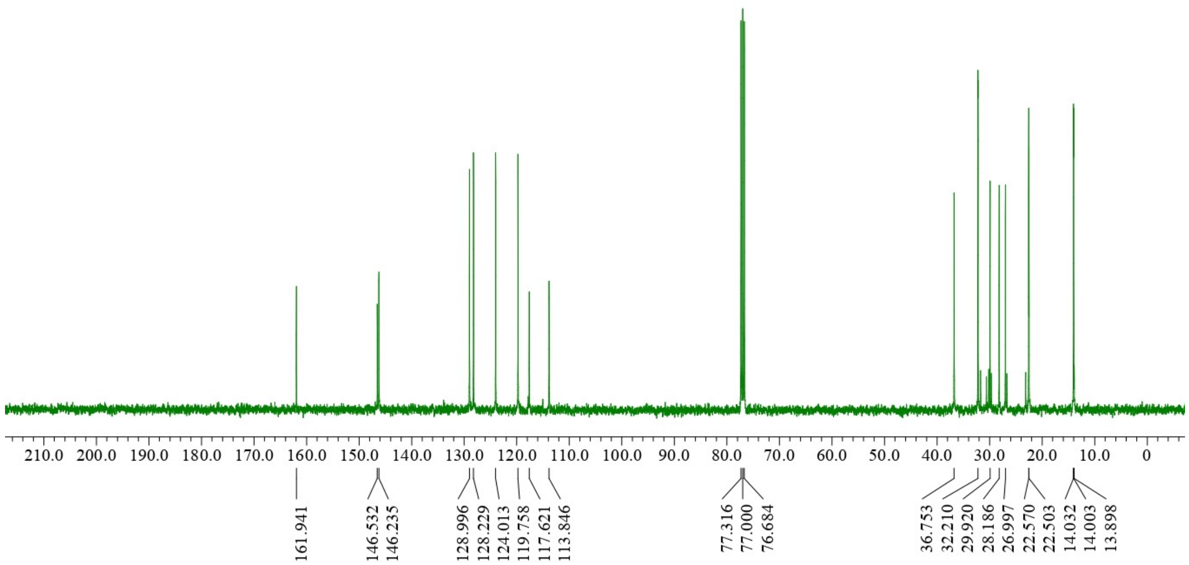

Figure S54. ^{1}H NMR of **12** (400 MHz, $CDCl_3$)

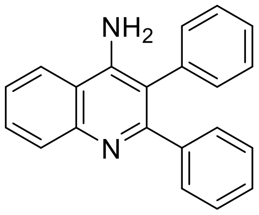

12

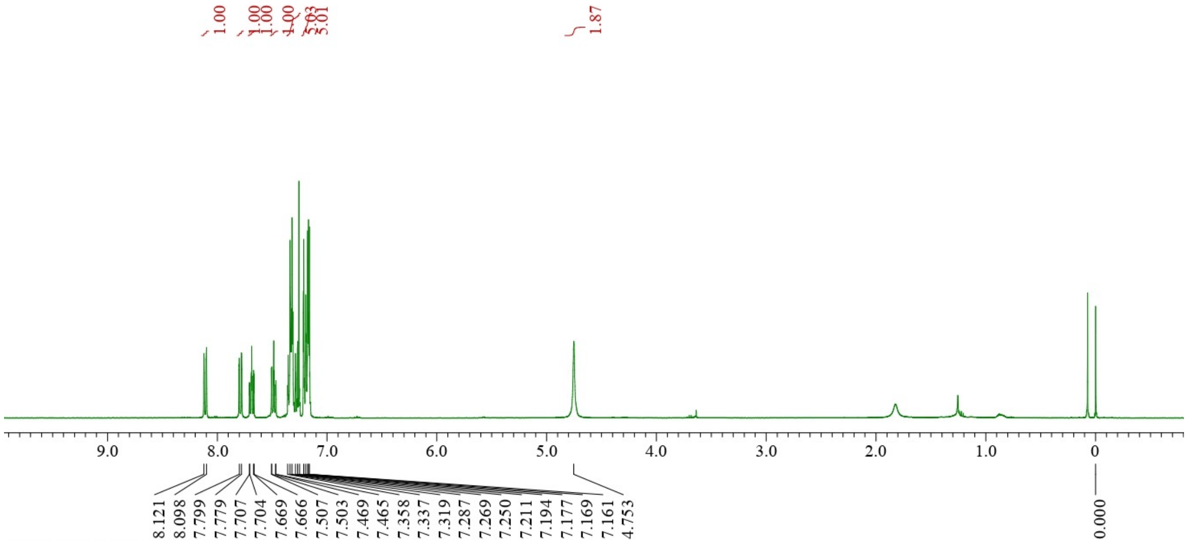

Figure S55. $^{13}C\{^{1}H\}$ NMR of **12** (100 MHz, $CDCl_3$)

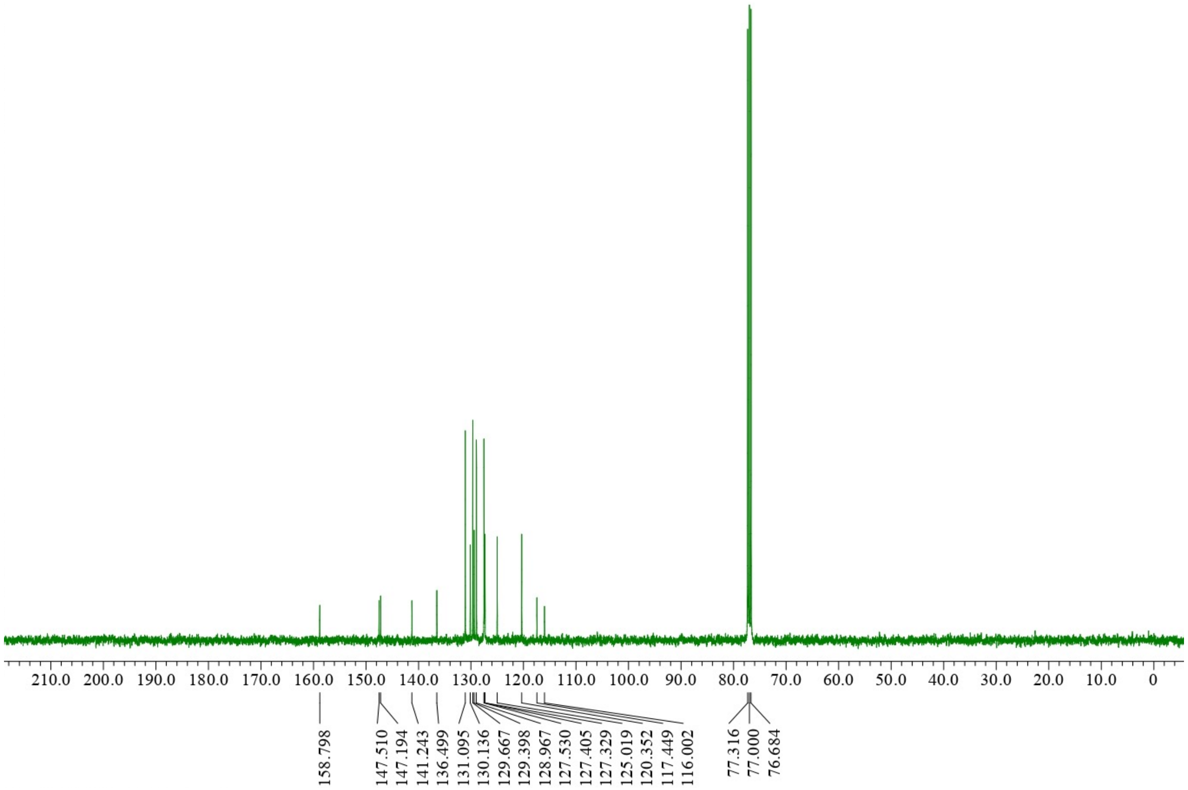

Figure S56. ^{1}H NMR of **13** (400 MHz, $CDCl_3$)

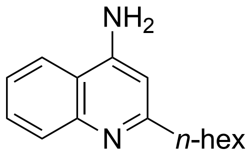

13

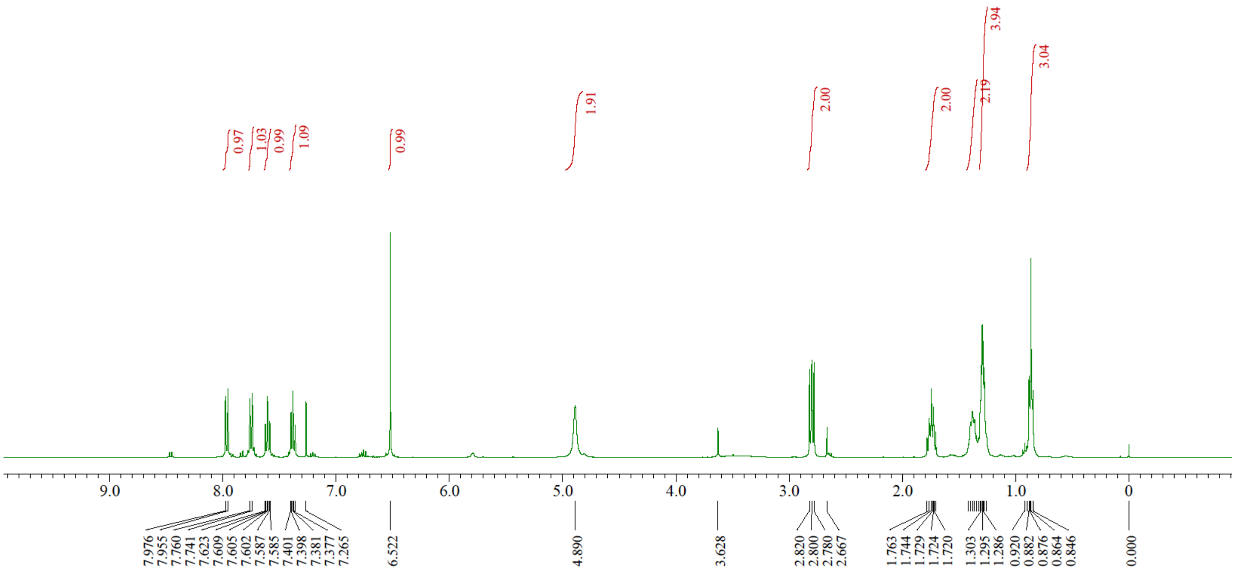

Figure S57. $^{13}C\{^{1}H\}$ NMR of **13**(100 MHz, $CDCl_3$)

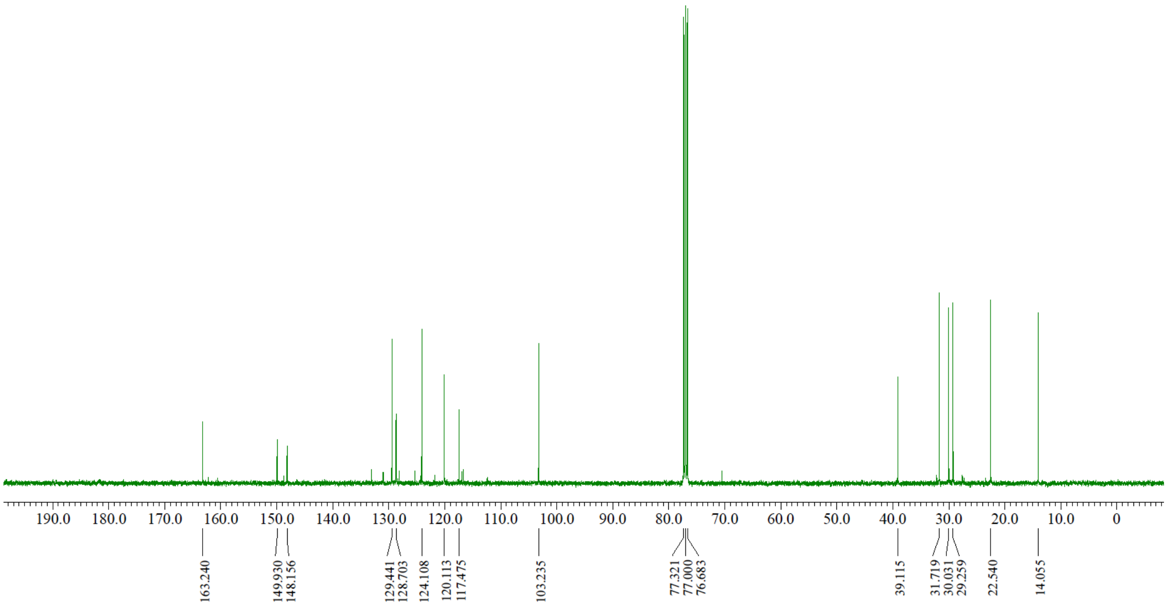

Figure S58. ^{1}H NMR of **14** (400 MHz, $CDCl_3$)

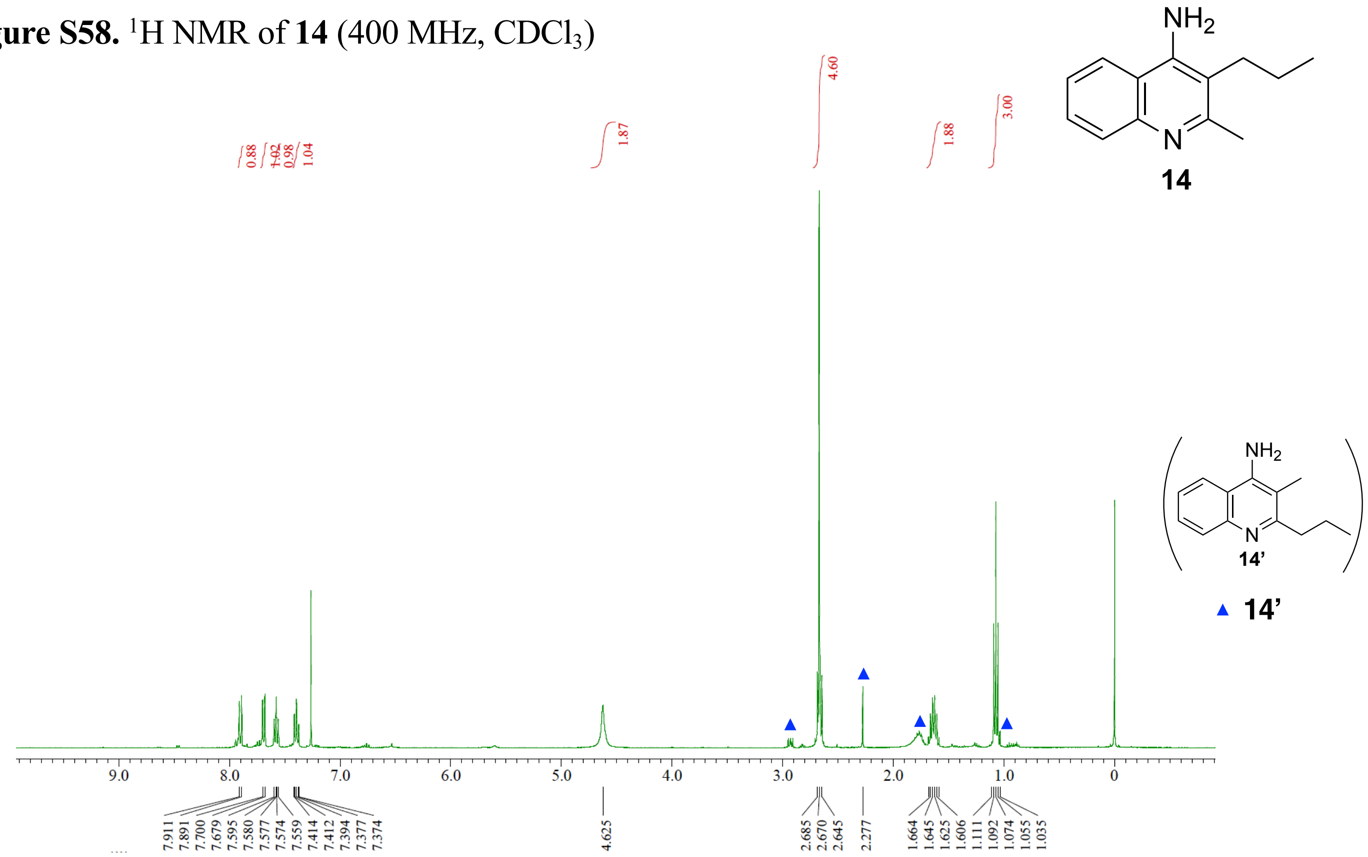

Figure S59. $^{13}C\{^{1}H\}$ NMR of **14** (100 MHz, $CDCl_3$)

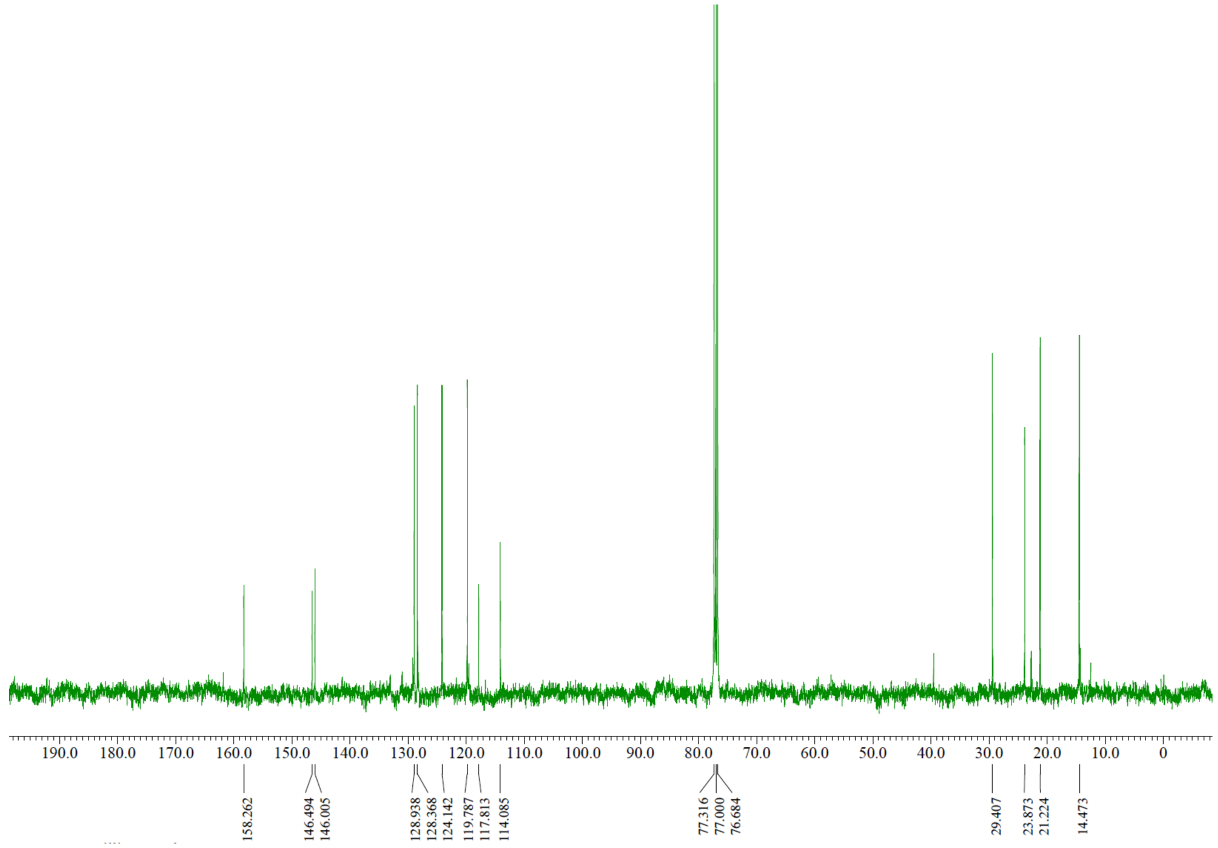

Figure S60. ^{1}H NMR of **16** (400 MHz, $CDCl_3$)

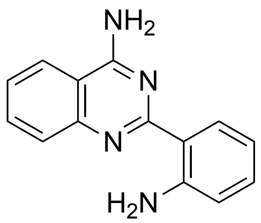

16

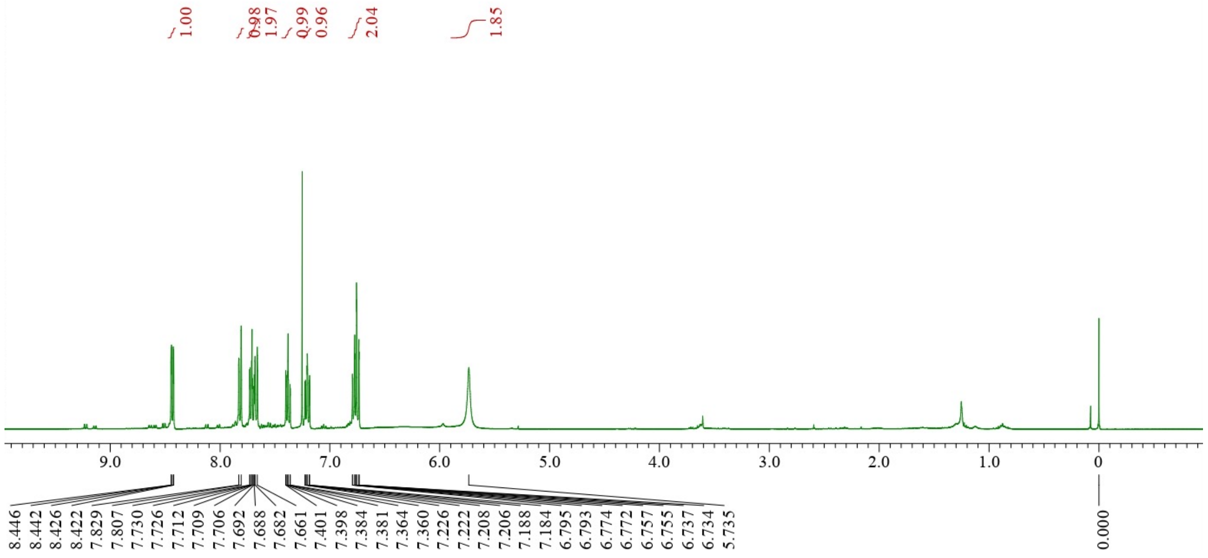

Figure S61. $^{13}C\{^{1}H\}$ NMR of **16** (100 MHz, $CDCl_3$)

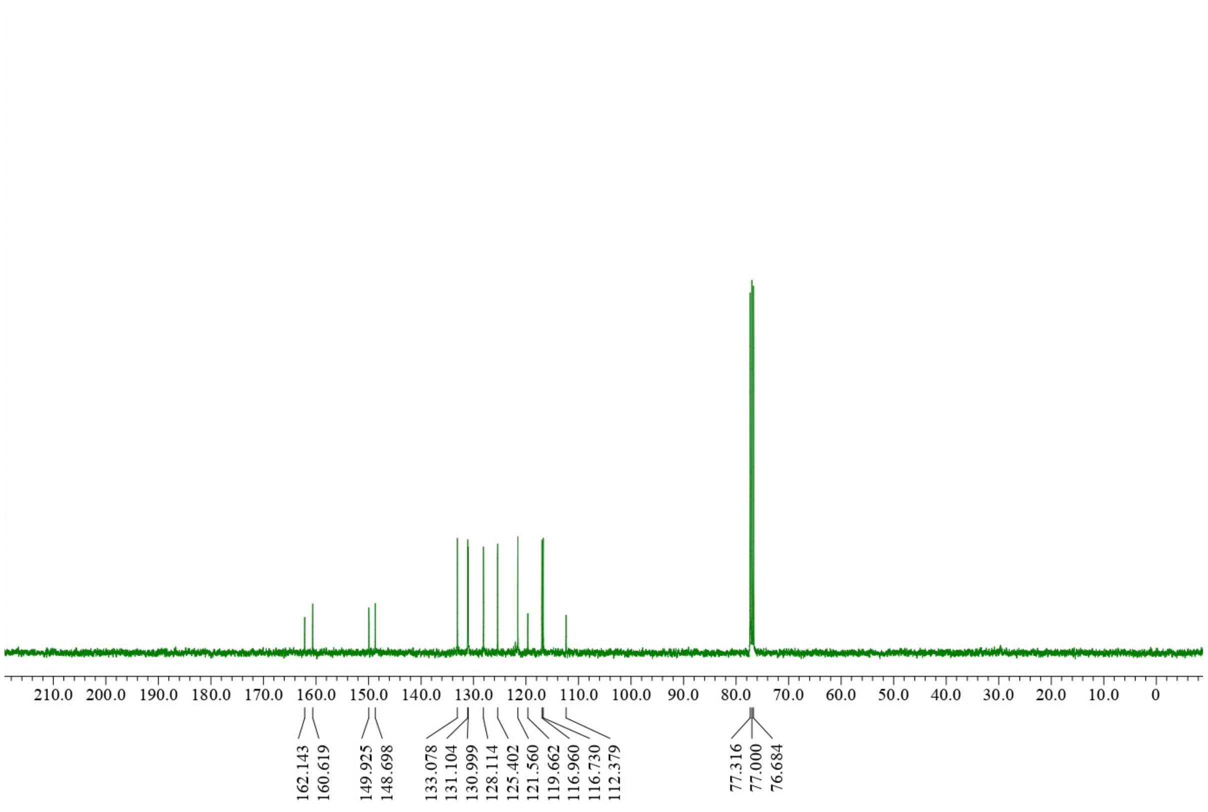

Figure S62. ^{1}H NMR of **17** (400 MHz, DMSO-d_6)

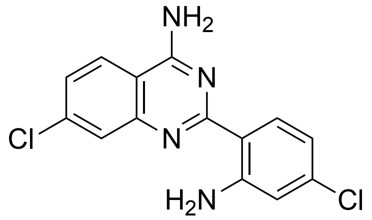

17

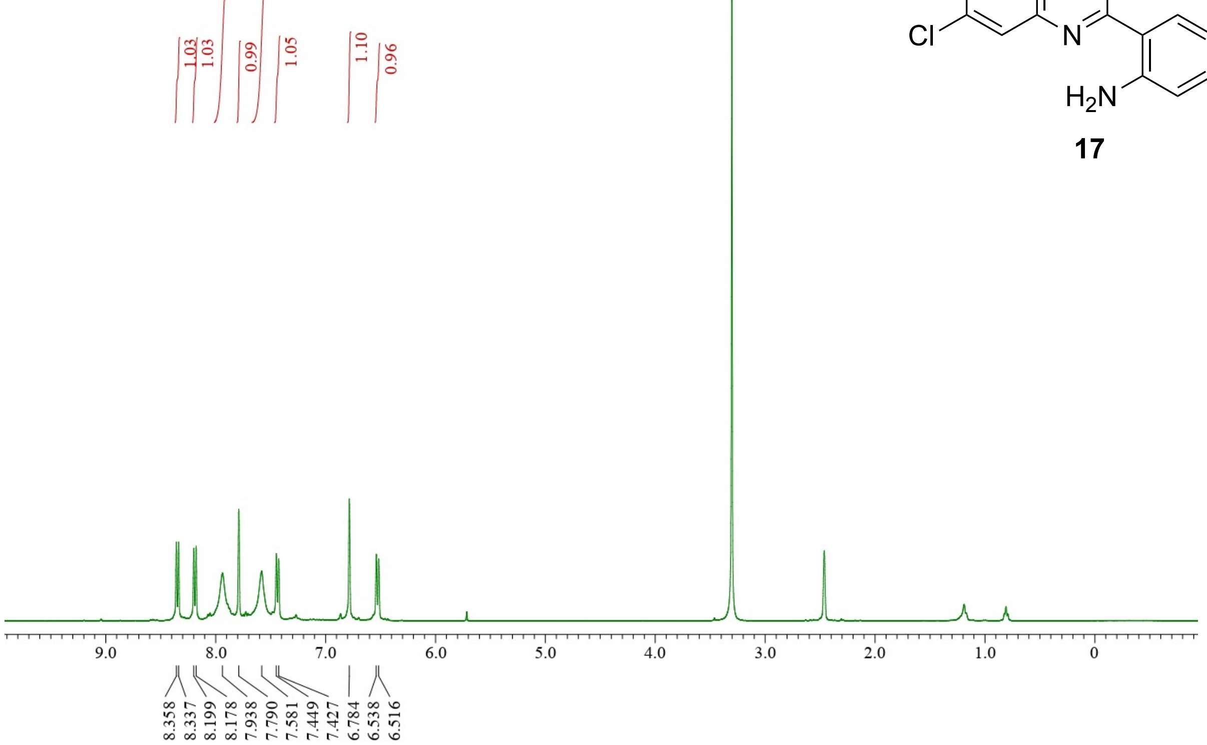

Figure S63. ^{13}C{^{1}H} NMR of **17** (100 MHz, DMSO-d_6)

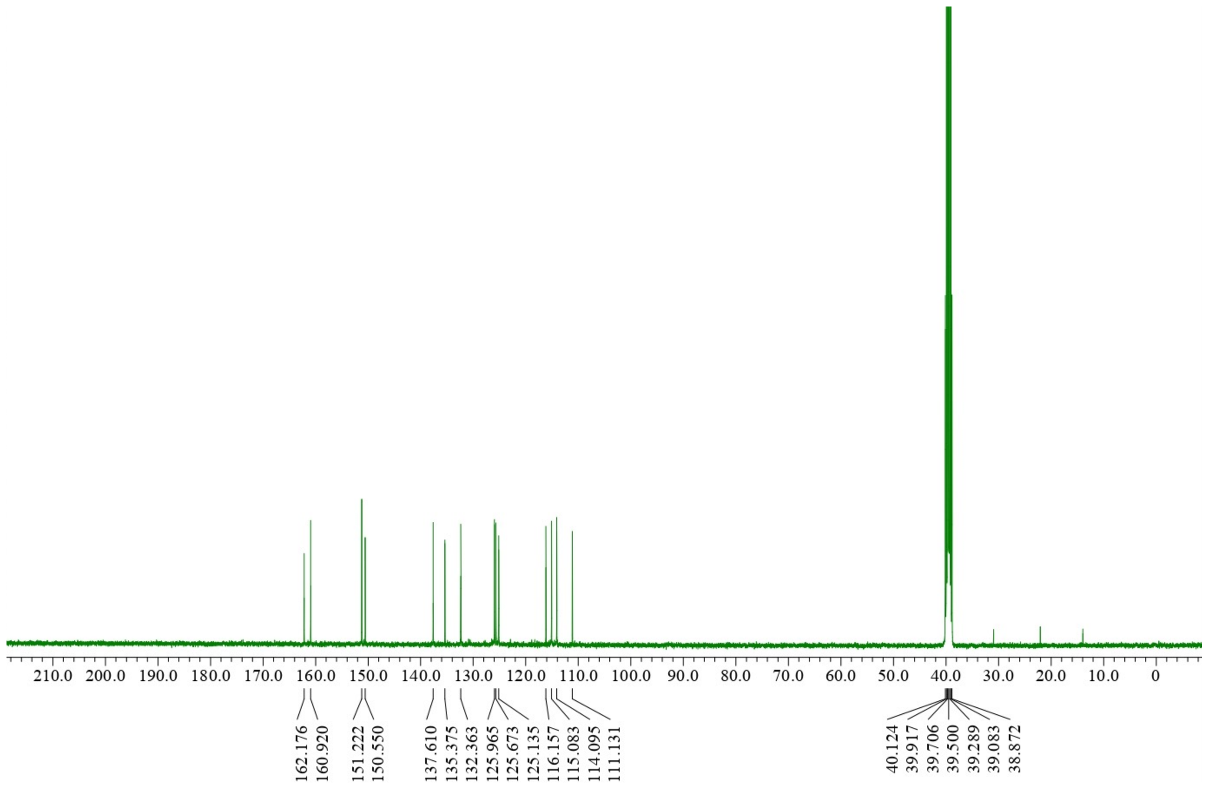

Supplement: Supplementary file 1 [file ao6c01857_si_001.pdf]
